# Novel meriolin derivatives potently inhibit cell cycle progression and transcription in leukemia and lymphoma cells via inhibition of cyclin-dependent kinases (CDKs)

**DOI:** 10.1038/s41420-024-02056-6

**Published:** 2024-06-11

**Authors:** Laura Schmitt, Julia Hoppe, Pablo Cea-Medina, Peter-Martin Bruch, Karina S. Krings, Ilka Lechtenberg, Daniel Drießen, Christoph Peter, Sanil Bhatia, Sascha Dietrich, Björn Stork, Gerhard Fritz, Holger Gohlke, Thomas J. J. Müller, Sebastian Wesselborg

**Affiliations:** 1 Institute for Molecular Medicine I, Medical Faculty and University Hospital Düsseldorf, Heinrich Heine University Düsseldorf, Universitätsstraße 1, 40225 Düsseldorf, Germany; 2 Institute for Pharmaceutical and Medicinal Chemistry, Faculty of Mathematics and Natural Sciences, Heinrich Heine University Düsseldorf, Universitätsstraße 1, 40225 Düsseldorf, Germany; 3 Department of Hematology, Oncology and Clinical Immunology, Medical Faculty and University Hospital Düsseldorf, Heinrich Heine University Düsseldorf, Moorenstraße 5, 40225 Düsseldorf, Germany; 4 Department of Medicine V, Heidelberg University Hospital, Heidelberg, Germany; 5 Molecular Medicine Partnership Unit (MMPU), Heidelberg, Germany; 6 Center for Integrated Oncology Aachen-Bonn-Cologne-Düsseldorf (CIO ABCD), Düsseldorf, Germany; 7 Institute of Organic Chemistry and Macromolecular Chemistry, Faculty of Mathematics and Natural Sciences, Heinrich Heine University Düsseldorf, Universitätsstraße 1, 40225 Düsseldorf, Germany; 8 Department of Pediatric Oncology, Hematology and Clinical Immunology, Medical Faculty and University Hospital Düsseldorf, Heinrich Heine University Düsseldorf, Moorenstraße 5, 40225 Düsseldorf, Germany; 9 Institute of Toxicology, Medical Faculty and University Hospital Düsseldorf, Heinrich Heine University Düsseldorf, Universitätsstraße 1, 40225 Düsseldorf, Germany; 10 John von Neumann Institute for Computing (NIC), Jülich Supercomputing Center (JSC) and Institute of Bio- and Geosciences (IBG-4: Bioinformatics), Forschungszentrum Jülich GmbH, Wilhelm-Johnen-Straße, 52425 Jülich, Germany

**Keywords:** Drug development, Pharmaceutics, Apoptosis, Checkpoints, Drug development

## Abstract

A key feature of cancer is the disruption of cell cycle regulation, which is characterized by the selective and abnormal activation of cyclin-dependent kinases (CDKs). Consequently, targeting CDKs via meriolins represents an attractive therapeutic approach for cancer therapy. Meriolins represent a semisynthetic compound class derived from meridianins and variolins with a known CDK inhibitory potential. Here, we analyzed the two novel derivatives meriolin 16 and meriolin 36 in comparison to other potent CDK inhibitors and could show that they displayed a high cytotoxic potential in different lymphoma and leukemia cell lines as well as in primary patient-derived lymphoma and leukemia cells. In a kinome screen, we showed that meriolin 16 and 36 prevalently inhibited most of the CDKs (such as CDK1, 2, 3, 5, 7, 8, 9, 12, 13, 16, 17, 18, 19, 20). In drug-to-target modeling studies, we predicted a common binding mode of meriolin 16 and 36 to the ATP-pocket of CDK2 and an additional flipped binding for meriolin 36. We could show that cell cycle progression and proliferation were blocked by abolishing phosphorylation of retinoblastoma protein (a major target of CDK2) at Ser612 and Thr82. Moreover, meriolin 16 prevented the CDK9-mediated phosphorylation of RNA polymerase II at Ser2 which is crucial for transcription initiation. This renders both meriolin derivatives as valuable anticancer drugs as they target three different Achilles’ heels of the tumor: (1) inhibition of cell cycle progression and proliferation, (2) prevention of transcription, and (3) induction of cell death.

## Introduction

A fundamental aspect of cancer is the dysregulation of cell cycle control, which is associated with selective, aberrant activation of cyclin-dependent kinases (CDKs). The CDK family consists of 21 CDKs, with different functions in cell cycle and gene transcription [1, 2]. CDK1 and CDK2 are responsible for the cell cycle, whereas CDK7, 8, and 9 control transcription by phosphorylating and activating RNA polymerase II [1, 3, 4]. Accordingly, inhibition of CDKs represents an attractive therapeutical strategy in cancer therapy.

The cell cycle consists of four phases: G_1_ phase, S phase, G_2_ phase and M phase (mitosis and cytokinesis) [5, 6]. Cell cycle progression is regulated by (1) CDKs, (2) their respective cyclins, (3) interconnected transcriptional complexes (i.e., E2F), (4) the Aurora kinase family and (5) kinases of the anaphase-promoting complex/cyclosome (APC/C; multiprotein E3 ubiquitin ligase complex targeting mitotic proteins for degradation and promoting anaphase) [6, 7]. The interconnected network of transcription factors (E2F, B-MYB, forkhead box protein M1 (FOXM1) and retinoblastoma (RB) protein) and CDKs with their respective cyclins regulate each other by feedback loops, which collectively constitute an almost fail-save system for cell cycle progression [6]. Two key regulators—cyclins and CDKs—determine the cell’s progress through the cell cycle [8, 9]. Cyclins comprise the regulatory and CDKs the catalytic subunits of an activated heterodimer. The cyclins have no catalytic activity and CDKs are inactive in the absence of their cyclin partner [9]. CDKs get activated by binding to their cyclin partner in order to phosphorylate their target proteins (e.g., RB protein or RNA polymerase II). The E2F suppressor RB plays an important role in the cell cycle, in particular in the decision to re-enter a new cycle [10, 11]. The RB protein exists mainly in two major phosphorylation states. CDK4/6/cyclin D can monophosphorylate RB on any of its 14 known phosphosites, whereas CDK1 and CDK2 are the main contributors to the multi- or hyperphosphorylation of RB and its inactivation (a comprehensive overview is provided in Supplementary Fig. 1).

Cancer cells exhibit increased cell division and depend on cell cycle control mechanisms to compensate for the excessive accumulation and propagation of genomic instability [5]. CDK inhibitors for tumor therapy have been studied since the early 1990s, and many compounds exhibiting specific or pan-CDK activity have been developed and analyzed in clinical trials. Broad-spectrum CDK inhibitors such as flavopiridol or roscovitine impede the cell cycle and repress growth, but their major drawback is their lack of specificity, leading to elevated toxicity and serious side effects [12]. Consequently, they displayed limited success in clinical trials [13–15].

Meriolins represent a chemical hybrid between the natural products meridianins (a family of 3-((2-amino) pyrimidin-4-yl) indoles) and variolins (containing a central pyrido[3’,2’:4,5]pyrrolo[1,2-c]pyridine core substituted with a 2-aminopyrimidine ring) (Supplementary Fig. 2A, B) [16–19]. Meridianins and variolins exhibit inhibitory activity against CDKs in micro- to nanomolar range [16, 17, 20, 21]. So far, the biological effects of numerous meriolin derivatives have been documented, including their cytotoxicity, kinase inhibition, and ability to inhibit tumor growth [16, 17, 19, 21–26]. Chemical derivatization led to higher selectivity and improved anti-proliferative and pro-apoptotic properties compared to their original compounds [27].

In a previous study, we showed that two novel meriolin derivatives (meriolin 16 and meriolin 36) were able to activate the mitochondrial apoptosis pathway even in the presence of antiapoptotic Bcl-2 protein. Meriolin 16 and meriolin 36 potently induced apoptosis at nanomolar concentrations and were able to induce cell death in imatinib-resistant K562 chronic myeloid leukemia (CML) cells, cisplatin-resistant J82 urothelial carcinoma cells and cisplatin-resistant 2102EP germ cell tumor cells [28].

In the present study, we were interested to unravel the kinase inhibition profile, binding mode to CDK1, 2 and 9, effect on cell cycle and proliferation, and transcriptional activity of meriolin 16 and 36. Meriolin 16 was synthesized with an additional methoxy group at the aromatic pyridine ring in order to enhance its cytotoxicity compared to its parental compound meriolin 31 (Supplementary Fig. 2B) [22]. In order to evaluate the cytotoxic potential, we compared meriolin 16 and 36 with established CDK inhibitors (dinaciclib, flavopiridol, meriolin 3, R547, roscovitine, SNS-032, and zotiraciclib), some of which are presently undergoing clinical trials (Supplementary Fig. 2C). Thus, we could show that meriolin 16 and 36 displayed a strong cytotoxic potential in different leukemia and lymphoma cell lines (HL60, HPBALL, Jurkat, K562, KOPTK1, MOLT4, Ramos, and SUPB15) as well as in primary patient-derived lymphoma and leukemia cells. Especially, the novel derivative meriolin 16 displayed a strong cytotoxic potential (IC_50_: 20–30 nM) in Ramos Burkitt lymphoma cells that was in the range of dinaciclib (IC_50_: 10 nM). Using a kinome screen, almost all CDKs could be identified as targets of meriolin 16 and 36. In addition, we modeled the interactions of meriolin 16 and 36 with the ATP-binding pocket of the targeted CDKs, revealing two possible binding modes for meriolin 36. Accordingly, cell cycle progression and proliferation were blocked due to meriolin-induced CDK inhibition that was further corroborated by the loss of the phosphorylation of the CDK2 target retinoblastoma protein at Ser612 and Thr821. Beside cell cycle inhibition, meriolin 16 also prevented transcription initiation due to the inhibition of CDK9-mediated phosphorylation of RNA polymerase II at Ser2. Thus, meriolins serve as a versatile tool for cancer therapy since they target tumor growth via inhibition of cell cycle, proliferation and gene transcription, and activate the endogenous suicide program.

## Results

### Meriolin 16 and 36 are highly cytotoxic in different human leukemia and lymphoma cell lines and in malignant primary cells derived from leukemia and lymphoma patients

**Fig. 1:**
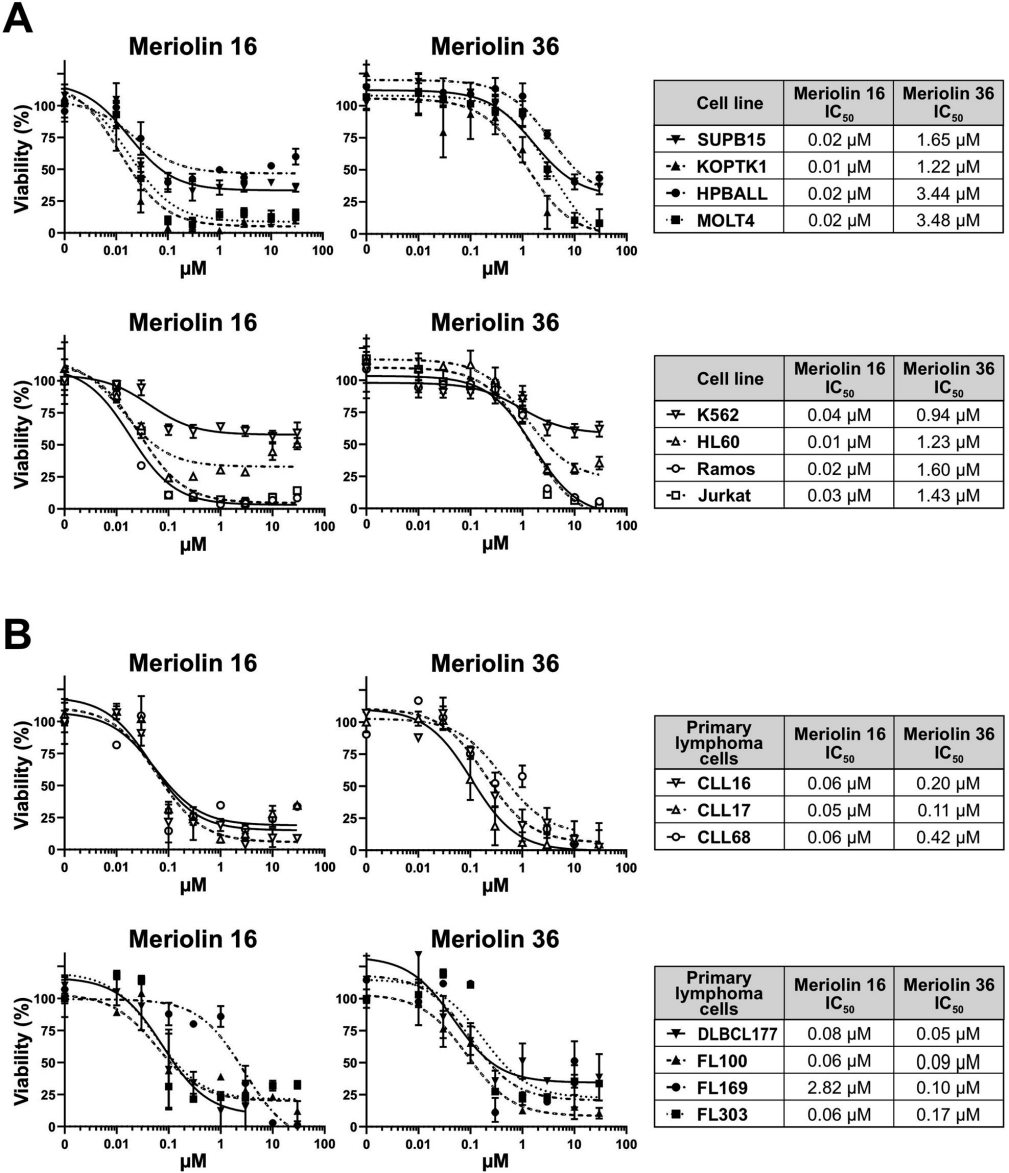
Meriolin 16 and meriolin 36 are highly cytotoxic to myeloid and lymphoid cell lines and also to primary malignant patient cells. **A** Cytotoxicity was determined after 24 h with increasing concentrations of meriolin 16 and meriolin 36 in two myeloid cell lines (K562 (chronic myeloid leukemia; CML) and HL60 (acute myeloid leukemia; AML)) and six lymphoid cell lines (SUPB15 (B cell acute lymphoblastic leukemia; B-ALL), KOPTK1 (T-ALL), HPBALL (T cell acute lymphoblastic leukemia; T-ALL), MOLT4 (T-ALL), Ramos (Burkitt B cell lymphoma) and Jurkat (acute T cell lymphoblastic leukemia; T-ALL)). Cell viability was assessed by AlamarBlue® viability assay. Error bars = Mean ± SD of a representative experiment performed in triplicates. **B** Cytotoxicity was determined after 24 h with increasing concentrations of meriolin 16 and meriolin 36 in malignant primary patient cells such as diffuse large B cell lymphoma cells (DLBCL), follicular lymphoma cells (FL) and chronic lymphocytic leukemia cells (CLL) by AlamarBlue® assay. Error bars = Mean ± SD of a representative experiment performed in triplicates. The respective IC_50_ values are given on the right side for the respective compound in each cell line. n.d. = not detected in the depicted concentration range.

Based on the apoptotic capacity of meriolin 31 [22], a novel derivative (termed meriolin 16) with an additional methoxy group was synthesized with the intention of enhancing its cytotoxicity (Supplementary Fig. 2B). We analyzed the cytotoxic potential of the novel derivative meriolin 16 compared to the previously described derivative meriolin 36 [22] in different human leukemia and lymphoma cell lines (i.e., HL60 (acute myeloid leukemia; AML), Jurkat (T cell acute lymphoblastic leukemia; T-ALL), HPBALL (T-ALL), K562 (chronic myeloid leukemia; CML), KOPTK1 (T-ALL), MOLT4 (T-ALL), SUPB15 (B cell acute lymphoblastic leukemia; B-ALL), and Ramos (B cell Burkitt lymphoma)). As shown in Fig. 1A, meriolin 16 potently induced cell death at nanomolar range (IC_50_ values ranging from 10 to 40 nM) in all cell lines tested and was substantially more cytotoxic than meriolin 36 (IC_50_ values ranging from 940 to 3840 nM). In addition, both meriolin derivatives were highly cytotoxic in malignant primary cells derived from patients with diffuse large B cell lymphoma cells (DLBCL), follicular lymphoma cells (FL) or chronic lymphocytic leukemia cells (CLL) (Fig. 1B).

### In vitro kinase assays and kinome screen reveal meriolin 16 and 36 as potent inhibitors of cyclin-dependent kinases (CDKs)

**Fig. 2:**
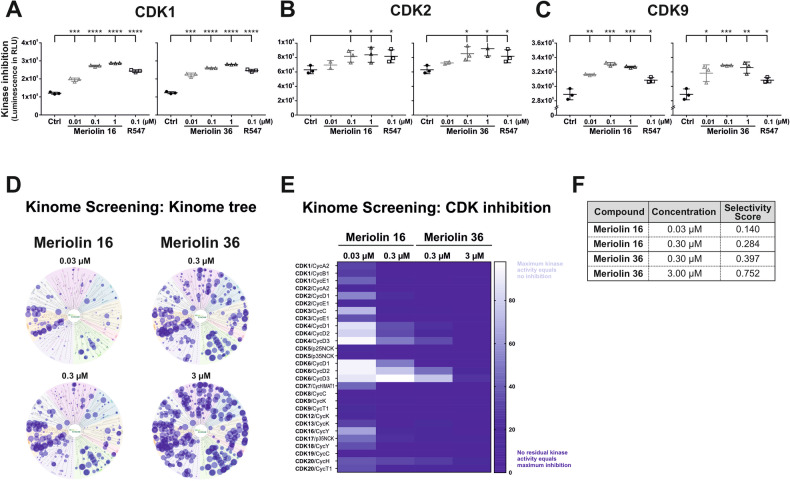
Meriolin 16 and 36 inhibit CDK1, 2, and 9 and inhibit a variety of other kinases in a kinome screen with a prevalence to the CMGC family. A luminescence-based kinase activity assay was used to determine the inhibitory activity of meriolin 16 and 36 on selected CDKs and their corresponding cyclins with the usage of CDK Kits from BPS Bioscience (**A**) CDK1/cyclin B1; (**B**) CDK2/cyclin A2; (**C**) CDK9/cyclin T. In this kinase assays, the relative luminescence correlates with the inhibitory capacity of the treatment and concentration, respectively. DMSO (0.1% v/v) was used as solvent control and R547 as positive control (Selleckchem.com: inhibitor of CDK1/2 and 4). Error bars = mean ± SD values of three independent biological experiments are shown. Statistical analysis: one-way ANOVA, Bonferroni’s multiple comparison test; (****p ≤ 0.0001). **D** Kinome screening with meriolin 16 and 36. Kinase inhibition by meriolin 16 (0.03 µM and 0.3 µM) and meriolin 36 (0.3 µM and 3 µM) is depicted as kinome tree. The diameter of dots reflects % inhibition of 335 kinases (atypical kinases DNAPK, EEF2K mTOR and PKMzeta were excluded), the scale is 0 to ≥100% inhibition. **E** Heatmap of the CDK/cyclin family with the residual kinase activity upon inhibition with meriolin 16 (0.03 µM and 0.3 µM) and meriolin 36 (0.3 µM and 3 µM). This heat map shows kinase inhibition by meriolins from dark blue (maximum inhibition of the kinase, 0%) to white (maximum kinase activity, 100%). **F** Shown in this table are the selectivity scores which were calculated for both concentrations of meriolin 16 (0.03 µM and 0.30 µM) and meriolin 36 (0.30 µM and 3.00 µM).

Meriolin derivatives have been shown to inhibit a variety of CDKs [16, 17, 21]. Therefore, we used a luminescence-based kinase activity assay to investigate in how far meriolin 16 and 36 might inhibit CDK1, CDK2 and CDK9. These three CDKs were selected due to their cell cycle regulatory function (CDK1 and 2) or transcriptional control (CDK9). The CDK 1, 2 and 4 specific inhibitor R547 was used as positive control [29]. Meriolin 16 and meriolin 36 inhibited CDK1/cyclin B1, CDK2/cyclin A2 and CDK9/cyclin T in a concentration-dependent manner and in a similar range as R547—though the inhibitory effect on CDK2 was less pronounced (Fig. 2A–C).

In order to accomplish a comprehensive approach, we performed a kinome screening. In this screening, the inhibitory activity of meriolin 16 and 36 was analyzed on a panel of 335 kinases (performed by ^33^PanQinase^TM^ from Reaction Biology). Meriolin 16 and meriolin 36 were tested in two concentrations according to their differing IC_50_ values evaluated in preliminary experiments in Ramos cells (i.e., meriolin 16 with 0.03 µM and 0.3 µM, meriolin 36 with 0.3 µM and 3 µM). The results are shown as a kinome tree in Fig. 2D (a comprehensive inhibition profile of all 335 kinases tested is provided in Supplementary Table 1). We observed that the lower concentration of meriolin 16 (0.03 µM; which equals the IC_50_ value in Ramos cells) showed a high specificity for kinases within the yellow area of the kinome tree, which represents the CMGC family. The CMGC family consists of CDKs, MAPKs (mitogen-activated protein kinases), GSKs (glycogen synthase kinases) and CLKs (CDC-like kinases) [19, 22]. When the concentration was increased to 0.3 µM, a more unspecific inhibition pattern for meriolin 16 was observed, with increasing diameter of the dots of the CMGC family correlating with increased inhibitory activity. For meriolin 36, the concentration of 0.3 µM shows a comparable pattern of dots to the kinome tree with 0.3 µM of meriolin 16. Thus, it appears that the two meriolin derivatives are more similar in their CDK inhibitory activity than in their cytotoxic potential. Again, for meriolin 36, a prevalence for the CMGC family but also a specificity for the AGC family (cAMP-dependent protein kinase (PKA), the cGMP-dependent protein kinase (PKG), and the protein kinase C (PKC); green area), consisting of 63 evolutionarily related serine/threonine protein kinases [30], could be observed. At 3 µM, meriolin 36 inhibited almost all kinases tested, at least to a lower extent. In Fig. 2E, a heatmap of the inhibition profile of meriolin 16 and 36 on all CDKs tested within the kinome screen is provided. Thus, in addition to CDK1, 2 and 9 (Fig. 2A–C) almost all tested CDKs (in complex with their respective cyclins) were inhibited (such as CDK1, 2, 3, 5, 7, 8, 9, 12, 13, 16, 17, 18, 19, 20) as shown in the heatmap in Fig. 2E. However, CDK4 and 6 were inhibited to a lesser extent—especially at the lower concentration (0.03 µM) of meriolin 16. Intriguingly, CDK9, which regulates transcription via phosphorylation of RNA polymerase II, was also completely inhibited by both meriolin derivatives.

In order to determine the overall specificity, a selectivity score was calculated, where a high specificity is characterized by a low value. As shown in Fig. 2F, meriolin 16 displayed the highest selectivity at 0.03 µM with a score of 0.140, while meriolin 36 was less specific.

### Drug-to-target modeling studies of the binding mode of meriolin 16 and 36 to the ATP-pocket of CDK2

The kinome-wide assay results showed that both meriolin derivatives are active against CDKs. To further investigate the structural basis of the binding mechanism, we performed molecular docking studies on CDKs. Since the active site across all the inhibited CDKs is fairly conserved (Supplementary Fig. 3), meriolins should share a similar binding mode for each protein. To assess this, we docked both meriolin 16 and 36 into the structures of the active forms of CDK1, 2, and 9. Our results show an overall similar binding mode across all different CDKs, with the indole moiety positioned next to the loop between the β5 strand and the α2 helix for both compounds. In meriolin 36, small variations in the position of the benzene group can be observed (Supplementary Fig. 4). Since the binding mode is conserved among all CDKs, we chose CDK2 as a representative system to investigate the binding pose of both meriolins in more detail.

**Fig. 3:**
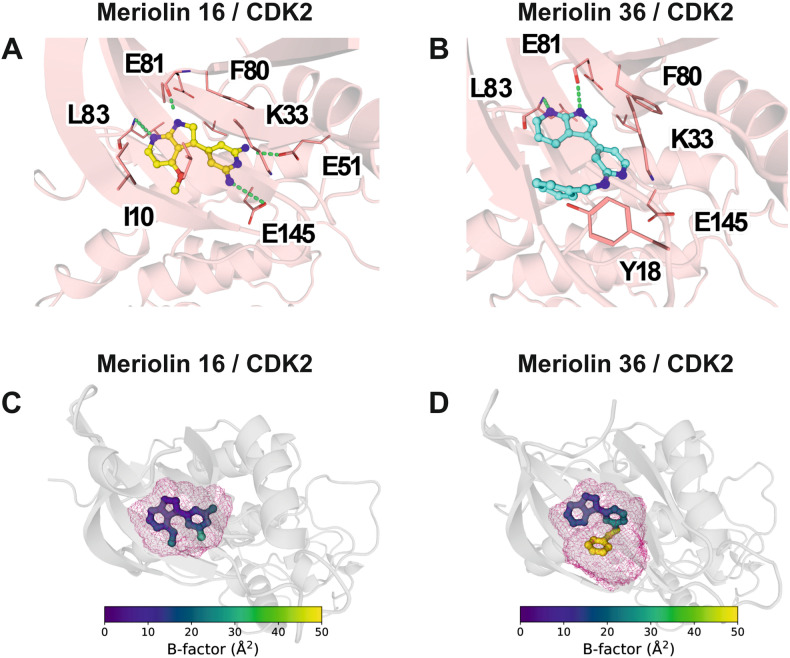
Binding mode of meriolin 16 and meriolin 36 in CDK2. Binding mode of meriolin 16 (**A**) and meriolin 36 (**B**) in the active site of CDK2 predicted by docking and molecular dynamics simulations. Residues forming direct interactions with the ligands are labeled. Green dashed lines indicate polar interactions with the ligand. Mobility of the ligands throughout the course of the molecular dynamics simulations expressed as volumetric occupancy (pink mesh) and per-atom B-factor (color code according to the given scale; higher values indicate higher mobility) for meriolin 16 (**C**) and meriolin 36 (**D**).

In both derivatives, the indole moiety forms non-covalent interactions with the backbone of the conserved residues Leu83 and Glu81 (Fig. 3A, B). In case of meriolin 16, we observed electrostatic interactions between the amino groups of the di-aminopyridine moiety and Glu145 and Glu51. Glu51 is part of the C-helix, a critical secondary structure element that undergoes major conformational rearrangements upon CDK activation [31]. The observed binding mode is in agreement with what has been experimentally observed for other indole-containing molecules through X-ray crystallography [16], where the indole binds to the same backbone atoms of the protein. Nonetheless, our initial docking results also showed an alternative possible binding mode with a better docking score for meriolin 36 (−10.02 vs. −10.81 kcal mol^−1^). We termed this alternative pose “flipped mode”, as in this case the aminopyridyl region is making interactions with the backbone atoms of Leu83, and the indole moiety of meriolin 36 is placed deeper in the protein pocket surrounded by hydrophobic residues such as Val18, Phe80 and Leu134 (Supplementary Fig. 5A).

To further corroborate our binding mode predictions, we performed microsecond-long all-atom molecular dynamics simulations and assessed the overall stability of the docked poses. To quantify how tightly each ligand fits into the active site of CDK2, we calculated the B-factor of each atom of meriolin 16 and 36, which measures how mobile the atoms are through the course of the simulations, and we also calculated the total volume that each ligand occupied within the protein through the simulations (Fig. 3C, D). The results show that meriolin 16 remained tightly bound within the active site throughout the simulations as evidenced by the low B-factor values and the small effective volume occupied (Fig. 3C). Meriolin 36 also remains bound through the entire 5 µs sampled, however, different behaviors can be observed at different regions of the molecule. The indole moiety remains firmly placed in the loop between β5 strand and α2 helix, whereas the benzene group moves around the pocket due to the lack of prominent hydrophobic interactions, as can be seen by the high B-factor values of this moiety, and the shape of the volume used by the ligand (Fig. 3D).

Simulations of the flipped mode of meriolin 36 show an overall more mobile pose compared to the canonical binding pose, with both the benzene and the indole group displaying higher B-factor values than in the standard mode and showing a bigger effective volume due to the constant tumbling of the ligand within the active site, suggesting weaker interactions with the protein site (Supplementary Fig. 5B). The exposed hydrophobic surface on ligands is commonly associated with higher desolvation penalties and a worse fit to the active site [32]. For this reason, we quantified the solvent-accessible surface area (SASA) of meriolin 36 on its standard and flipped binding mode. The results show a significant increase of SASA when meriolin 36 is bound in the putative flipped mode (Supplementary Fig. 5C).

CDKs undergo drastic conformational rearrangements upon activation by cyclins, which are essential for the protein function. Interestingly, crystal structures show that ATP can bind to CDK2 in its inactive state [33]. Since kinase inhibitors can be specific for active or inactive states [34], we tested whether meriolin 16 and 36 could also bind to CDKs in their inactive conformational state by docking both compounds onto the inactive ATP-bound crystal structure of CDK2. Only meriolin 16 yielded a binding pose with a score below −5.0 kcal mol^−1^. In this pose, the di-aminopyridyl moiety occupies the ribose binding region of the active site (Supplementary Fig. 5D, left panel). To assess the robustness of this alternative complex, we also performed molecular dynamics simulations starting from this docking pose. In four out of five replicas, we observed dissociation of the ligand within the first 500 ns of simulation (Supplementary Fig. 5D, right panel), which is indicative of a false-positive binding mode [35]. Therefore, the conformational changes triggered by the presence of a cyclin partner seem to be essential for the meriolin binding. Thus, in contrast to meriolin 36, which only binds to the active conformational state, meriolin 16 can also bind to the inactive state of CDK2.

### Effect of meriolin 16 and 36 on CDK-mediated phosphorylation of the retinoblastoma (RB) protein

Since both meriolin derivatives selectively inhibited CDKs at low concentrations (though also other kinases at higher concentrations; see Fig. 2D and Supplementary Table 1), we focused on the downstream signaling of cell cycle regulation in G_1_, S, G_2_ and M phase. In G_1_ phase, accumulation of CDK4/6/cyclin D is necessary for cell cycle entry [5]. CDK4/6/cyclin D are able to monophosphorylate the retinoblastoma (RB) protein at any of its 14 known phosphosites and CDK1 and 2 are the main contributors to hyperphosphorylation of RB [6, 36]. The RB protein is the major regulator protein for gene expression, since in its hypo- or mono-phosphorylated form (in complex with the transcription factor DP) it represses E2F-dependent gene expression during G_1_. This complex dissociates from E2F-regulated genes when RB is phosphorylated by CDK4/6/cyclin D at Ser249 and Thr252 (for overview see Supplementary Fig. 1).

To investigate the possible effect of CDK inhibition by meriolins on the RB protein, Ramos cells were treated with meriolin 16 and 36 at 0.1 µM or 1 µM in a kinetics up to 24 h. Subsequently, the expression of cyclin B1, cyclin D3, phospho-RB (p-Ser249 and p-Thr252) and CDK1 was monitored by immunoblotting and quantified respectively. Thus, we observed that meriolin 16 reduced the phosphorylation of RB at p-Ser249 and p-Thr252 after 24 h, whereas meriolin 36 had no effect on these RB-phosphosites. The CDK1, 2 and 4 specific inhibitor R547 [29] however, completely abrogated the phosphorylation of RB within 4 h (Supplementary Fig. 6A, B). Since meriolin 16 and 36, in contrast to R547, do not inhibit CDK4 as strongly at the applied concentrations, this might explain why the effect on the CDK4-mediated phosphorylation of RB at p-Ser249/p-Thr252 was less pronounced. The cyclin D3 levels were not impaired by meriolins or R547 (Supplementary Fig. 6A, B). In contrast to meriolin 36 and R547, which rather increased the expression of cyclin B1 after 24 h, meriolin 16 reduced the expression of cyclin B1, whereas the expression of CDK1 was not affected by meriolins or R547 (Supplementary Fig. 6C, D).

**Fig. 4:**
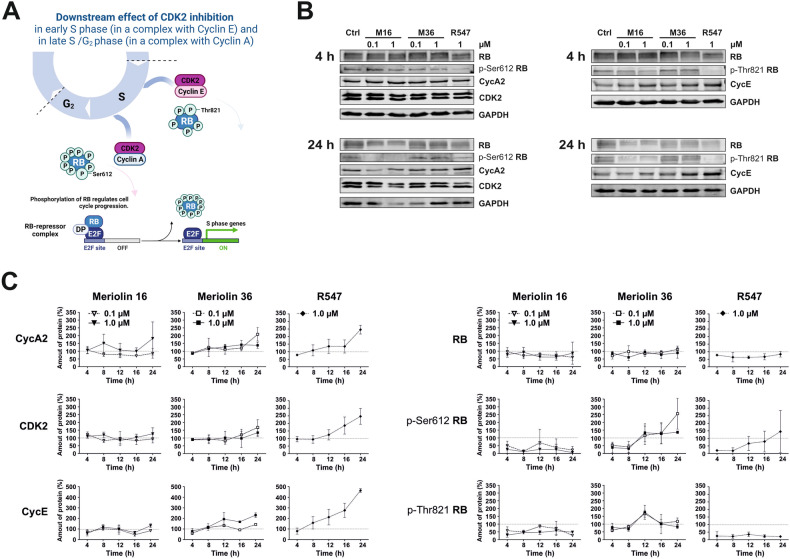
CDK2/cyclin A- and CDK2/cyclin E-inhibition by meriolin 16 and meriolin 36 impair downstream phosphorylation of the main cell cycle regulating protein retinoblastoma (RB). **A** Graphical abstract visualizing the part of the cell cycle which is analyzed in this figure. The downstream effect of CDK2 inhibition in a complex with cyclin E representing the early S phase and in a complex with cyclin A representing late S phase early G_2_ phase. The respective phosphosites of the RB protein are shown, Thr821 is phosphorylated in early S phase by CDK2/cyclin E and Ser612 is phosphorylated in late S phase early G_2_ phase by CDK2/cyclin A. These phosphorylations ensure propagation of the cell cycle, allowing transcription of E2F-dependent S phase genes due to the phosphorylation-mediated inhibition of RB. **B** Ramos cells were treated for 4, 8, 12, 16 and 24 h with meriolin 16 (0.1 and 1 µM) and meriolin 36 (0.1 and 1 µM) and with R547 (1 µM as comparative CDK inhibitor), untreated cells are shown as negative control. Two representative immunoblots of each phase (left two blots for early S phase; right two blots for late S/early G_2_ phase) from ≥2 independent biological replicates for each time point are shown for the detection of cyclin A2, CDK2, RB and p-Ser612 RB and also for cyclin E and RB with p-Thr821 RB. (GAPDH served as loading control). The immunoblots after 8, 12 and 16 h are shown in Supplementary Fig. 7A, B. **C** Shown is the quantification of a time kinetics of the immunoblots for cyclin A2, CDKs, cyclin E, RB and the two phosphosites p-Ser612 RB and p-Thr821 RB. Error bars = Mean ± SD values of ≥2 independent biological experiments are shown. Dashed lines indicate 100% amount of protein of control. The immunoblots after 8, 12 and 16 h are shown in Supplementary Fig. 7A, B.

Next, we investigated the effect of meriolin 16 and 36 on the CDK2-mediated phosphorylation of RB at Ser612 and Thr821. Sequential phosphorylation of the suppressor protein RB by CDKs ensures the inactivation of the suppressor activity of RB and thereby allows cell cycle progression. CDK2 is associated with cyclin E in early S phase and with cyclin A in late S/G2 phase and plays a crucial role in genome replication [5]. At the end of the S phase, cyclin A replaces cyclin E by forming a new complex with CDK2, then cyclin E is degraded [37]. The CDK2/cyclin A complex is responsible for the termination of the S phase, driving the transition from S phase to G_2_, with the subsequent activation of CDK1 by cyclin A allowing the cell to enter M phase [37] (a schematic overview is given in Fig. 4A). To investigate the influence on the signaling in the S phase of the cell cycle, the protein levels of CDK2, cyclin A2, cyclin E, and phosphorylation of RB at Ser612 and Thr821 were analyzed via immunoblotting (Fig. 4B, C and Supplementary Fig. 7A, B). Quantification of the immunoblot kinetics revealed that CDK2 and RB protein levels remained stable upon meriolin 16 and meriolin 36 treatment. Greater variations were observed for cyclin A2 and cyclin E protein levels upon treatment with meriolin 16 and 36 (Fig. 4C). The phosphosites p-Thr821 (which gets phosphorylated in early S phase by CDK2/cyclin E) and p-Ser612 of RB (which is phosphorylated in late S phase by CDK2/cyclin A2) were no longer phosphorylated upon meriolin 16 treatment. This effect was not observed with meriolin 36 treatment. The application of the CDK1, 2 and 4 inhibitor R547 [29] resulted in increased protein levels of CDK2, cyclin A2 and cyclin E and the loss of phosphorylation of RB at p-Thr821 (Fig. 4C). Inhibition of CDK2 by meriolins (either in complex with cyclin A2 or with cyclin E) did not result in a decrease of the respective protein level. However, the downstream target suppressor protein RB was affected by R547.

In general, the phosphorylation at p-Ser612-RB enhances cell cycle progression in S phase [38] and the phosphorylation of RB results in the release of E2F family members and enables the transcription of crucial E2F-responsive genes for the S phase [39]. Since meriolin 16 substantially reduced the phosphorylation at p-Ser612 and p-Thr821 of RB (Fig. 4C), it most likely would affect cell cycle progression. The results shown in Fig. 4 were further supported by immunopurification of the RB protein after meriolin 16 treatment (0.1 and 1 µM) for 4 and 24 h (Supplementary Fig. 8). Thus, the phosphosites p-Ser612 and p-Thr821 of RB were less phosphorylated after 4 h upon 1 µM meriolin 16 treatment and completely abrogated after 24 h (Supplementary Fig. 8).

### Meriolins and other known CDK inhibitors induce cell cycle arrest and reduce proliferation at sublethal doses

**Fig. 5:**
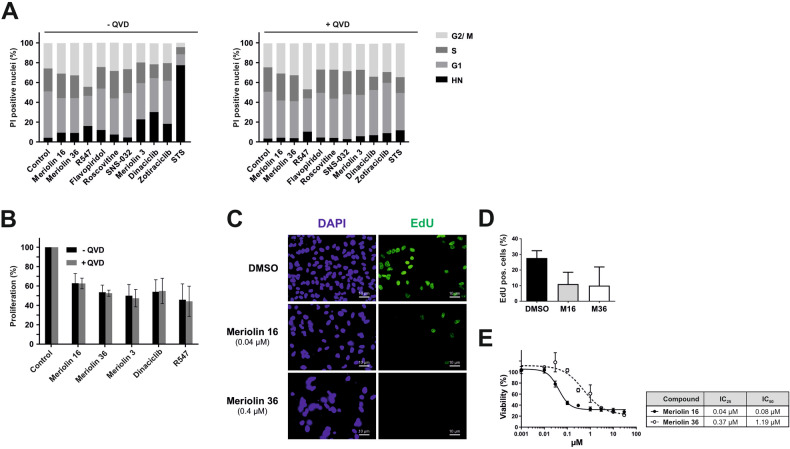
Meriolins and other known CDK inhibitors induce cell cycle arrest and reduce proliferation in sublethal doses. **A** The detection of cell cycle phases was performed by flow-cytometric analysis of the DNA content of propidium iodide-stained nuclei using the Nicoletti assay [41]. Since the DNA content doubles during S phase, a higher fluorescence intensity can be detected in G_2_ compared to G_1_ phase. Due to the caspase dependent DNA fragmentation and subsequent leakage of fragmented DNA from apoptotic nuclei, apoptosis can be determined by the formation of hypodiploid nuclei (HN). Cell cycle analysis in Ramos cells was determined after 24 h treatment without (left diagram) and with (right diagram) pre-and co-treatment of the pan-caspase inhibitor QVD (10 µM), followed by a sublethal dosage of each of the respective CDK inhibitor (meriolin 16 (0.02 µM), meriolin 36 (2 µM), R547 (1 µM), flavopiridol (0.1 µM), roscovitine (10 µM), SNS-032 (0.15 µM), meriolin 3 (0.1 µM), dinaciclib (0.01 µM), zotiraciclib (0.1 µM)). The broad kinase inhibitor and potent apoptotic stimulus staurosporine (STS; 2.5 µM) was used as positive control for apoptosis induction and DMSO (0.1% v/v) as diluent control. Untreated cells are shown as control. Error bars = Mean ± SD of three independent experiments performed in triplicates. **B** Proliferation was measured by the incorporation of BrdU with the BrdU cell proliferation assay. 1 × 10^4^ Ramos cells were pre-incubated with the compounds for 24 h: meriolin 16 (0.02 µM), meriolin 36 (0.1 µM), meriolin 3 (0.1 µM), dinaciclib (0.01 µM), R547 (1 µM), or medium (Control). One hour post treatment with the compounds of interest, the BrdU solution was added and further incubated with the compounds in order to monitor residual proliferative capacity. Error bars = Mean ± SD of ≥4 independent experiments performed in triplicates. **C** Measurement of proliferation-inhibition by microscopic analysis of EdU-incorporation in HeLa cells. In the EdU assay, the incorporation of EdU (thymidine nucleoside analog) into the DNA was measured and serves as a parameter of proliferation. HeLa cells were treated with the respective IC_25_ values (as evaluated in **E**) of meriolin 16 (0.04 µM), meriolin 36 (0.4 µM) or DMSO (0.1% v/v) as solvent control. The EdU-incorporation was analyzed by immunofluorescence and exemplary microscopy images are shown (green: EdU-incorporation; blue: DAPI stained nuclei). **D** The quantification of EdU-positive cells from several microscopy images is shown as bar graph of relative EdU positive cells in %. (Biological replicates *n* = 1–2; 10 cells were counted for DMSO, 30 for meriolin 16, 22 for meriolin 36). **E** Evaluation of IC_25_ and IC_50_ values of HeLa cells. HeLa cells were treated for 24 h with concentrations between 0.01 μM and 30 μM of meriolin 16 or meriolin 36. Cell viability was measured by AlamarBlue assay; *n* = 1.

Next, the effect of meriolins and other CDK inhibitors on the cell cycle was investigated. For this, we used established CDK inhibitors (such as dinaciclib, flavopiridol, meriolin 3, R547, roscovitine, SNS-032, and zotiraciclib), some of which are presently undergoing clinical trials. Meriolin 3 was included since it is the most potent meriolin derivative described to date [16, 17, 25] and has already been tested in preclinical trials [12, 21, 40]. Since CDK inhibitors can activate caspases and thereby generate hypodiploid apoptotic nuclei that may interfere with cell cycle analysis, Ramos cells were pretreated with the pan-caspase inhibitor Q-VD-OPh (QVD). This procedure should enable the differentiation between caspase-dependent (apoptotic) and non-caspase-dependent effects on the DNA content and cell cycle. Subsequently, cells were incubated with a non-lethal dose of the respective CDK inhibitors. The broad kinase inhibitor and potent apoptotic stimulus staurosporine (STS) was used as positive control. The detection of the different cell cycle phases was performed by flow-cytometric analysis of the DNA content of propidium iodide-stained nuclei [41]. Though non-lethal dosages were applied, the different compounds induced residual apoptosis as indicated by the formation of hypodiploid apoptotic nuclei, which was completely abrogated upon addition of the pan-caspase inhibitor QVD (Fig. 5A). However, even in the presence of QVD, meriolin 16, meriolin 36, and most of the CDK inhibitors displayed only a slight shift to G_2_ phase. Only R547 induced a pronounced G_2_ arrest. This observation was further analyzed by measuring the proliferative activity using the BrdU assay. As shown in Fig. 5B, treatment of Ramos cells with non-lethal doses of meriolin 16, meriolin 36, meriolin 3, dinaciclib and R547 induced a substantial decrease in proliferation after 24 h—which was not affected by caspase inhibition via QVD. In addition to the BrdU assay, EdU incorporation was analyzed by microscopy in HeLa cells. For this, HeLa cells were treated for 24 h with non-lethal doses of meriolin 16 and 36 and residual EdU incorporation was detected via immunofluorescence and quantified as shown in Fig. 5C–E. After 24 h treatment, the EdU incorporation and thus proliferation were reduced to 10%. Therefore, meriolin 16 and 36 induce an arrest in DNA replication and proliferation at non-lethal doses.

### Meriolin 16 and 36 impair CDK9-mediated downstream phosphorylation of the transcriptional regulator RNA polymerase II

**Fig. 6:**
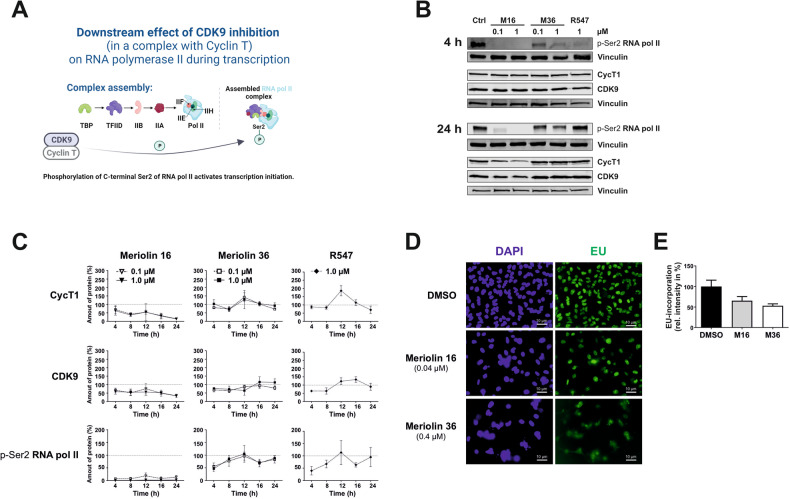
CDK9/cyclin T-inhibition by meriolin 16 and meriolin 36 impair downstream phosphorylation of the key transcription regulating protein RNA polymerase II (RNA pol II). **A** Graphical abstract visualizing the role of CDK9 in a complex with cyclin T during transcription. The phosphosite Ser2 in the C-terminal domain of RNA pol II is phosphorylated during transcription initiation and elongation. CDK9/cyclin T phosphorylates Ser2 as part of the positive transcription elongation factor (P-TEFb) complex and mediates the transition from transcription initiation to productive elongation of pre-mRNA transcripts. **B** Ramos cells were treated for 4, 8, 12, 16 and 24 h (untreated cells are shown as control), meriolin 16 (0.1 and 1 µM; M16) and meriolin 36 (0.1 and 1 µM; M36) and with R547 (1 µM as comparative CDK inhibitor). Two representative immunoblots of three independent biological replicates for each time point are shown for the detection of cyclin T1, CDK9 and p-Ser2 of RNA pol II. (Vinculin served as loading control). The immunoblots after 8, 12 and 16 h are shown in Supplementary Fig. 7C. **C** Quantification of a time kinetics of the immunoblots for cyclin T1, CDK9 and p-Ser2 of RNA pol II. Error bars = Mean ± SD values of three independent biological experiments are shown. Dashed lines indicate 100% amount of protein of control. The immunoblots after 8, 12 and 16 h are provided in Supplementary Fig. 7C. **D** HeLa cells were treated with meriolin 16 (0.04 µM) and 36 (0.4 µM) and DMSO for 24 h. By microscopy, EU-incorporation (green) was determined and shown are exemplary microscopy images (DAPI in blue stains the nuclei). **E** From different microscopy images (biological replicates *n* = 2; 30 cells were counted for DMSO, 30 for meriolin 16, 30 for meriolin 36) DMSO-treated cells and their EU-incorporation was set to 100%, shown is the EU-positive relative intensity in %.

Since meriolin 16 and 36 also potently inhibited CDK9 (Fig. 2E), we investigated in how far the downstream signaling of CDK9 was affected by both meriolin derivatives. CDK9 is not primarily involved in cell cycle regulation, but in transcriptional control by activating RNA polymerase II (a schematic overview is provided in Fig. 6A). Therefore, we examined whether meriolin 16 and 36 inhibited the CDK9/cyclin T mediated phosphorylation of RNA polymerase II at the transcriptional crucial phosphosite p-Ser2. For this, we analyzed the protein levels of CDK9/cyclin T1 and the phosphorylation of RNA polymerase II (at p-Ser2) upon treatment with meriolin 16, 36 or R547 over time (4, 8, 12, 16 and 24 h) via immunoblotting. Meriolin 16 induced a decrease of CDK9 and cyclin T1 protein levels over 4, 8, 12, 16 and 24 h at low (0.1 µM) and high (1 µM) concentrations. In contrast, these protein levels were relatively stable for meriolin 36 (Fig. 6B, C and Supplementary Fig. 7C). Both meriolin 16 concentrations resulted in a total loss of the phosphorylation at Ser2 within 4 h. In contrast, meriolin 36 and R547 mediated reduction of the phosphorylation at Ser2 was less pronounced (Fig. 6B, C and Supplementary Fig. 7C). Comparing both meriolins, meriolin 16 treatment had a higher impact on this phosphorylation than meriolin 36.

It has been shown that the CDK9/cyclin T1 induced phosphorylation at Ser2 within the C-terminal domain (CTD) of RNA polymerase II mediates the transition from transcription initiation to elongation [42–44]. Since meriolin 16 completely abrogated the transcription-initiating phosphorylation at Ser2 of RNA polymerase II (Fig. 6B, C and Supplementary Fig. 7C), it would consequently inhibit the transcriptional activity. Therefore, we measured the de novo RNA-synthesis by the incorporation of 5-ethynyl-uridin (EU) in HeLa cells treated with the respective non-lethal IC_25_ concentrations of meriolin 16 (0.04 µM) and meriolin 36 (0.4 µM) for 24 h. As shown in the immunofluorescence analysis in Fig. 6D, E, EU incorporation was reduced to ~60% by meriolin 16 and to ~50% by meriolin 36. Thus, both meriolin derivatives impair de novo RNA-synthesis and transcription at non-lethal concentrations.

### Meriolins are highly cytotoxic compared to other CDK inhibitors

**Fig. 7:**
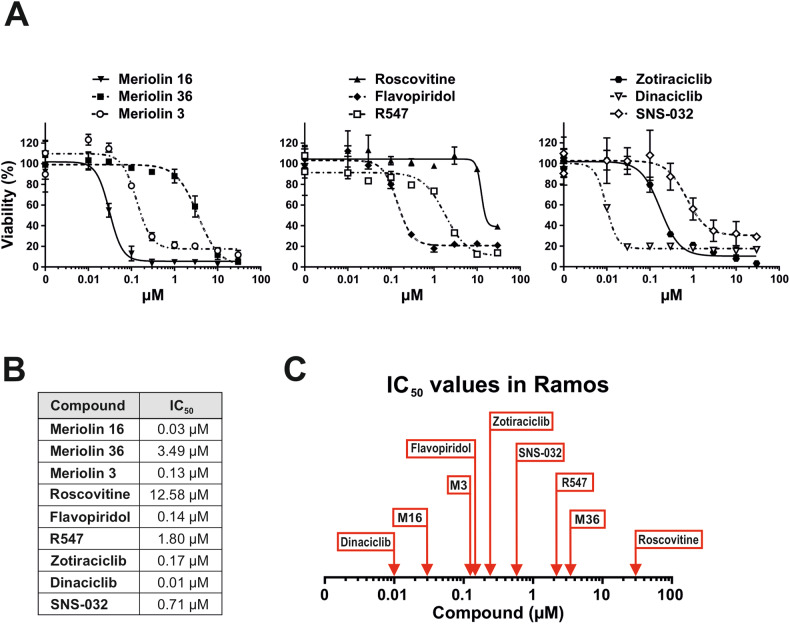
Meriolins are highly cytotoxic compared to known CDK inhibitors. **A** Cytotoxicity was determined after 24 h with increasing concentrations of meriolin 16 (M16), meriolin 36 (M36), meriolin 3 (M3) (left graph), roscovitine, flavopiridol, R547 (middle graph), zotiraciclib, dinaciclib, SNS-032 (right graph) in the Burkitt B cell lymphoma cell line Ramos. Cell viability was assessed by AlamarBlue® assay. Error bars = Mean ± SD of a representative experiment performed in triplicates. **B** The respective IC_50_ values are shown in the table. n.d. = not detected in the depicted concentration range. **C** Comparative overview of the determined IC_50_ values in µM after 24 h incubation with the respective compounds in Ramos cells.

Finally, we compared the cytotoxic potential of meriolin 16 and 36 with known CDK inhibitors like roscovitine, flavopiridol, R547, meriolin 3, zotiraciclib, dinaciclib and SNS-032. These CDK inhibitors were selected based on their clinical development or therapeutic use and CDK inhibition profile, which are summarized in Supplementary Table 2. Meriolin 3 has been synthesized by others and was included since it represents the most potent meriolin derivative described so far [16, 17, 25]. For this, Ramos lymphoma cells were treated with meriolins or other CDK inhibitors at increasing concentrations for 24 h and the cell viability was determined by AlamarBlue® assay. As shown in Fig. 7, meriolin 16 was the most active derivative amongst all meriolins tested—even more potent than meriolin 3. With an IC_50_ value of 30 nM, meriolin 16 was even in the range of dinaciclib (IC_50_ value: 10 nM), and both compounds were by far more cytotoxic than any other CDK inhibitor tested (Fig. 7B, C).

Finally, we assessed the applicability of our meriolin derivatives as drug candidates and performed ADME (absorption, distribution, metabolism and excretion) predictions using the Swiss-ADME tool [45]. The results are summarized in Supplementary Table 3. Thus, both meriolin 16 and 36 exhibit a combination of lipophilicity and solubility that should allow gastrointestinal absorption and provide a drug-like scaffold suitable for further lead optimization processes.

## Discussion

Dysregulation of cell cycle control is a major hallmark of cancer, achieved through the selective and aberrant activation of CDKs. Tumor cells with disrupted cell cycle control are particularly prone to undergo apoptosis, rendering them more susceptible to targeted therapy compared to healthy cells. Inhibiting CDKs has emerged as an appealing strategy in cancer therapy. While conventional approaches have focused on disrupting the integrity or replication of cancer cells’ DNA by applying alkylating agents, anti-metabolites, topoisomerase inhibitors, or inhibitors that affect mitotic spindle assembly/disassembly, novel targeted strategies have concentrated on developing inhibitors for crucial kinases for respective cancer entities [40, 46]. CDK1/cyclin B plays an essential role in mitotic functions and possesses a unique ability to compensate for other CDKs and is overexpressed in B cell lymphoma [47]. Consequently, targeting of CDK1 presents an appealing approach to inhibit cell proliferation. Various strategies aiming to target CDK1 and cyclin B have been proposed and demonstrated to effectively inhibit tumor growth [48–54]. Similarly, intervening with the functions of CDK2 during DNA replication and S phase progression offers a promising possibility for cancer therapeutics [40]. According to the dataset of proteinatlas.org, CDK2 expression is increased in 50% of lymphoma. Additionally, CDK4/cyclin D kinase has emerged as a well-established pharmacological target in several human cancers, particularly in melanoma and KRAS-mutant non-small cell lung cancers (NSCLC) [55, 56].

Like their natural counterparts (meridianins and variolins), meriolins display a pronounced inhibitory potential on a broad range of CDKs and appear to be even more potent—both in vitro and in vivo—compared to variolins and meridianins [17, 18, 22, 25, 57–59]. Thus, it has been demonstrated that meriolins exhibit potent cytotoxicity in the nanomolar range across various tumor cell lines of diverse origins, including colon cancer (HCT116, LS-174T), hepatoma (Huh7, F1), cervical cancer (HeLa), breast cancer (MCF-7), glioma (GBM, SW1088, U87), neuroblastoma (SH-SY5Y), leukemia (Jurkat, Molt-4), lymphoma (Ramos), and myeloma (KMS-11) [16, 21, 22, 24, 25, 60]. In addition, we previously showed that meriolin 16 and 36 were able to induce cell death in imatinib-resistant K562 chronic myeloid leukemia (CML) cells, cisplatin-resistant J82 urothelial carcinoma cells and cisplatin-resistant 2102EP germ cell tumor cells [28]. Here, could show that meriolin 16 and 36 displayed a strong cytotoxic potential in different human leukemia and lymphoma cell lines (HL60, HPBALL, Jurkat, K562, KOPTK1, MOLT4, Ramos, and SUPB15), as well as in primary patient-derived lymphoma and leukemia cells (Fig. 1).

Meriolin 16 was synthesized with an additional methoxy group in order to enhance the potential of its parental compound meriolin 31 [22] (Supplementary Fig. 2B). In a previous study we investigated in detail the apoptosis signaling of meriolin 16 and 36. Thus, we showed that apoptosis induction by both derivatives was independent of death receptor signaling but required caspase-9 and Apaf-1 as central mediators of the mitochondrial death pathway. Meriolin 16 and 36 induced the breakdown of the mitochondrial membrane potential (ΔΨm), mitochondrial release of proapoptotic Smac, processing of the dynamin-like GTPase OPA1, and subsequent fragmentation of mitochondria. Intriguingly, both derivatives were able to activate the mitochondrial apoptosis pathway in Jurkat cells overexpressing the antiapoptotic Bcl-2 protein [28].

In the present study, we focussed on the effect of meriolin 16 and 36 concerning their kinase inhibition profile, binding mode to CDK1, 2 and 9, effect on cell cycle cell progression and proliferation, as well as targeting of gene transcription via inhibition of CDK9. Using a kinome screen, almost all CDKs (such as CDK1, 2, 3, 5, 7, 8, 9, 12, 13, 16, 17, 18, 19, 20) could be identified as targets of meriolin 16 and 36. However, both meriolins displayed different specificity and selectivity, with meriolin 16 being more specific for CDKs amongst the 335 kinases tested and less potent concerning inhibition of CDK4 and 6 (Fig. 2). These results are consistent with previous reports on other meriolin derivatives, which have shown that these compounds are CDK inhibitors with a pronounced cytotoxic, anti-proliferative and anti-tumor activity [16, 17, 19, 22, 24, 25, 61].

We investigated the binding mechanism of the two novel meriolin derivatives against CDK proteins by molecular docking and molecular dynamics simulations. The binding modes were similar to those of previously described indole-containing kinase inhibitors [17]. The high degree of conservation of the active site of CDKs could explain why these compounds are active against different CDKs and, thus, exert biological effects on different cellular processes commonly associated with cancer progression. Nonetheless, small differences in protein structures could influence the kinetics of drug binding by altering the conformational dynamics [62, 63]. Therefore, the pharmacodynamic properties of meriolins might differ among the targeted CDKs. Besides observing a binding mode similar to previous ones, our studies revealed the possibility of an alternative binding mode for meriolin 36. The amino-pyridyl moiety between the indole and the benzene moieties mimics the hydrogen bond acceptor and donor pair also present in the indole group and, thus, is predicted to be able to take on this position. As meriolin 36 has two times a similar pharmacophoric pattern in its structure, it might be a more promiscuous kinase inhibitor since it can fit differently in ATP-binding pockets.

Meriolin-induced CDK2 inhibition was further corroborated by the loss of the phosphorylation of the retinoblastoma protein (RB; at Ser612 and Thr821) which is a direct target of CDK2 (Fig. 4). The functional status of RB protein determines whether a damaged cell undergoes cell cycle arrest or apoptosis [64]. The phosphorylation of RB protein at position Ser612 was analyzed, since this phosphosite has been shown to be phosphorylated by CDK2/cyclin A in late S-/G_2_-phase [65] and might be responsible for the observed meriolin induced G_2_-arrest (Fig. 5). In addition, Thr821 of RB was analyzed, since it is known to be phosphorylated by CDK2/cyclin E in early S phase. Distinct results were obtained for the two different derivatives. Meriolin 16 induced the loss of the phosphorylations (Ser612 and Thr821) at both concentrations (0.1 and 1 µM), whereas meriolin 36 induced an increase of the phosphorylation at Ser612 and no notable change for Thr821 at both concentrations (0.1 and 1 µM). This was probably due to the different IC_50_ values, since meriolin 16 exhibits an IC_50_ value which is lower (0.03 µM) compared to meriolin 36 (3.49 µM) in Ramos lymphoma cells (Fig. 7B). This leads to an insufficient phosphorylation status of the RB protein, which in turn influences E2F-dependent transcription. When the RB protein is mono- or hyperphosphorylated it binds to E2F-dependent genes that regulate cell cycle progression. As a result, essential genes for S phase or G_2_ phase are not transcribed and the cell cycle arrests.

Meriolin mediated inhibition of cell cycle dependent CDKs was obviously responsible for the reduction in cell cycle progression and proliferation (Fig. 5). Intriguingly, even non-lethal concentrations were sufficient for impeding the cell cycle. The tested meriolins apparently induced cell cycle arrest at the G_2_/M phase (Fig. 5A). Similar results have been shown for other meriolin derivatives, such as meriolin 5 and 15, that also induce cell cycle arrest in G_2_/M [25]. Other CDK inhibitors, as early pan-CDK inhibitors (such as flavopiridol) are known to induce both G_1_ and G_2_ arrest [37, 66]. Dinaciclib was also shown to induce cell cycle arrest in G_2_/M phase and to promote apoptosis [67]. In addition to cell cycle arrest, these CDK inhibitors including meriolins reduced proliferation already at non-lethal concentrations. Thus, the proliferative capacity of Ramos cells decreased to 40–70% under treatment with CDK inhibitors (Fig. 5B).

Beside CDKs involved in cell cycle control, meriolin 16 and 36 also potently inhibited CDK9 (Fig. 2C–E) that activates RNA polymerase II by phosphorylation. Accordingly, both meriolin derivatives inhibited de novo RNA-synthesis and transcription (Fig. 6D, E). CDK9/cyclin T is a regulator of transcriptional elongation and termination. CDK9/cyclin T phosphorylates RNA polymerase II at Ser2 at its C-terminal domain [44, 68–70]. Interestingly, meriolin 16 induced the decrease of CDK9 and cyclin T1 levels over time (Fig. 6B, C). Moreover, RNA polymerase II suppression via CDK9 inhibition was shown to result in a block of transcriptional elongation resulting in suppression of anti-apoptotic short-lived proteins such as Mcl-1 [16, 71–73]. Intriguingly, Cidado et al. could show that the selective CDK9-inhibitor AZD4573 induced a rapid downregulation of Mcl-1 mRNA, followed by a downregulation of Mcl-1 on protein level within 4 h, whereas the protein expression of Bcl-2 and Bcl-xL remained rather stable over 9 h [72]. In this context we could show that overexpression of Bcl-2 has no effect on meriolin-induced apoptosis, while overexpression of Bcl-xL and Bax-/Bak-deficiency does [28]. This is consistent with the observation that double-knockdown of Bax and Bak or Bak knockdown substantially abrogated CDK9-inhibitor-induced caspase-activation, whereas Bax knockdown was much less effective [72]. Thus, CDK9-inhibition appears to induce apoptosis via Bak by downregulating short-lived Mcl-1. Since Mcl-1 and Bcl-xL can interact with both Bax and Bak (unlike Bcl-2, which only binds to Bax) [74–76], this may explain why overexpression of Bcl-2 does not affect meriolin-induced apoptosis.

Transcriptional CDKs like CDK9 have been shown to be involved in the development of tumorigenesis and correlating abnormal CDK9/cyclin T1 activity has been described in several human malignancies [77]. CDK9 is also deregulated in myeloid leukemia [78] and its overexpression has been reported in CLL, B and T cell precursor-derived lymphoma, and follicular lymphoma and in Burkitt lymphoma abnormal mRNA levels of CDK9/cyclin T1 have been described [78, 79]. Thus, CDK9 inhibition by meriolin 16 (similar to dinaciclib) might serve as a promising approach for cancer treatment – especially of tumors overexpressing antiapoptotic Bcl2.

Finally, we compared the cytotoxic potential of meriolin 16 and 36 to other known CDK inhibitors and could show that meriolin 16 was the most active derivative amongst all meriolins tested—even more potent than meriolin 3 which is the most potent meriolin derivative described so far. Meriolin 16 was active at nanomolar concentrations and was more effective than other pan- or specific CDK inhibitors that are already on their way into the clinic (such as flavopiridol or roscovitine) and almost as potent as dinaciclib, which is already tested in clinical trials, according to the data bank of clinicaltrials.gov (Fig. 7).

Taken together, both meriolin 16 and 36 displayed a pronounced cytotoxic potential in various leukemia and lymphoma cell lines as well as in primary cells isolated from leukemia and lymphoma patients. Using a kinome screen, it could be shown—with exception of CDK4 and 6—that meriolin 16 and 36 potently inhibited all other CDKs tested. Consequently, cell cycle progression and proliferation were blocked. Beside CDKs involved in cell cycle control, meriolin 16 and 36 also potently inhibited CDK9 and subsequent RNA polymerase II mediated transcriptional activity. Finally, the comparison with known CDK inhibitors identified meriolin 16 as a promising candidate that was more cytotoxic than flavopiridol, meriolin 3 and meriolin 36 in Ramos cells and comparably effective as dinaciclib. This renders meriolins as versatile anticancer drugs since they target three different Achilles’ heels of the tumor by (1) inhibition of cell cycle progression and proliferation, (2) reduction of gene transcription, and (3) induction of cell death (i.e., apoptosis).

## Materials and methods

### Meriolins

The semisynthetic meriolin 36 (*N*-benzyl-4-(1*H*-pyrrolo2,3-*b*]pyridine-3-yl)pyridine-2-amine) was synthesized by the group of Prof. Dr. T. J. J. Müller (Institute of Organic Chemistry of Heinrich Heine University Düsseldorf) and described in [18, 22]. Meriolin 16 (4-(4-methoxy-1*H*-pyrrolo2,3-*b*]pyridine-3-yl)pyridine-2,6-diamine) was newly synthesized based on the structure of 31 with an additional methoxy-group and described in [18]. Meriolin 3 was synthesized by others as described and characterized in [16, 17, 25]. All meriolin derivatives were dissolved in DMSO at a 10 mM stock solution and stored at −20 °C.

### Cell lines and culture conditions

Ramos cells (human B cell Burkitt lymphoma) were kindly provided by Michael Engelke (Institute of Cellular and Molecular Immunology, University Hospital Göttingen, Germany). Jurkat cells (acute T cell leukemia) were obtained from DSMZ (Deutsche Sammlung von Mikroorganismen und Zellkulturen; ACC-282). HeLa cells (human cervix carcinoma; #ACC-57), HL60 (human acute myeloid leukemia; #ACC-3), HPBALL (human T cell acute lymphoblastic leukemia; #ACC-483), MOLT4 (human T cell acute lymphoblastic leukemia; #ACC-362), K562 (human chronic myeloid leukemia; #ACC-10) and SUPB15 (human B cell acute lymphoblastic leukemia; #ACC-389) were obtained from DSMZ. KOPTK1 (human T cell acute lymphoblastic leukemia; CVCL-4965) were kindly provided by Oskar Haas (Children’s Cancer Research Institute, St. Anna Children’s Hospital, Vienna, Austria). All cell lines were maintained at 5% CO_2_, 37 °C, and stable humidity in the following cell culture media. The suspension cells were maintained in RPMI media with 10% FCS, 10 mM HEPES, 100 U/ml penicillin and 100 µg/ml streptomycin. HeLa cells were cultured in high-glucose Dulbecco’s Modified Eagle’s medium (DMEM) supplemented with 10% FCS, 10 mM HEPES, 100 U/ml penicillin and 100 µg/ml streptomycin.

### Malignant primary patient cells

Informed consent was obtained from all patients before isolation and storage of primary tumor cells in line with the Declaration of Helsinki and the Department of Health and Human Services Belmont Report. The study was approved by the ethics committee of the Medical Faculty Heidelberg, Germany (S-206/2011 and S-356/2013). For chronic lymphocytic leukemia, mononuclear cells were isolated from peripheral blood using a Ficoll gradient (GE Healthcare). Primary cells from other lymphoma entities were isolated as previously described [80]. All cells were cryopreserved after isolation and thawed as previously described [81].

### Reagents

The following reagents were purchased from Sigma-Aldrich: R547 (#SML1254), flavopiridol (F3055), roscovitine (#557360), SNS-032 (#SML2218) and meriolin 3 (#445821). The following reagents were purchased from Selleckem: dinaciclib (#S2768) and Q-VD-OPH (#S7311). Zotiraciclib was purchased from AbMole (#M10327). Staurosporine was purchased from Biozol (#S-9300).

### Cell viability

The resazurin reduction assay, which is also known as AlamarBlue® assay, was used for the determination of cell viability in all cytotoxicity determinations shown in the present work, as previously described [22]. Briefly, cells were seeded at a specific density depending on the incubation time (24 h: 1 × 10^6^ cells/ml) in a 96-well plate and incubated with increasing compound concentrations (0.01–30 µM). After the specified treatment time, resazurin (Sigma, #R7017) was added to a final concentration of 40 µM. After 120 min of incubation, the fluorescence of resorufin (535/590 nm) was measured with a microplate spectrophotometer (Synergy Mix plate reader). DMSO (0.1% v/v) was used as negative control and staurosporine (2.5 µM) as internal positive control. The viability of control cells was set to 100% and all other values were normalized to the control condition, the dose-response curves were then fitted with Prism v7.01 (GraphPad software, La Jolla, CA, USA). The reduction of resazurin to resorufin is proportional to aerobic respiration; therefore, it is used as a measure for the cell viability and cytotoxicity of a tested compound.

### Analysis of apoptotic cell death and cell cycle

DNA content during cell cycle and hypodiploid apoptotic nuclei were measured by the method of Nicoletti et al. [41]. Nuclei were prepared by lysing cells in a hypotonic lysis buffer (1% sodium citrate, 0.1% Triton X-100, and 50 µg/ml propidium iodide) and then analyzed by flow-cytometry. Prepared nuclei were measured on linear mode to clearly differentiate G_1_-, S-, G_2_/M-Phase, and hypodiploid nuclei (HN) for cell cycle measurements. Hypodiploid nuclei were measured in logarithmic mode for apoptosis determination. Flow-cytometry analysis was performed on an LSRFortessa^TM^ (Becton, Dickinson, Heidelberg, Germany) and data analysis was performed using FlowJo_V10 (BD Biosciences).

### Immunoblotting

Cells were seeded at a density of 2 × 10^6^ cells, treated as specified and harvested by centrifugation at 3000 rpm for 5 min, followed by freezing in liquid nitrogen. The cell pellets were thawed on ice and quick-frozen and defrozen in liquid nitrogen for three times, then lysed on ice in lysis buffer (20 mM Tris-HCl, 150 mM NaCl, 1% v/v Triton X-100, 0.5 mM EDTA, 10 mM NaF, 2.5 mM Na_4_P_2_O_7_, protease inhibitor (Sigma, #P2714)) for 30 min, accompanied by vortexing. Subsequently, centrifugation 13,300 rpm for 15 min purified cell lysates from cell debris. The supernatant was transferred to a new tube and the protein concentration was determined by Bradford assay. The samples were diluted with sample buffer. SDS-Page and Western Blot were conducted in accordance with standard workflows. Primary antibodies were diluted in 1x TBS-T supplemented with 0.05% NaN_3_ (without 5% BSA) according to manufacturer’s suggestions. The antibody baths were stored at 4 °C and re-used.

The following primary antibodies were purchased from Thermo Fisher (cyclin A2-ms #MA1-154, 1:1000; CDK1-ms #MA5-11472, 1:100; cyclin B1-ms #MA5-13128, 1:50; cyclin E-rb #MA5-42650, 1:2000; cyclin D3-rb #MA5-32366, 1:1000; CDK9-rb #MA5-32397, 1:1000; cyclin T1-rb #PA5-82177, 1:250; p-Ser612-RB-rb #PA5-64513, 1:1000). The following primary antibodies were purchased from Invitrogen (CDK2-rb #MA5-32017, 1:1000; p-Thr821-RB-rb #44-582G, 1:1000; p-Ser249, Thr252-RB-rb #44-584G, 1:1000). The primary RB-ms antibody was purchased from BD Biosciences (#554136, 1:250), p-Ser2-RNA polymerase II-rb was purchased from Biomol (#A300-654A, 1:1000). The loading controls β-Actin-ms (#A5315, 1:20000) and Vinculin-ms (#V9131, 1:2000) were purchased from Sigma-Aldrich and GAPDH-ms was purchased from Abcam (#ab8245, 1:5000).

IRDye®-conjugated secondary antibodies from LICOR® Bioscience (680RD Donkey anti-Mouse (#926-68072), 800CW Donkey anti-Mouse (#926-32212), 800RD Donkey anti-Rabbit (#926-32213) and 680RCW Donkey anti-Rabbit (#926-68073)) were diluted 1:20.000 (680RD) or 1:10.000 (800RD) in 1x TBS-T for the detection of target proteins on PVDF membrane using LI-COR Odyssey® imaging system.

### Immunopurification

For immunopurification (IP) the Dynabeads^TM^ Protein G Immunoprecipitation kit from Invitrogen (#10007D) was used. For IP, 2 × 10^6^ cells were seeded in a 24-well plate and treated with the appropriate compound. After incubation, cells were lysed with NP-40 containing lysis buffer (Hepes-NaOH 50 mM pH 7.5, NaCl 150 mM, NP-40 1% v/v, MgCl_2_ 2.5 mM, 1x Protease inhibitor (cOmplete^TM^, EDTA-free Protease Inhibitor Cocktail), 1x Phosphatase Inhibitor (PhosSTOP^TM^)) for 30 min. A protein content of 50 µg per IP was used, the protein concentration was measured by Bradford assay (Bio-Rad #5000006) with the photometer. Protein G is conjugated to the magnetic Dynabeads^TM^. In the first step, the Dynabeads^TM^ were incubated for 10 min with the specific RB antibody (Abcam #ab181616; rabbit), which was used in a dilution of 1:80. The antibody bound to the conjugated protein G and a bead-antibody complex was formed. By placing the tube on a magnet, the beads were easily separated from the solution and the supernatant could be removed. The bead-antibody complex was washed once with Ab binding and washing buffer. In the second step, the cell lysate was incubated for 10 min with the bead-antibody complex to allow the RB protein from the lysate to bind to the antibody. The bead complex was washed several times with washing buffer by placing the tube on the magnet and removing the supernatant. Elution was performed for 20 min by adding the elution buffer. After elution, 6 x Laemmli buffer was added to the samples and they were heated up at 37 °C for 30 min. Samples were loaded onto a 10% SDS gel and gel electrophoresis followed by immunoblotting was performed. IgG control was obtained from Sigma #14506.

### Statistical analysis of Western blots

The density of the individual protein band was determined using Image Studio^TM^ Lite Version 5.2. The density of each protein was divided by the average of all bands of this protein. The ratios of the protein of interest were normalized to the ratio of the respective loading control. To determine the fold change, each normalized ratio was divided by the normalized band of the control line (untreated cells). Fold change is 1 for the control line, *n* ≥ 2. The results are shown as mean ± standard deviation and were visualized with GraphPad Prism 7.

### Microscopy-based analysis of EdU-incorporation

The EdU-assay, utilizing the EdU-click 488 kit from Baseclick GmbH (#BCK-TCell-FC488) was performed according to manufacturer’s instructions. The EdU-assay is employed to measure the incorporation of 5-ethynyl-2’-deoxyuridine (EdU) into DNA, providing a means to assess cell proliferation. Cell proliferation involves the synthesis of new DNA during the S phase of the cell cycle. When cells are exposed to 5-EdU, they incorporate the compound into their DNA at thymidine bases during S phase. A fluorophore-labeled azide reacts with the incorporated EdU, enabling its detection through microscopy (sigmaaldrich.com). The experimental process involves seeding cells and allowing them to grow to the desired density, applying the indicated treatments, and subsequently adding EdU to the media. After fixation and permeabilization, the EdU detection, along with DAPI-stained nuclei, is carried out using fluorescence microscopy.

### Microscopy-based analysis of EU-incorporation

The Invitrogen Click-iT® RNA imaging kit (#C10329) was performed according to manufacturer’s instructions. Briefly, transcriptional activity and newly synthesized RNA was measured by incorporating 5-Ethynyl-uridine (EU). EU is a nucleoside analog of uracil, which is used to directly image spatially and temporally nascent global RNA transcription. Once incorporated into the RNA, EU can react with an azide-containing detection reagent, forming a stable triazole ring coupled to a fluorescent dye. Therefore, detection of EU incorporation serves as a measurement for de novo RNA-synthesis. In summary, cells were plated the day before the experiment, treated with specific substances for the indicated duration, and then exposed to EU for 1 h to allow its incorporation. EU incorporation was monitored following the manufacturer’s instructions. Subsequently, the cells were fixed and permeabilized, and the detection of EU, along with DAPI-stained nuclei, was carried out using fluorescence microscopy.

### BrdU assay

The Bromodeoxyuridine (BrdU) Cell Proliferation kit from Millipore (#QIA58-1000TEST) was used to measure proliferation of cells. The manufacturer protocol has been optimized as described in the following. 1 × 10^4^ cells per well were seeded in a 96-well plate. The cells were pre-incubated with the compounds of interest for 1 h. Following this, the BrdU solution was added and the cells were incubated for a total of 24 h. BrdU is an analog of the nucleoside thymidine and is incorporated into the DNA of dividing cells during replication. Culture medium was removed and cells were fixed and denatured with Fixative/Denaturing solution for 30 min at room temperature. Since suspension cells were used, they had to be centrifuged at 1200 rpm for 5 min after each following step. After, the anti-BrdU-antibody was incubated for 1 h at RT. Cells were washed three times with washing buffer. The secondary antibody, which is coupled to HRP (horseradish peroxidase) was incubated for 30 min at RT. Again, cells were washed three times with washing buffer.

Then, the substrate solution was added, that contained TMB (3,3’,5,5’-Tetramethylbenzidine), which is oxidized by HRP. Oxidation of TMB causes a color change from colorless to blue. The intensity of color change is correlated with the number of bound peroxidases, which depends on the quantity of primary antibodies and therefore on the content of the incorporated BrdU. If the cells are not proliferating, no BrdU is incorporated, resulting in a weak signal.

After 15 min incubation time the stopping reagent was added, causing a pH change and resulting in a color change from blue to yellow. The colored reaction product was quantified at 450 nm using a spectrophotometer. For all measurements, three technical replicates were made. The control was set to 100% proliferation, and the treated cells were normalized to the control. The results were visualized using GraphPad Prism 7.

### Molecular modeling of CDK-meriolin complexes

The structures of meriolin 16 and 36 were prepared for docking using LigPrep, assigning the most likely protonation state at pH 7 using Epik. The crystal structures of CDK1 (PDB: 4Y72), CDK2 (PDB: 3BHU, 3BHT) and CDK9 (PDB: 3TNH), and the inactive form of CDK2 (PDB: 1HCK) were prepared using the Schrodinger Preparation wizard [82]. The protonation state of titratable residues was assigned with Propka [83]. Dockings were carried out using Glide in extra precision mode [84] with a grid cell of 35 Å placed in the active site, using as reference the crystallized ligand on the CDK2 structures (both in its active and inactive form) and OPLS_2005 as force field. Three poses per ligand were generated.

Partial charges for meriolin 16 and 36 were obtained by performing a geometric optimization at the Hartree-Fock 6-31G* theory level with Gaussian16 [85] and then deriving atomic point charges through the RESP fitting method in Antechamber [86]. Simulations of the protein-ligand complexes were performed using the Amber22 suite of simulation software with the ff19SB force-field for proteins and the general amber force-field 2 (GAFF2) for ligands. Complexes were solvated on a TIP3P truncated octahedron with a 14 Å distance towards the edge of the box. All simulations were performed using the Amber22 suite of software. Long-range interactions were treated with the GPU-accelerated implementation of the Particle Mesh Ewald Method [87], using a cut-off of 10 Å. All bonds involving hydrogen atoms were constrained using the SHAKE algorithm.

The systems were minimized in three rounds using a mixture of 500 steps of steepest descent, followed by 2000 steps of conjugate gradient. In the first round, positional restraints of 5.0 kcal mol^−1^ were placed on all heavy atoms, then the process was repeated with a lighter restraint of 0.1 kcal mol^−1^ and finally with no restraints. The systems were thermalized from 0 to 100 K in a window of 25 ps, using the Langevin thermostat with a collision coefficient of 1.0 ps^−1^ under NVT conditions. Afterward, the systems were heated to 300 K over a window of 250 ps, under NPT conditions using the Berendsen barostat with isotropic position scaling. All previous steps were performed with a positional restraint of 1 kcal mol^−1^. The restraints were removed gradually in 0.2 kcal mol^−1^ steps over a total window of 250 ps. Unrestrained production runs were carried out for 1 µs under NPT conditions. Five independent replicas for each system were performed.

Per-atom root mean square fluctuations were calculated using cpptraj [88] using the *atomicfluct* command after aligning the protein residues to the first frame using the *rmsd* function. Volumetric grids of the ligand occupancies were determined using the *grid* command in cpptraj, with a spacing of 0.5 Å.

### CDK activity assays (CDK1/cyclin B1, CDK2/cyclin A2, and CDK9/cyclin T)

The activity of specific CDKs and their cyclins was assessed using CDK Kits from BPS Bioscience (CDK1/cyclin B1 #79597; CDK2/cyclin A2 #79599; CDK9/cyclin T #79628). The assay was conducted following the manufacturer’s guidelines. In summary, the CDK/cyclin complexes, as purified recombinant enzymes, were allowed to react with CDK substrate peptide, ATP, and kinase buffer in the presence or absence of meriolins. The reaction was initiated by adding diluted CDK/cyclin enzyme to the wells and incubating for 45 min at 30 °C. After the incubation, the Kinase-Glo-Max® reagent was added as a detection reagent, and the resulting luciferase reaction produced luminescence, which was inversely correlated with the kinase activity.

The luminescent CDK/cyclin assay is a method that quantifies the amount of ATP remaining in the solution after a kinase reaction. Luminescence was measured using the Synergy Mix microplate reader as an endpoint measurement. Data analyses were performed according to the manufacturer’s instructions, and statistical analysis was carried out using one-way ANOVA, with corrections made by Bonferroni’s multiple comparison test. The significance level was indicated as ****p ≤ 0.0001.

### Kinome screening by Reaction Biology

The Kinome Screening was performed by Reaction Biology Europe GmbH (http://reactionbiology.com; Freiburg i. Br., Germany). After treatment with meriolin 16 (tested concentrations: 0.03 µM and 0.3 µM), meriolin 36 (tested concentrations: 0.3 µM and 3 µM) and an inactive structurally related meriolin derivative (17, tested concentrations: 0.3 µM and 3 µM), the company measured the activity of 335 wild-type protein kinases as recombinant purified active proteins. The inactive derivative (meriolin 17) was chosen to rule out false positive results. A kinase inhibition profile of the three meriolins was determined by measuring residual activity at two concentrations each in single values in 335 wild-type protein kinase assays.

The residual activity was calculated as:

$${{{Res}}}.{{{Activity}}}\left( \% \right)=100\,x\,(\frac{\left({{{Signal}}\; {{of}}\; {{compound}}}-{{{low}}\; {{control}}}\right)}{{{{high}}\; {{control}}}-{{{low}}\; {{control}}}})$$

The selectivity score was calculated as:

$${{{Selectivity}}\; {{Score}}}=\frac{\left({{Count}}\; {{of}}\; {{data}}\; {{points}} \,<\, 50 \% \right)}{({{{Total}}}\; {{number}}\; {{of}}\; {{data}}\; {{points}})}\,$$

### Replicates and statistical analysis

Experiments were replicated at least two times and only representative data are shown. Error bars indicate standard deviation (SD). All statistical analyses were performed using Prism v7.01 (GraphPad Software, La Jolla, CA, USA). Statistical analyses were only performed in the case of *n* = 3.

### Supplementary information

Supplemental Material: Supplemental Figures and Tables

Supplemental Material: Original Western blots

## Data Availability

Data were generated by the authors and included in the article.
