# Supplementary material for: Novel meriolin derivatives potently inhibit cell cycle progression and transcription in leukemia and lymphoma cells via inhibition of cyclin-dependent kinases (CDKs)
Source: Cell Death Discov. 2024 Jun 11;10:279. doi: 10.1038/s41420-024-02056-6 (PMC11167047; doi:10.1038/s41420-024-02056-6)
Supplement: Supplementary file 1 — Supplemental Material: Supplemental Figures and Tables [file 41420_2024_2056_MOESM1_ESM.pdf]

Supplemental Information

Supplemental Figures

Supplemental Figure 1: CDK activity and periodic expression of Cyclins are the main regulators of cell cycle progression and act via phosphorylations on key mediators of the cell cycle

Supplemental Figure 2: Structures of meridianins and variolins – the parental compounds of meriolins, the four meriolin derivatives analyzed in this and comparable CDK inhibitors

Supplemental Figure 3: Active site residue conservation on CDK1, 2 and 9

Supplemental Figure 4: Docking meriolin 16 and meriolin 36 against all 3 CDKs yields similar binding poses

Supplemental Figure 5: Alternative binding modes for meriolin derivatives

Supplemental Figure 6: Cyclin D3, p-Ser249/p-Thr252-RB and CDK1/Cyclin B1 protein levels over time

Supplemental Figure 7: Representative Immunoblots of 8, 12 and 16 h treatment with meriolin 16 and meriolin 36 in 0.1 and 1 µM and R547 in 1 µM over time

Supplemental Figure 8: Immunopurification (IP) of RB after 4 and 24 h meriolin 16 treatment

Supplemental Tables

Supplemental Table 1: Kinome profiling (33PanQinaseTM) of meriolin 17 at 0.3 and 3 µM, meriolin 36 at 0.3 and 3 µM and meriolin 16 at 0.03 and 0.3 µM each against 335 wild-type protein kinases in singlicate measurement

Supplemental Table 2: Overview of CDK inhibitors of which some are commercially available and in use in pre-clinical trials, clinical phases or are applied in clinical treatment

Supplemental Table 3: Predicted ADME properties for meriolin 16 and 36

Supplemental References

Supplemental Figures

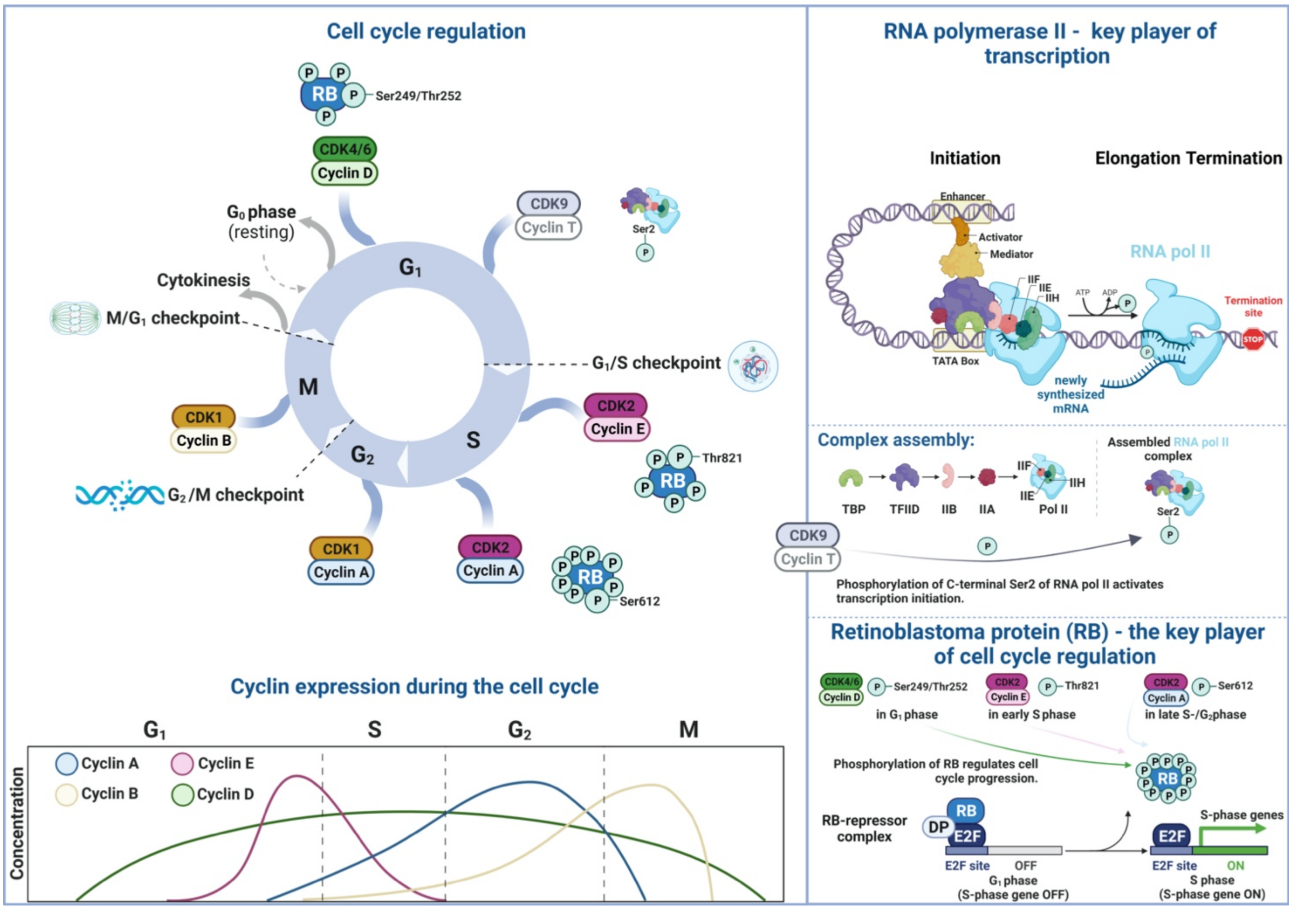

Suppl. Figure 1: CDK activity and periodic expression of Cyclins are the main regulators of cell cycle progression and act via phosphorylations on key mediators of the cell cycle.

The cell cycle is separated in G_1, S, G_2 and M phase. The depicted CDKs in complex with their Cyclin binding partners mediates progression of the cell cycle by phosphorylating their downstream targets. These downstream targets are for example RNA polymerase II (RNA pol II) or Retinoblastoma protein (RB), which get phosphorylated by different CDK/Cyclin complexes: CDK9/Cyclin T phosphorylates RNA pol II at Ser2 in G_1 to initiate transcription, whereas CDK1/Cyclin B phosphorylates RNA pol II at Ser5 in M to inhibit transcription. The RB protein is the main regulator protein for gene expression, since it suppresses E2F-dependent gene expression during G_1 in its hypo- or mono-phosphorylated form in a complex with the transcription factor DP. This complex dissociates from E2F regulated genes, as soon as RB gets phosphorylated by CDK4/6/Cyclin D on Ser249/Thr252. Further phosphorylations are added on the protein by CDK2/Cyclin E in early S phase on Thr821 or CDK2/Cyclin A in late S-/G_2 phase on Ser612 and many more (since the RB protein has 14 *in vivo* CDK phosphorylation sites in total) [1]. (This figure was created with Biorender.com according to figures in [2-9]).

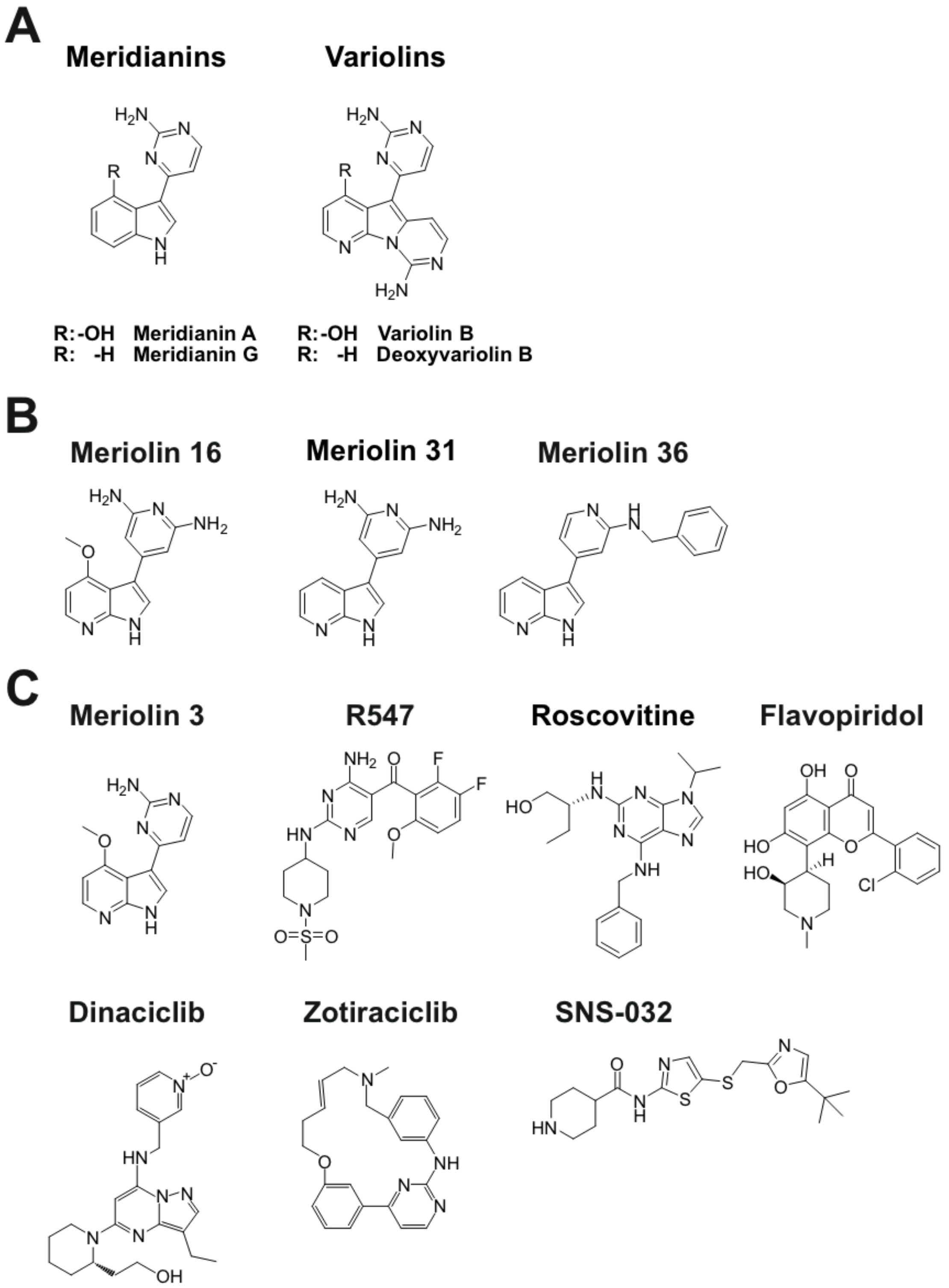

Suppl. Figure 2: Structures of meridianins and variolins – the parental compounds of meriolins, the four meriolin derivatives analyzed in this and comparable CDK inhibitors.

(A) The natural compounds meridianins and variolins inspired the synthesis of meriolins as semisynthetic hybrid structures. (B) Structure of the biologically active meriolin derivatives 16, 31 and 36. (C) The meriolins are compared to known CDK inhibitors of which some are already in clinical trials. (Structures shown in A-B were drawn according to structures in [10-12], others according to their Pubchem IDs: meriolin 3 (23727981), R547 (91618035), roscovitine (160355), flavopiridol (527969), dinaciclib (46926350), zotiraciclib (16739650) and SNS-032 (3025986) in ChemDraw).

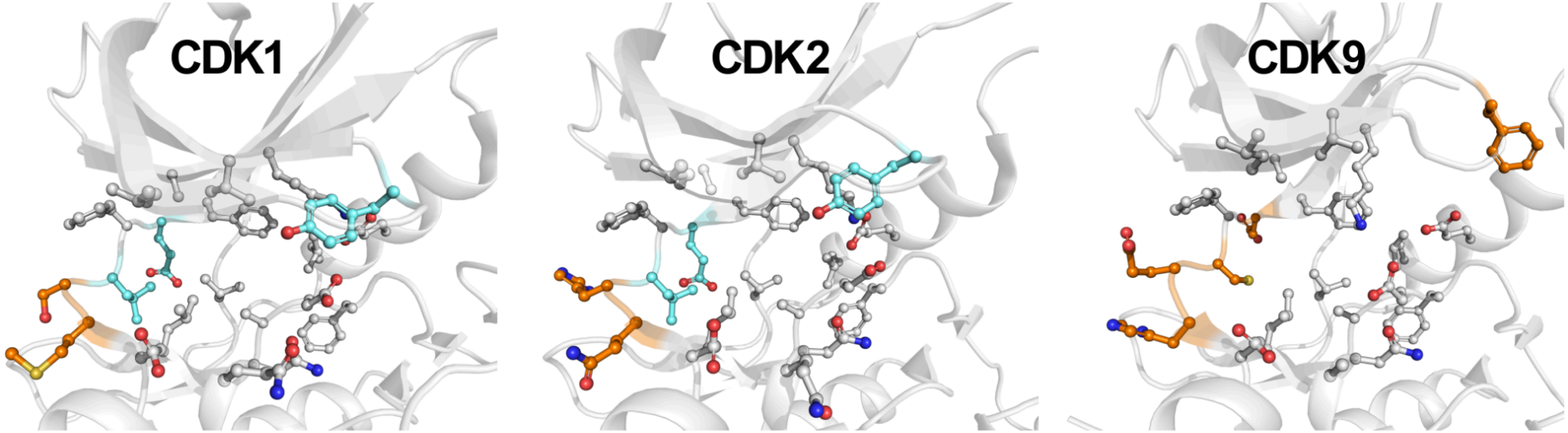

Suppl. Figure 3: Active site residue conservation on CDK1, 2 and 9.

The ATP binding pocket residues of CDK1, 2, and 9 are depicted as sticks and colored according to their conservation. Residues identical across the three proteins are colored white, residues observed in two proteins are colored in cyan, and unique residues are colored in orange.

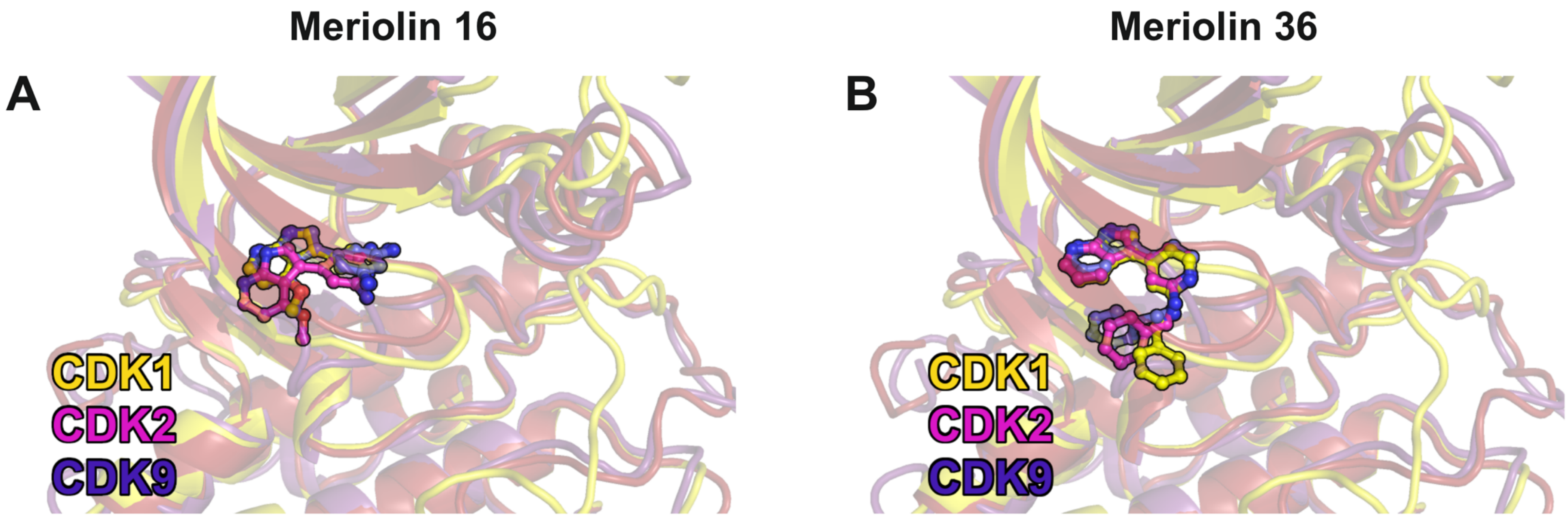

Suppl. Figure 4: Docking meriolin 16 and meriolin 36 against all 3 CDKs yields similar binding poses.

Docking results for meriolin 16 **(A)** and meriolin 36 **(B)** against CDK1 (yellow), CDK2 (magenta) and CDK9 (purple).

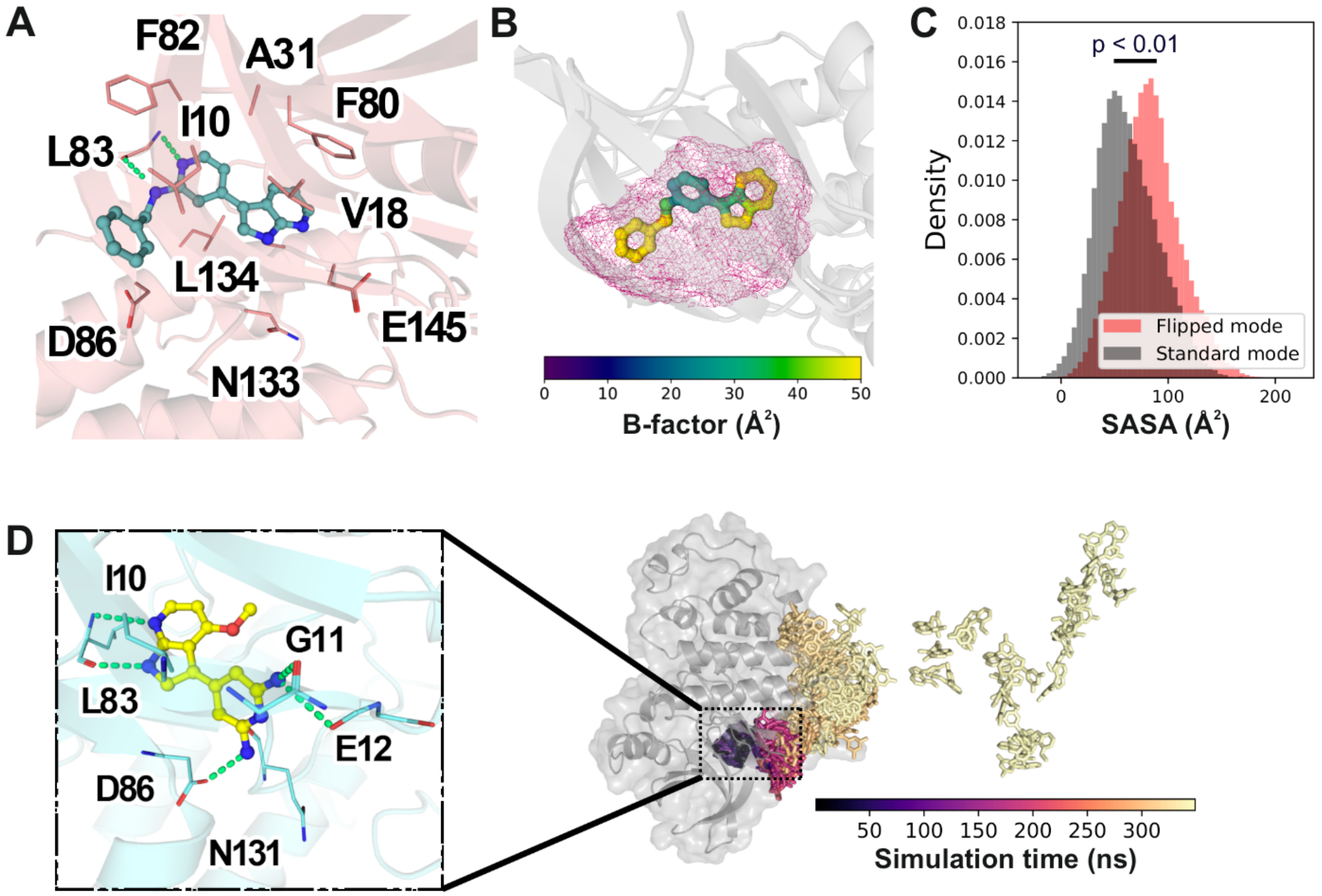

Suppl. Figure 5: Alternative binding modes for meriolin derivatives.

(A) "Flipped" binding mode of meriolin 36 against CDK2. **(B)** Mobility of meriolin 36 throughout the course of the molecular dynamics simulations expressed as volumetric occupancy (pink mesh) and per-atom B-factor (color code according to the given scale; higher values indicate higher mobility). **(C)** Histogram showing the solvent accessible surface (SASA) of meriolin 36 on its standard mode (black) and the flipped mode (red), *p*-value indicated on top. **(D, left panel)** Binding mode of meriolin 16 against inactive CDK2, the green dashed lines represent electrostatic interactions between the compounds and the protein. **(D, right panel)** Overlay of the position of meriolin 16 (shown as sticks) through the simulation couse (indicated by the color scale).

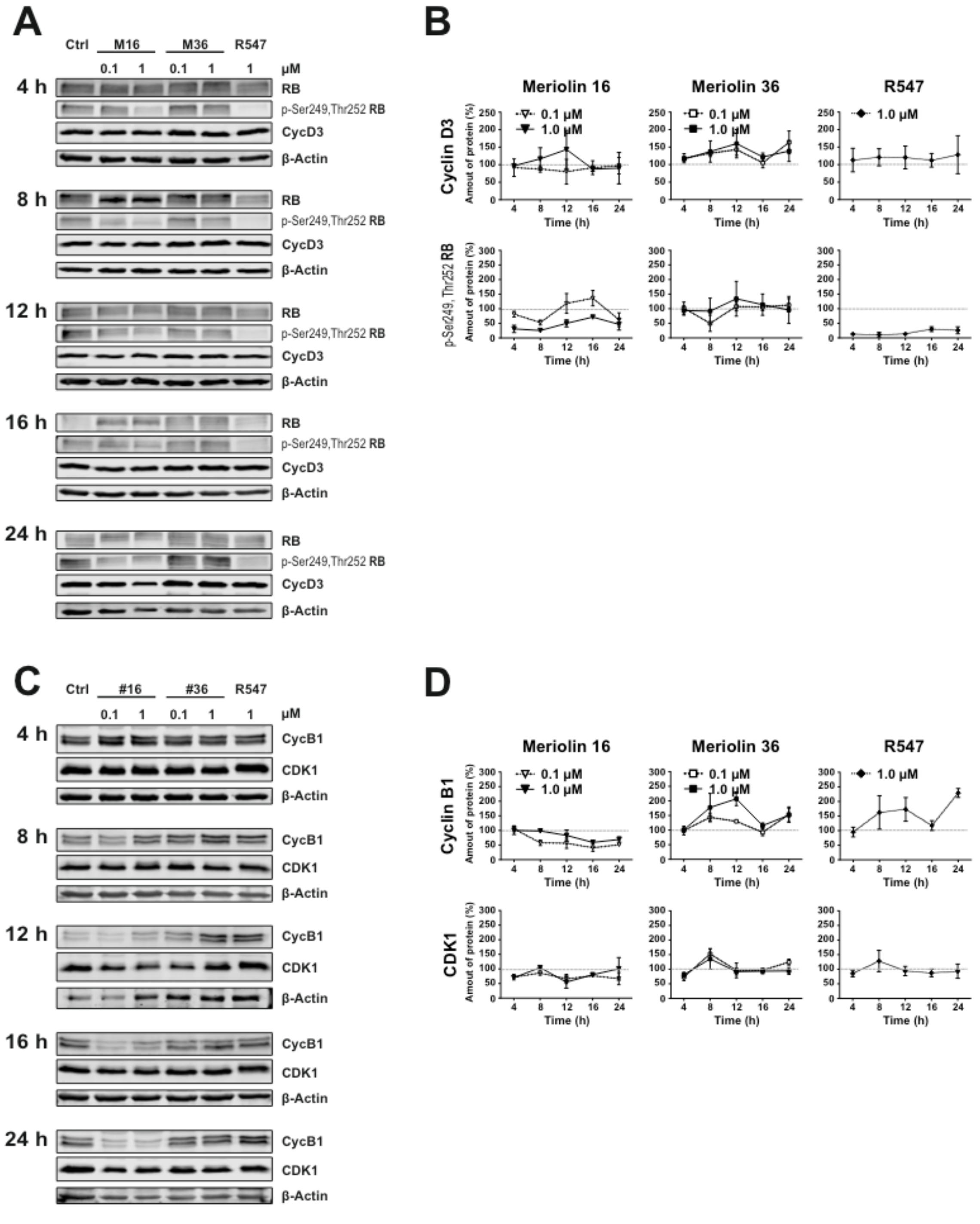

Suppl. Figure 6: Cyclin D3, p-Ser249/p-Thr252-RB and CDK1/Cyclin B1 protein levels over time.

(A-D) Ramos cells were treated for 4, 8, 12, 16 and 24 h with untreated cells as control, meriolin 16 (0.1 and 1 µM) and meriolin 36 (0.1 and 1 µM) and with R547 (1 µM as comparative CDK inhibitor). Representative immunoblots of three independent biological replicates for each time point are shown for the detection of **(A)** Cyclin D3 and phospho-RB (p-Ser249/p-Thr252 RB), **(C)** CDK1 and Cyclin B1. (β-Actin served as loading control). **(B)** and **(D)** show the quantification (≥ 2 independent biological replicates) of the kinetics of the immunoblots for Cyclin D3, phospho-RB (p-Ser249/p-Thr252 RB), CDK1 and Cyclin B1. Error bars = Mean ± SD values of ≥ 2 independent biological experiments are shown.

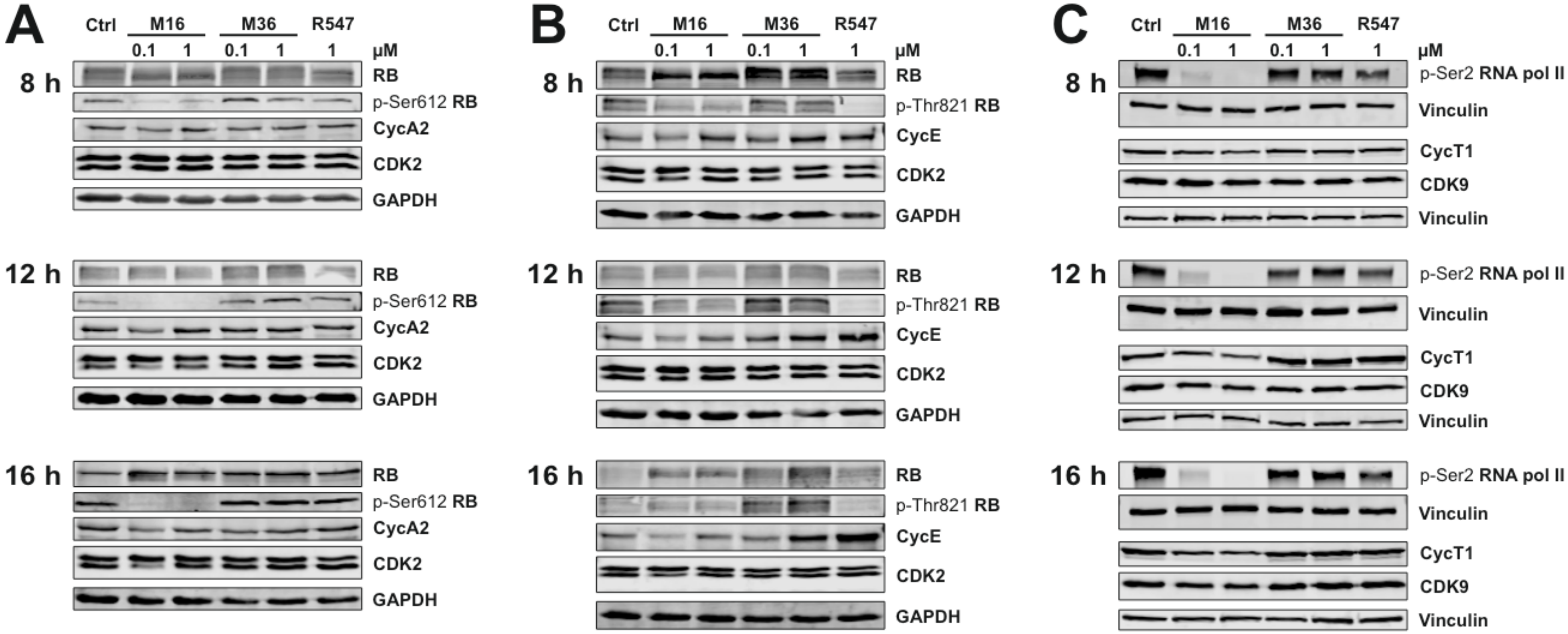

Suppl. Figure 7: Representative Immunoblots of 8, 12 and 16 h treatment with meriolin 16 and meriolin 36 in 0.1 and 1 µM and R547 in 1 µM over time.

(A-C) Ramos cells were treated for 4, 8, 12, 16 and 24 h with media as control, meriolin 16 (0.1 and 1 µM) and meriolin 36 (0.1 and 1 µM) and with R547 (1 µM as comparative CDK inhibitor). Shown are n ≥ 2 biological replicates, which was used for quantifications shown in Figure 6 and 7. The immunoblots after 4 and 24 h are shown in Fig. 5 and 7 of the main text.

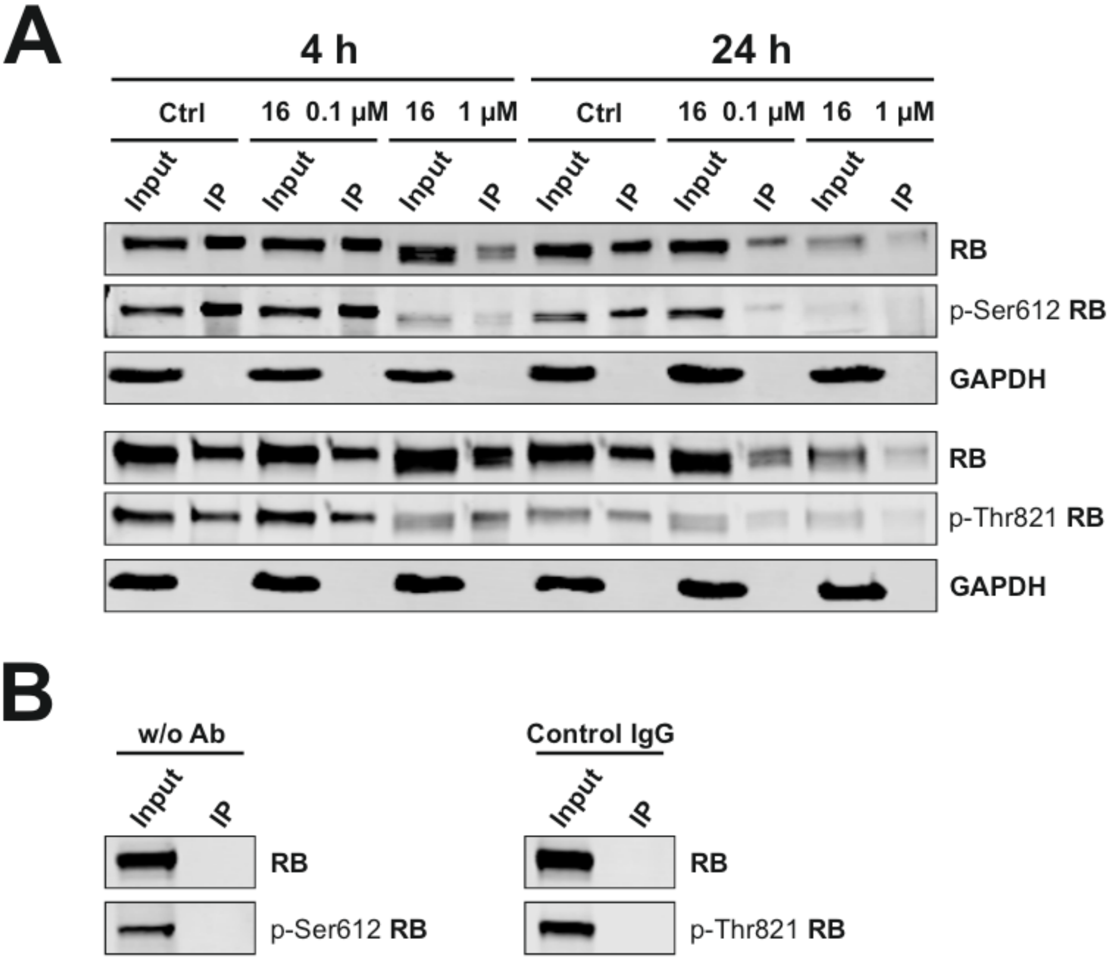

Suppl. Figure 8: Immunopurification (IP) of RB after 4 and 24 h meriolin 16 treatment.

(A) Ramos cells were treated with 0.1 and 1 µM meriolin 16 for 4 and 24 h, subsequently immunopurification was performed against total RB protein (Anti-RB (rabbit), from Abcam #ab181616, in a dilution of 1:80 (18.81 µg/mL)) using the Dynabeads^{TM} Protein G immunopurification kit from Invitrogen. As input 50 µg protein content were used per IP. The protein level of the total RB protein and the phosphosites p-Ser612 (Thermofisher #PA5-64513) and p-Thr821 (Invitrogen #44-584G) were analyzed by immunoblotting. **(B)** IP controls without antibody and IgG control. IP was performed without antibody (w/o Ab) and with an unspecific IgG binding antibody (Control IgG) to represent non-specific binding partners.

Supplemental Tables

Suppl. Table 1: Kinome profiling (^{33}PanQinase^{TM}) of meriolin 17 at 0.3 and 3 µM, meriolin 36 at 0.3 and 3 µM and meriolin 16 at 0.03 and 0.3 µM each against 335 wild-type protein kinases in singlicate measurement.

Shown is the residual kinase activity (% of control) for each treatment. (# = kinase number, * the classification of protein kinase families was performed according to [13]: AGC (containing PKA, PKG and PKC families); CAMK (containing Calcium/Calmodulin-dependent protein kinases); CK1 (Casein kinase 1-like); CMGC (containing CDK, MAPK, GSK3 and CLK families); TK (Tyrosine Kinases); TKL (Tyrosine Kinase-like) and STE (Homologs of Yeast Sterile 7, Sterile 11 and Sterile 20 Kinases). Selectivity Scores (< 50% residual activity): meriolin 17 (0.3 µM) 0.009, meriolin 17 (3 µM) 0.069, meriolin 36 (0.3 µM) 0.397, meriolin 36 (3 µM) 0.752, meriolin 16 (0.03 µM) 0.140, meriolin 16 (0.3 µM) 0.284.

#	Kinase Name	Kinase Family*	M17 0.3 µM	M17 3 µM	M36 0.3 µM	M36 3 µM	M16 0.03 µM	M16 0.3 µM
1	**ABL1**	**TK**	106	106	80	26	103	99
2	**ABL2**	**TK**	89	85	56	18	95	91
3	**ACK1**	**TK**	86	82	59	15	83	72
4	**ACVR1**	**TKL**	86	84	24	1	89	72
5	**ACVR1B**	**TKL**	62	70	43	10	75	69
6	**ACVR2A**	**TKL**	107	86	94	47	103	103
7	**ACVR2B**	**TKL**	74	68	22	5	81	74
8	**ACVRL1**	**TKL**	91	79	43	9	101	89
9	**AKT1**	**AGC**	78	84	89	41	96	70
10	**AKT2**	**AGC**	94	99	102	75	98	79
11	**AKT3**	**AGC**	110	90	93	42	108	82
12	**ALK**	**TK**	92	89	60	14	104	77
13	**AMPKα1**	**CAMK**	109	97	57	9	108	78
14	**ARK5**	**CAMK**	105	90	90	40	92	88
15	**ASK1**	**STE**	89	86	50	15	97	85
16	**AuroraA**	**OTHER**	107	82	81	35	109	107
17	**AuroraB**	**OTHER**	82	55	67	26	85	61
18	**AuroraC**	**OTHER**	81	84	76	41	104	95
19	**AXL**	**TK**	101	86	75	28	101	75
20	**BLK**	**TK**	118	101	88	13	126	95
21	**BMPR1A**	**TKL**	111	97	105	61	94	94
22	**BMPR1B**	**TKL**	86	72	75	45	91	91
23	**BMX**	**TK**	108	112	109	49	118	103
24	**BRAF**	**TKL**	97	95	66	50	94	105
25	**BRK**	**TK**	92	87	66	18	91	85
26	**BRSK1**	**CAMK**	94	82	89	73	91	97
27	**BRSK2**	**CAMK**	112	96	112	61	111	92
28	**BTK**	**TK**	97	81	103	77	114	96
29	**BUB1B**	**OTHER**	94	86	89	73	88	81
30	**CAMK1D**	**CAMK**	98	70	70	38	102	81
31	**CAMK2A**	**CAMK**	107	93	26	2	103	81
32	**CAMK2B**	**CAMK**	116	89	83	31	103	74
33	**CAMK2D**	**CAMK**	102	87	16	1	99	64
34	**CAMK2G**	**CAMK**	93	82	34	6	94	82

#	Kinase Name	Kinase Family*	M17 0.3 µM	M17 3 µM	M36 0.3 µM	M36 3 µM	M16 0.03 µM	M16 0.3 µM
35	**CAMK4**	**CAMK**	90	82	92	73	96	92
36	**CAMKK1**	**OTHER**	86	90	51	18	97	95
37	**CAMKK2**	**OTHER**	87	96	9	1	92	55
38	**CDC42BPA**	**AGC**	100	76	1	1	53	7
39	**CDC42BPB**	**AGC**	116	83	0	0	37	2
40	**CDC7/DBF4**	**OTHER**	105	72	10	2	63	12
41	**CDK1/CycA2**	**CMGC**	78	68	2	1	32	5
42	**CDK1/CycB1**	**CMGC**	98	78	4	1	29	5
43	**CDK1/CycE1**	**CMGC**	84	70	2	0	42	8
44	**CDK12/CycK**	**CMGC**	84	65	1	2	21	1
45	**CDK13/CycK**	**CMGC**	109	92	12	-10	31	5
46	**CDK16/CycY**	**CMGC**	101	88	17	3	64	16
47	**CDK17/p35NCK**	**CMGC**	86	64	20	10	46	24
48	**CDK18/CycY**	**CMGC**	97	81	6	0	36	5
49	**CDK19/CycC**	**CMGC**	41	18	3	0	6	2
50	**CDK2/CycA2**	**CMGC**	83	66	1	2	18	2
51	**CDK2/CycD1**	**CMGC**	82	92	6	5	54	23
52	**CDK2/CycE1**	**CMGC**	90	57	1	1	16	0
53	**CDK20/CycH**	**CMGC**	88	50	29	13	38	33
54	**CDK20/CycT1**	**CMGC**	84	55	19	10	36	17
55	**CDK3/CycC**	**CMGC**	98	85	9	3	71	20
56	**CDK3/CycE1**	**CMGC**	99	65	1	-1	36	5
57	**CDK4/CycD1**	**CMGC**	93	79	24	4	86	36
58	**CDK4/CycD2**	**CMGC**	90	85	21	3	81	34
59	**CDK4/CycD3**	**CMGC**	85	86	34	6	95	51
60	**CDK5/p25NCK**	**CMGC**	93	91	0	1	16	1
61	**CDK5/p35NCK**	**CMGC**	99	73	0	0	7	1
62	**CDK6/CycD1**	**CMGC**	94	86	2	1	93	51
63	**CDK6/CycD2**	**CMGC**	92	84	47	10	93	76
64	**CDK6/CycD3**	**CMGC**	80	84	76	26	88	98
65	**CDK7/CycH/MAT1**	**CMGC**	98	80	7	2	44	6
66	**CDK8/CycC**	**CMGC**	41	13	5	2	6	4
67	**CDK9/CycK**	**CMGC**	73	35	2	0	7	0
68	**CDK9/CycT1**	**CMGC**	87	52	1	0	13	2
69	**CHK1**	**CAMK**	87	68	76	40	96	87
70	**CHK2**	**CAMK**	97	91	81	41	90	82
71	**CK1α1**	**CK1**	136	122	110	51	129	93
72	**CK1Δ**	**CK1**	109	86	43	9	77	17
73	**CK1ε**	**CK1**	124	103	86	23	112	41
74	**CK1γ1**	**CK1**	107	96	23	3	87	38
75	**CK1γ2**	**CK1**	98	97	19	3	86	30
76	**CK1γ3**	**CK1**	105	95	49	9	77	22
77	**CK2α1**	**OTHER**	105	106	99	53	106	104
78	**CK2α2**	**OTHER**	105	99	91	43	110	105
79	**CLK1**	**CMGC**	73	35	4	2	11	3
80	**CLK2**	**CMGC**	102	45	1	2	8	1
81	**CLK3**	**CMGC**	130	108	13	1	94	36
82	**CLK4**	**CMGC**	56	11	1	1	3	0
83	**COT**	**STE**	90	103	85	92	104	86
84	**CSF1R**	**TK**	97	78	33	14	90	88
85	**CSK**	**TK**	106	103	116	71	114	106

#	Kinase Name	Kinase Family*	M17 0.3 µM	M17 3 µM	M36 0.3 µM	M36 3 µM	M16 0.03 µM	M16 0.3 µM
86	**DAPK1**	**CAMK**	154	127	119	64	112	72
87	**DAPK2**	**CAMK**	128	107	118	67	106	78
88	**DAPK3**	**CAMK**	110	91	80	25	98	51
89	**DCAMKL2**	**CAMK**	88	86	91	81	92	85
90	**DDR2**	**TK**	103	92	59	17	105	93
91	**DMPK**	**AGC**	104	92	3	-2	86	63
92	**DNAPK**	**ATYPICAL**	96	88	71	25	100	97
93	**DYRK1A**	**CMGC**	93	57	7	0	11	2
94	**DYRK1B**	**CMGC**	83	36	7	0	8	0
95	**DYRK2**	**CMGC**	93	56	31	4	6	1
96	**DYRK3**	**CMGC**	93	82	59	14	27	2
97	**DYRK4**	**CMGC**	95	82	66	22	39	7
98	**EEF2K**	**ATYPICAL**	86	90	84	97	96	89
99	**EGFR**	**TK**	118	101	107	53	109	90
100	**EIF2AK2**	**OTHER**	76	76	79	63	83	95
101	**EIF2AK3**	**OTHER**	84	82	81	74	87	90
102	**EPHA1**	**TK**	120	114	127	126	128	116
103	**EPHA2**	**TK**	103	92	101	72	109	101
104	**EPHA3**	**TK**	105	100	111	103	102	94
105	**EPHA4**	**TK**	111	80	93	72	105	85
106	**EPHA5**	**TK**	100	102	146	84	115	102
107	**EPHA6**	**TK**	130	85	101	65	98	105
108	**EPHA7**	**TK**	128	107	131	93	122	107
109	**EPHA8**	**TK**	111	96	113	94	119	107
110	**EPHB1**	**TK**	116	107	124	79	98	97
111	**EPHB2**	**TK**	92	89	85	47	97	95
112	**EPHB3**	**TK**	98	98	91	67	101	99
113	**EPHB4**	**TK**	110	98	110	85	97	90
114	**ERBB2**	**TK**	92	84	90	44	102	102
115	**ERBB4**	**TK**	111	90	101	56	108	90
116	**ERK1**	**CMGC**	94	96	72	37	111	96
117	**ERK2**	**CMGC**	104	94	79	30	103	87
118	**ERK5**	**CMGC**	84	43	13	8	28	22
119	**ERK7**	**CMGC**	98	63	7	-3	86	59
120	**FAK**	**TK**	112	96	85	60	102	89
121	**FER**	**TK**	81	81	66	22	63	58
122	**FES**	**TK**	95	72	83	50	93	87
123	**FGFR1**	**TK**	93	82	56	16	103	91
124	**FGFR2**	**TK**	91	84	52	9	94	85
125	**FGFR3**	**TK**	96	93	64	20	93	84
126	**FGFR4**	**TK**	106	100	98	58	91	87
127	**FGR**	**TK**	107	95	40	6	111	105
128	**FLT3**	**TK**	101	78	9	2	49	21
129	**FRK**	**TK**	100	99	54	16	103	99
130	**FYN**	**TK**	98	84	22	3	93	69
131	**GRK2**	**AGC**	102	88	99	51	96	106
132	**GRK3**	**AGC**	117	105	98	74	111	107
133	**GRK4**	**AGC**	118	109	108	95	101	90
134	**GRK5**	**AGC**	118	110	119	74	118	91
135	**GRK6**	**AGC**	104	96	96	54	98	93
136	**GRK7**	**AGC**	114	98	94	48	105	78

#	Kinase Name	Kinase Family*	M17 0.3 µM	M17 3 µM	M36 0.3 µM	M36 3 µM	M16 0.03 µM	M16 0.3 µM
137	**GSG2**	**OTHER**	35	5	2	0	2	0
138	**GSK3α**	**CMGC**	127	80	41	7	99	93
139	**GSK3β**	**CMGC**	81	62	18	2	80	66
140	**HCK**	**TK**	105	81	75	25	106	81
141	**HIPK1**	**CMGC**	113	90	47	10	49	10
142	**HIPK2**	**CMGC**	81	58	22	3	36	4
143	**HIPK3**	**CMGC**	95	82	45	11	53	7
144	**HIPK4**	**CMGC**	95	85	27	2	74	24
145	**HRI**	**OTHER**	106	89	93	71	98	90
146	**IGF1R**	**TK**	130	115	128	83	116	99
147	**IKKα**	**OTHER**	100	39	37	13	27	16
148	**IKKβ**	**OTHER**	96	60	80	36	85	64
149	**IKKε**	**OTHER**	99	90	40	6	94	61
150	**INSR**	**TK**	97	89	82	36	96	91
151	**INSRR**	**TK**	87	89	85	39	97	96
152	**IRAK1**	**TKL**	110	86	49	9	95	76
153	**IRAK4**	**TKL**	85	66	14	2	83	50
154	**ITK**	**TK**	97	93	77	16	105	88
155	**JAK1**	**TK**	88	77	58	25	78	86
156	**JAK2**	**TK**	98	83	40	8	102	80
157	**JAK3**	**TK**	87	66	5	-1	58	15
158	**JNK1**	**CMGC**	106	81	74	23	102	95
159	**JNK2**	**CMGC**	80	73	63	29	78	85
160	**JNK3**	**CMGC**	93	83	54	13	73	68
161	**KIT**	**TK**	96	82	89	46	70	96
162	**LCK**	**TK**	98	90	61	18	87	91
163	**LIMK1**	**TKL**	85	39	9	1	10	3
164	**LIMK2**	**TKL**	105	89	70	27	106	101
165	**LRRK2**	**TKL**	85	58	1	1	50	8
166	**LTK**	**TK**	100	88	46	11	110	98
167	**LYN**	**TK**	103	95	73	25	105	81
168	**MAP3K1**	**STE**	90	89	94	93	105	106
169	**MAP3K10**	**STE**	94	89	50	9	99	76
170	**MAP3K11**	**STE**	104	85	45	4	100	74
171	**MAP3K7/MAP3K7IP1**	**STE**	81	61	15	5	54	17
172	**MAP3K9**	**STE**	93	86	57	12	95	80
173	**MAP4K2**	**STE**	83	50	3	0	22	4
174	**MAP4K4**	**STE**	83	35	6	2	76	29
175	**MAP4K5**	**STE**	98	78	3	0	87	52
176	**MAPKAPK2**	**CAMK**	143	118	130	87	114	95
177	**MAPKAPK3**	**CAMK**	107	96	102	90	99	87
178	**MAPKAPK5**	**CAMK**	106	89	106	69	104	85
179	**MARK1**	**CAMK**	93	99	90	43	121	101
180	**MARK2**	**CAMK**	109	96	98	63	91	88
181	**MARK3**	**CAMK**	111	98	89	48	99	86
182	**MARK4**	**CAMK**	102	93	90	54	99	91
183	**MASTL**	**AGC**	85	84	64	26	87	56
184	**MATK**	**TK**	122	117	134	94	107	90
185	**MEK1**	**STE**	113	89	35	6	108	102
186	**MEK2**	**STE**	88	61	13	3	66	48
187	**MEK5**	**STE**	88	52	30	12	35	31

#	Kinase Name	Kinase Family*	M17 0.3 µM	M17 3 µM	M36 0.3 µM	M36 3 µM	M16 0.03 µM	M16 0.3 µM
188	**MEKK2**	**STE**	105	100	66	18	95	91
189	**MEKK3**	**STE**	104	97	66	26	106	90
190	**MELK**	**CAMK**	91	29	36	9	74	14
191	**MERTK**	**TK**	109	95	69	36	97	86
192	**MET**	**TK**	103	99	99	48	110	106
193	**MINK1**	**STE**	78	25	2	0	75	20
194	**MKK4**	**STE**	86	81	45	9	86	66
195	**MKK6 SDTD**	**STE**	77	75	63	30	77	87
196	**MKK7**	**STE**	94	83	81	48	96	88
197	**MKNK1**	**CAMK**	86	68	48	16	97	70
198	**MKNK2**	**CAMK**	91	65	16	3	93	62
199	**MLK4**	**TKL**	103	101	73	40	105	75
200	**MST1**	**STE**	97	87	4	1	88	49
201	**MST2**	**STE**	104	87	19	4	100	77
202	**MST3**	**STE**	88	87	71	32	101	91
203	**MST4**	**STE**	81	72	52	12	78	81
204	**MTOR**	**ATYPICAL**	104	87	78	24	91	105
205	**MUSK**	**TK**	83	73	28	5	88	70
206	**MYLK**	**CAMK**	107	66	62	25	103	71
207	**MYLK2**	**CAMK**	78	58	61	26	65	32
208	**MYLK3**	**CAMK**	116	68	71	21	119	77
209	**NEK1**	**OTHER**	82	79	84	55	86	67
210	**NEK11**	**OTHER**	92	85	105	85	97	87
211	**NEK2**	**OTHER**	103	94	95	72	103	96
212	**NEK3**	**OTHER**	101	87	94	79	96	78
213	**NEK4**	**OTHER**	104	90	84	37	89	47
214	**NEK6**	**OTHER**	100	109	112	108	107	104
215	**NEK7**	**OTHER**	111	104	103	107	111	96
216	**NEK9**	**OTHER**	86	86	88	67	89	87
217	**NIK**	**STE**	89	84	59	33	94	79
218	**NLK**	**CMGC**	101	87	78	29	96	81
219	**p38α**	**CMGC**	85	79	78	80	94	74
220	**p38β**	**CMGC**	81	76	77	84	79	88
221	**p38Δ**	**CMGC**	101	85	59	14	79	24
222	**p38γ**	**CMGC**	94	84	64	19	56	8
223	**PAK1**	**STE**	88	88	85	76	106	98
224	**PAK2**	**STE**	108	103	71	17	92	93
225	**PAK3**	**STE**	100	92	85	40	85	84
226	**PAK4**	**STE**	101	86	56	16	86	74
227	**PAK6**	**STE**	94	83	54	14	88	76
228	**PAK7**	**STE**	94	77	42	9	89	81
229	**PASK**	**CAMK**	100	47	3	0	82	39
230	**PBK**	**OTHER**	110	67	69	80	95	90
231	**PDGFRα**	**TK**	85	73	25	8	89	60
232	**PDGFRβ**	**TK**	97	81	24	4	93	75
233	**PDK1**	**AGC**	91	75	160	39	98	83
234	**PHKG1**	**CAMK**	105	88	16	2	85	39
235	**PHKG2**	**CAMK**	66	62	52	23	74	52
236	**PIM1**	**CAMK**	84	57	55	14	77	40
237	**PIM2**	**CAMK**	108	98	119	90	115	83
238	**PIM3**	**CAMK**	89	54	85	46	118	68

#	Kinase Name	Kinase Family*	M17 0.3 µM	M17 3 µM	M36 0.3 µM	M36 3 µM	M16 0.03 µM	M16 0.3 µM
239	**PKA**	**AGC**	85	81	12	2	75	43
240	**PKCα**	**AGC**	105	82	49	11	95	80
241	**PKCβ1**	**AGC**	110	92	53	9	103	50
242	**PKCβ2**	**AGC**	106	85	51	11	106	71
243	**PKCΔ**	**AGC**	109	72	4	0	47	6
244	**PKCε**	**AGC**	81	77	5	1	25	4
245	**PKCη**	**AGC**	93	56	2	2	8	2
246	**PKCγ**	**AGC**	100	77	33	5	99	54
247	**PKCι**	**AGC**	91	86	75	32	105	94
248	**PKCµ**	**AGC**	93	74	63	23	77	24
249	**PKCν**	**AGC**	78	65	62	17	89	32
250	**PKCΘ**	**AGC**	97	70	2	1	40	7
251	**PKCζ**	**AGC**	115	106	85	29	97	78
252	**PKMYT1**	**OTHER**	79	53	39	13	32	23
253	**PKMzeta**	**AGC**	102	89	89	68	97	90
254	**PKN3**	**AGC**	84	84	27	3	83	74
255	**PLK1**	**OTHER**	96	92	100	66	95	90
256	**PLK3**	**OTHER**	111	94	97	87	95	92
257	**PRK1**	**AGC**	94	87	17	3	84	35
258	**PRK2**	**AGC**	78	69	8	0	90	42
259	**PRKD2**	**CAMK**	91	80	68	25	87	37
260	**PRKG1**	**AGC**	84	53	6	1	75	30
261	**PRKG2**	**AGC**	77	20	14	2	48	7
262	**PRKX**	**AGC**	123	85	15	-1	54	3
263	**PYK2**	**TK**	93	98	73	24	117	102
264	**RAF1 YDYD**	**TKL**	75	82	67	67	77	96
265	**RET**	**TK**	120	96	61	12	106	100
266	**RIPK2**	**TKL**	75	64	61	33	95	78
267	**RIPK4**	**TKL**	92	86	88	45	94	75
268	**RIPK5**	**TKL**	90	76	58	16	101	86
269	**ROCK1**	**AGC**	85	39	1	1	24	2
270	**ROCK2**	**AGC**	77	39	1	0	12	1
271	**RON**	**TK**	106	91	98	76	92	90
272	**ROS**	**TK**	93	72	26	5	92	81
273	**RPS6KA1**	**AGC**	97	54	20	10	46	22
274	**RPS6KA2**	**AGC**	90	63	6	0	87	34
275	**RPS6KA3**	**AGC**	86	56	7	1	85	39
276	**RPS6KA4**	**AGC**	91	88	71	29	90	89
277	**RPS6KA5**	**AGC**	87	73	46	15	89	82
278	**RPS6KA6**	**AGC**	78	40	13	3	84	48
279	**S6K**	**AGC**	99	73	82	37	99	84
280	**S6Kbeta**	**AGC**	117	100	87	67	89	103
281	**SAK**	**OTHER**	67	62	53	24	97	84
282	**SGK1**	**AGC**	75	41	30	10	92	76
283	**SGK2**	**AGC**	99	78	88	32	97	74
284	**SGK3**	**AGC**	91	82	81	27	94	84
285	**SIK1**	**CAMK**	89	81	87	48	102	98
286	**SIK2**	**CAMK**	103	81	86	27	96	102
287	**SIK3**	**CAMK**	94	83	72	32	106	88
288	**SLK**	**STE**	74	83	4	1	83	77
289	**SNARK**	**CAMK**	93	79	30	5	99	64

#	Kinase Name	Kinase Family*	M17 0.3 µM	M17 3 µM	M36 0.3 µM	M36 3 µM	M16 0.03 µM	M16 0.3 µM
290	**SNK**	**OTHER**	97	95	94	43	96	98
291	**SRC**	**TK**	104	91	88	24	95	86
292	**SRMS**	**TK**	104	91	93	73	101	93
293	**SRPK1**	**CMGC**	109	110	110	70	108	99
294	**SRPK2**	**CMGC**	99	98	87	55	93	91
295	**STK17A**	**CAMK**	87	36	23	4	83	23
296	**STK23**	**CAMK**	94	96	95	88	109	78
297	**STK25**	**STE**	99	91	33	6	99	81
298	**STK33**	**CAMK**	90	76	41	14	73	43
299	**STK39**	**STE**	98	77	40	19	77	66
300	**SYK**	**TK**	98	97	90	64	93	84
301	**TAOK2**	**STE**	125	102	60	19	107	89
302	**TAOK3**	**STE**	100	100	44	6	111	91
303	**TBK1**	**OTHER**	111	90	48	10	114	99
304	**TEC**	**TK**	114	101	114	96	110	101
305	**TGFBR1**	**TKL**	68	58	18	0	65	67
306	**TGFBR2**	**TKL**	67	67	72	24	109	85
307	**TIE2**	**TK**	102	108	113	65	132	105
308	**TLK1**	**AGC**	121	105	119	106	98	89
309	**TLK2**	**AGC**	152	125	149	88	111	91
310	**TNK1**	**TK**	93	86	29	9	82	39
311	**TRKA**	**TK**	101	81	9	1	83	40
312	**TRKB**	**TK**	98	72	10	2	68	29
313	**TRKC**	**TK**	103	91	18	2	72	36
314	**TSF1**	**OTHER**	85	52	57	8	109	104
315	**TSK2**	**CAMK**	102	93	103	94	107	92
316	**TSSK1**	**CAMK**	117	90	50	15	109	83
317	**TTBK1**	**CK1**	104	110	101	77	116	108
318	**TTBK2**	**CK1**	100	94	93	56	96	91
319	**TTK**	**OTHER**	109	85	55	17	49	8
320	**TXK**	**TK**	97	95	84	35	100	90
321	**TYK2**	**TK**	108	84	6	1	105	68
322	**TYRO3**	**TK**	85	87	94	64	104	89
323	**ULK2**	**OTHER**	90	83	82	45	96	54
324	**VEGFR1**	**TK**	98	83	83	43	110	89
325	**VEGFR2**	**TK**	102	82	60	19	102	98
326	**VEGFR3**	**TK**	100	84	64	23	90	77
327	**VRK1**	**CK1**	94	96	104	61	114	108
328	**VRK2**	**CK1**	115	106	93	45	103	92
329	**WEE1**	**OTHER**	98	419	72	37	80	99
330	**WNK1**	**OTHER**	106	97	107	98	101	100
331	**WNK2**	**OTHER**	89	71	86	74	99	86
332	**WNK3**	**OTHER**	106	93	106	104	85	85
333	**YES**	**TK**	108	96	65	19	100	90
334	**ZAK**	**TKL**	91	91	57	14	96	83
335	**ZAP70**	**TK**	101	89	110	85	95	83

Suppl. Table 2: Overview of CDK inhibitors of which some are commercially available and in use in pre-clinical trials, clinical phases or are applied in clinical treatment. Shown are the inhibited CDKs, their enzymatic concentrations, clinical trials and targeted cancer entities. All enzymatic IC_{50} values of all CDK inhibitors except meriolin 3, were taken from the manufacturer homepage of Selleckchem.com. The enzymatic IC_{50} values for meriolin 3 were taken from [10, 12]. All clinical trials were taken from the homepage of clinicaltrials.gov.

CDK inhibitor	inhibited CDKs	Enzymatic IC_{50} values	Clinical phase	Clinical trials
**Roscovitine (Seliciclib)**	CDK2<br>CDK5<br>Cdc2	0.7 µM<br>0.2 µM<br>0.65 µM	II	Cystic fibrosis (NCT02649751),<br>Cushing disease (NCT03774446, NCT02160730),<br>Adv. solid tumors (NCT00999401),<br>Breast Cancer (NCT01333423),<br>Non-small cell lung cancer (NCT00372073),<br>Colitis, ulcerative (NCT03844932) (MedChemExpress, clinicaltrials.gov)
**Flavopiridol (Alvocidib)**	CDK1<br>CDK2<br>CDK4<br>CDK6<br>CDK9	30 nM<br>40 nM<br>20-40 nM<br>60 nM<br>20 nM	II	67 NCT registered trials, which of relevance here:<br>Lymphoma (NCT00445341; NCT00005074; NCT00003039),<br>AML (NCT03969420; NCT03441555; NCT03298984),<br>B cell chronic lymphocytic leukemia (NCT0003620; NCT00098371; NCT00058240; NCT00064285), adult acute basophilic leukemia (NCT00101231), lymphocytic chronic leukemia (NCT00464633)
**R547**	CDK1<br>CDK2<br>CDK4	2 nM<br>3 nM<br>1 nM	I	Advanced Solid Tumors (NCT00400296)
**Meriolin 3**	CDK1<br>CDK2<br>CDK5<br>CDK9	170 nM<br>11 nM<br>170 nM<br>6 nM	pre-clinical	SH-SY5Y (neuroblastoma), HEK293 (embryo kidney), GBM (glioma), KMS-11 (myeloma), LS 174T (colorectal adenocarcinoma), HCT116 (colon carcinoma) [12]; additionally, in HCT 116 spheroids, Huh7 (hepatoma), F1 (hepatoma), human foreskin fibroblasts, Ewing's sarcoma xenografts (inoculation w. A4573) [10]; SW1088 (human

				anaplastic astrocytoma), U87 (human glioblastoma), native neural cells (astrocytes, neurons, astrocyte-neuron-co-culture) [14]
**Zotiraciclib (TG02)**	CDK1<br>CDK2<br>CDK9	9 nM<br>5 nM<br>3 nM	II	Brain tumor, cancer (NCT05588141); Brain tumor, astrocytoma, astroglioma (NCT02942264); chronic lymphocytic leukemia (NCT01699152); AML, ALL (NCT01204164); high-grade gliomas (NCT03904628);
**Dinaciclib (SCH 727965)**	CDK1<br>CDK2<br>CDK5<br>CDK9	3 nM<br>1 nM<br>1 nM<br>4 nM	III	18 NCT registered trials, which of relevance here:<br>AML (NCT03484520); rrCLL, rrMM, rrDLBCL (NCT02684617); CLL (NCT01650727; NCT01580228; NCT01515176)
**SNS-032 (BMS-387032)**	CDK2<br>CDK5<br>CDK7<br>CDK9	38 nM<br>340 nM<br>62 nM<br>4 nM	I	B-lymphoid malignancies, CLL, mCL (NCT00446342); tumors (NCT00292864)

Suppl. Table 3: Predicted ADME properties for meriolin 16 and 36.

	**Meriolin 16**	**Meriolin 36**
Consensus Log $P_{o/w}$*	1.32	3.38
Log *S* (ESOL)**	-2.59	-4.43
GI absorption	High	High
PAINS alerts	None	None
Lipinski's rule of 5	No violation	No violation

* Average value of 6 different predictions

** Method developed by Delaney JS, 2004 [15]

Supplemental References

1. Sanidas I, Morris R, Fella KA, Rumde PH, Boukhali M, Tai EC, et al. A Code of Mono-phosphorylation Modulates the Function of RB. Mol Cell. 2019;73:985-1000 e6.
2. Matthews HK, Bertoli C, de Bruin RAM. Cell cycle control in cancer. Nat Rev Mol Cell Biol. 2021;23:74-88.
3. Kõivomägi M, Swaffer MP, Turner JJ, Marinov G, Skotheim JM. Localized phosphorylation of RNA Polymerase II by G1 cyclin-Cdk promotes cell cycle entry. Science. 20212021.03.25.436872.
4. Aleem E, Arceci RJ. Targeting cell cycle regulators in hematologic malignancies. Front Cell Dev Biol. 2015;3:
5. Ding L, Cao J, Lin W, Chen H, Xiong X, Ao H, et al. The Roles of Cyclin-Dependent Kinases in Cell-Cycle Progression and Therapeutic Strategies in Human Breast Cancer. Int J Mol Sci. 2020;21:1960.
6. Hochegger H, Takeda S, Hunt T. Cyclin-dependent kinases and cell-cycle transitions: Does one fit all? Nat Rev Mol Cell Biol. 2008;9:910-6.
7. Bury M, Le Calvé B, Ferbeyre G, Blank V, Lessard F. New Insights into CDK Regulators: Novel Opportunities for Cancer Therapy. Trends Cell Biol. 2021;31:331-44.
8. Ma H, Seebacher NA, Hornicek FJ, Duan Z. Cyclin-dependent kinase 9 (CDK9) is a novel prognostic marker and therapeutic target in osteosarcoma. EBioMedicine. 2019;39:182-93.
9. Kolupaeva V, Janssens V. PP1 and PP2A phosphatases – cooperating partners in modulating retinoblastoma protein activation. FEBS J. 2013;280:627-43.
10. Bettayeb K, Tirado OM, Marionneau-Lambot S, Ferandin Y, Lozach O, Morris JC, et al. Meriolins, a new class of cell death inducing kinase inhibitors with enhanced selectivity for cyclin-dependent kinases. Cancer Res. 2007;67:8325-34.
11. Drießen D, Stuhldreier F, Frank A, Stark H, Wesselborg S, Stork B, et al. Novel meriolin derivatives as rapid apoptosis inducers. Bioorg Med Chem. 2019;27:3463-8.
12. Echalier A, Bettayeb K, Ferandin Y, Lozach O, Clement M, Valette A, et al. Meriolins (3-(pyrimidin-4-yl)-7-azaindoles): Synthesis, kinase inhibitory activity, cellular effects, and structure of a CDK2/cyclin A/meriolin complex. J Med Chem. 2008;51:737-51.
13. Manning G, Whyte DB, Martinez R, Hunter T, Sudarsanam S. The protein kinase complement of the human genome. Science. 2002;298:1912-34.
14. Jarry M, Lecointre C, Malleval C, Desrues L, Schouft MT, Lejoncour V, et al. Impact of meriolins, a new class of cyclin-dependent kinase inhibitors, on malignant glioma proliferation and neo-angiogenesis. Neuro Oncol. 2014;16:1484-98.
15. Delaney JS. ESOL: estimating aqueous solubility directly from molecular structure. J Chem Inf Comput Sci. 2004;44:1000-5.
