# Supplementary material for: Novel meriolin derivatives potently inhibit cell cycle progression and transcription in leukemia and lymphoma cells via inhibition of cyclin-dependent kinases (CDKs)
Source: Cell Death Discov. 2024 Jun 11;10:279. doi: 10.1038/s41420-024-02056-6 (PMC11167047; doi:10.1038/s41420-024-02056-6)

Original Western Blots

(all western blots were scanned via LI-COR Odyssey® imaging system)

Figure 4B (left panel)

4 h

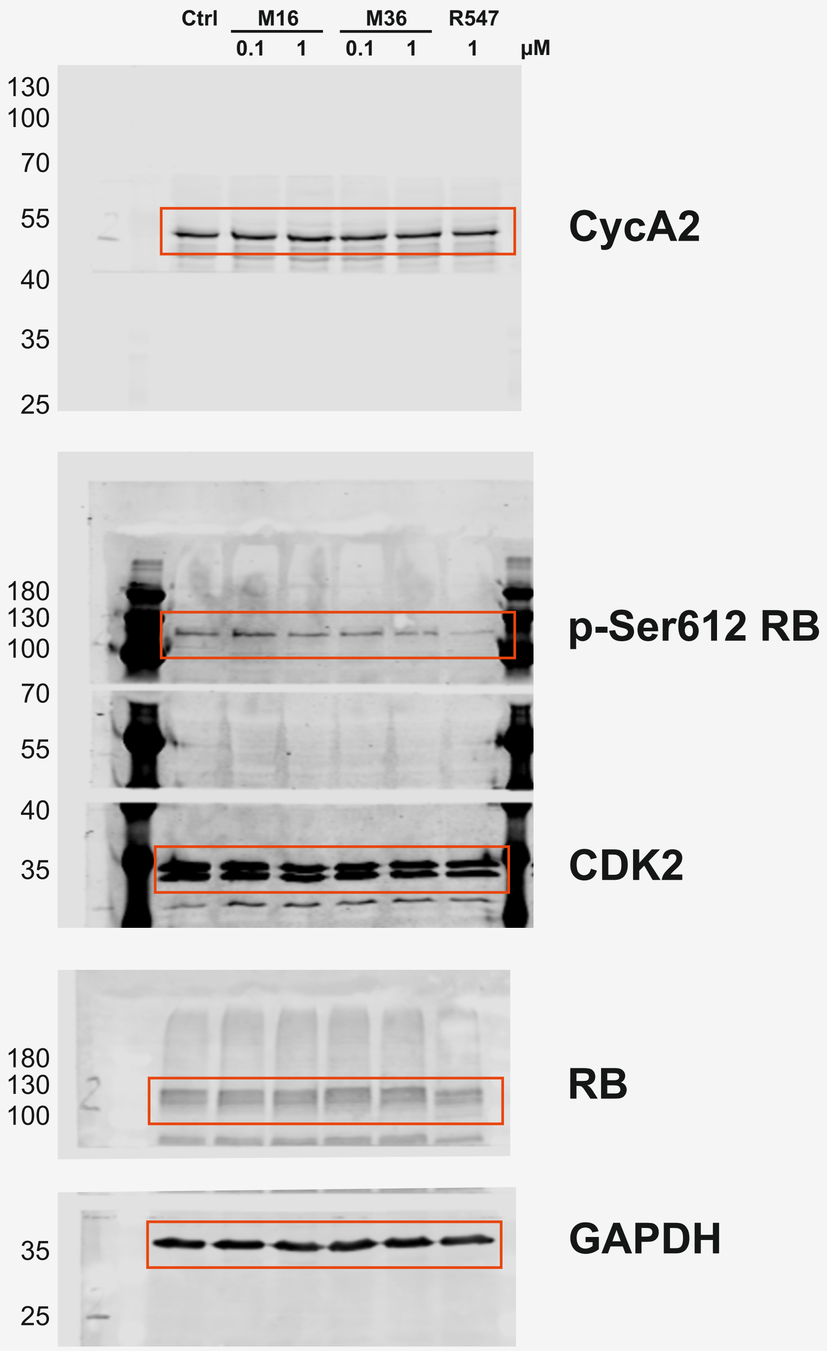

Figure 4B (left panel)

24 h

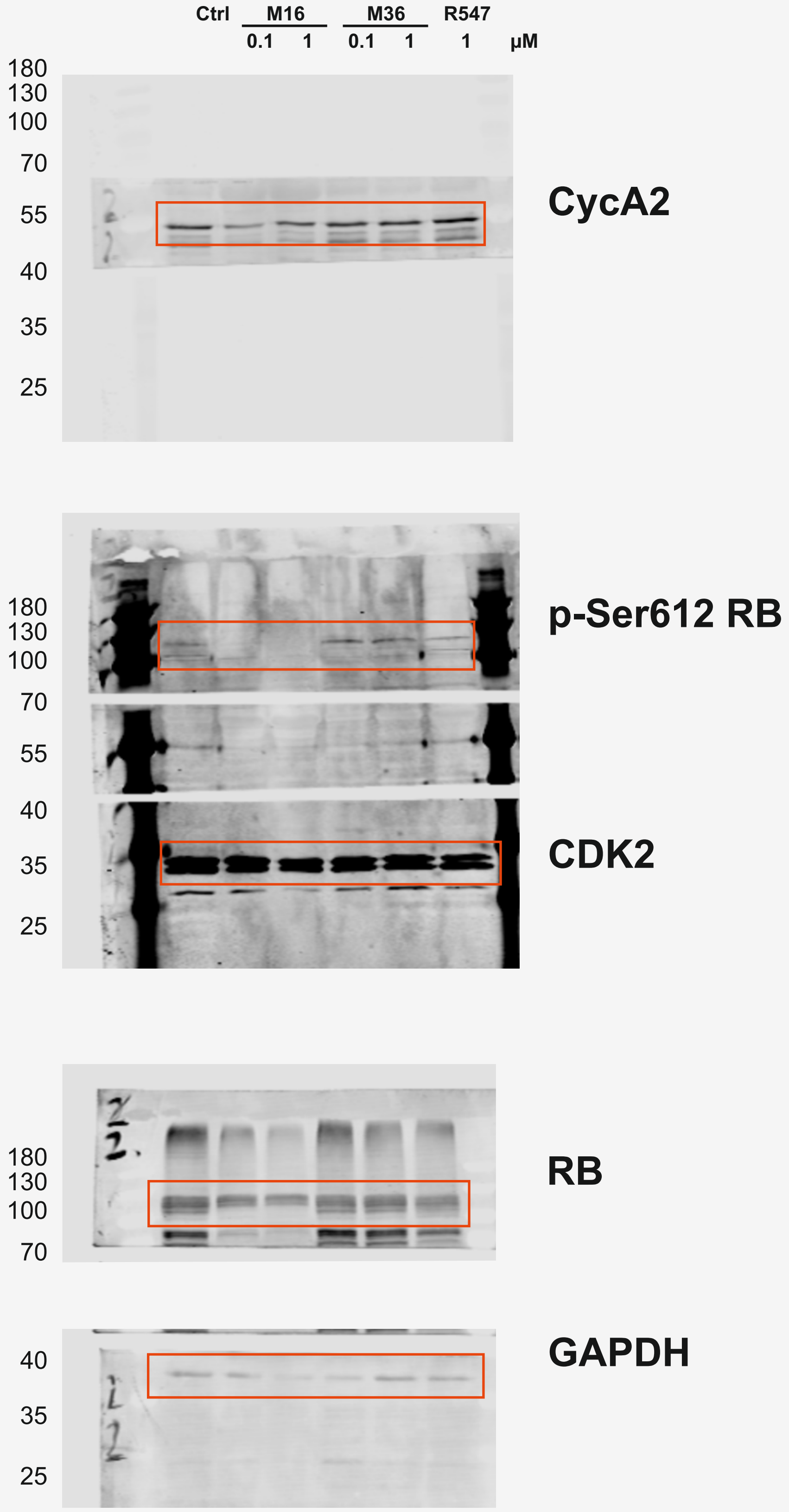

Figure 4B (right panel)

4 h

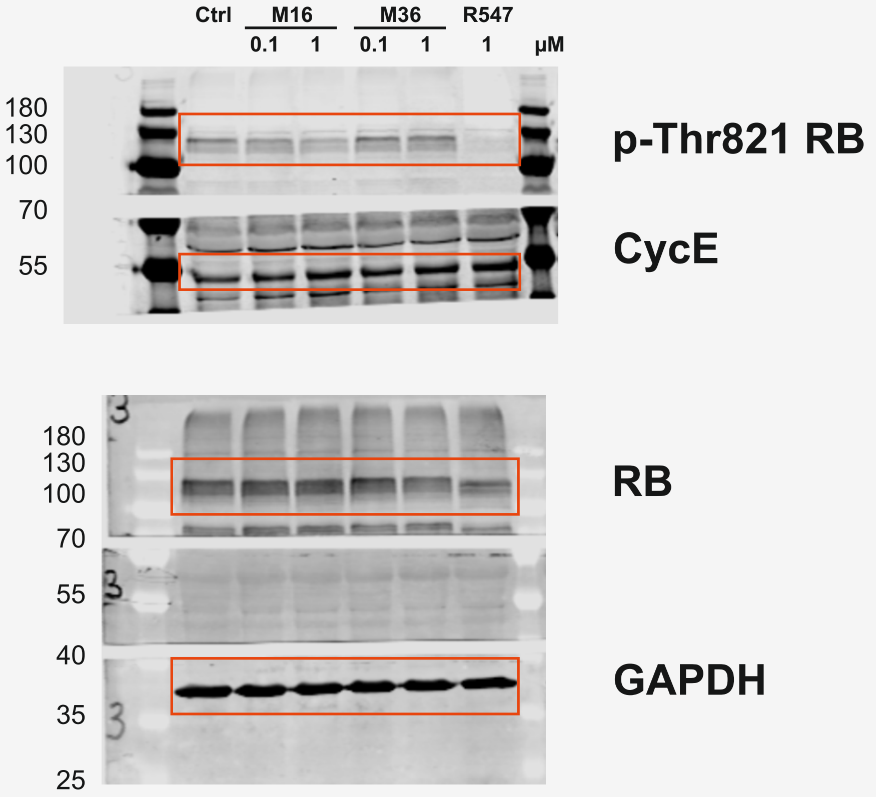

24 h

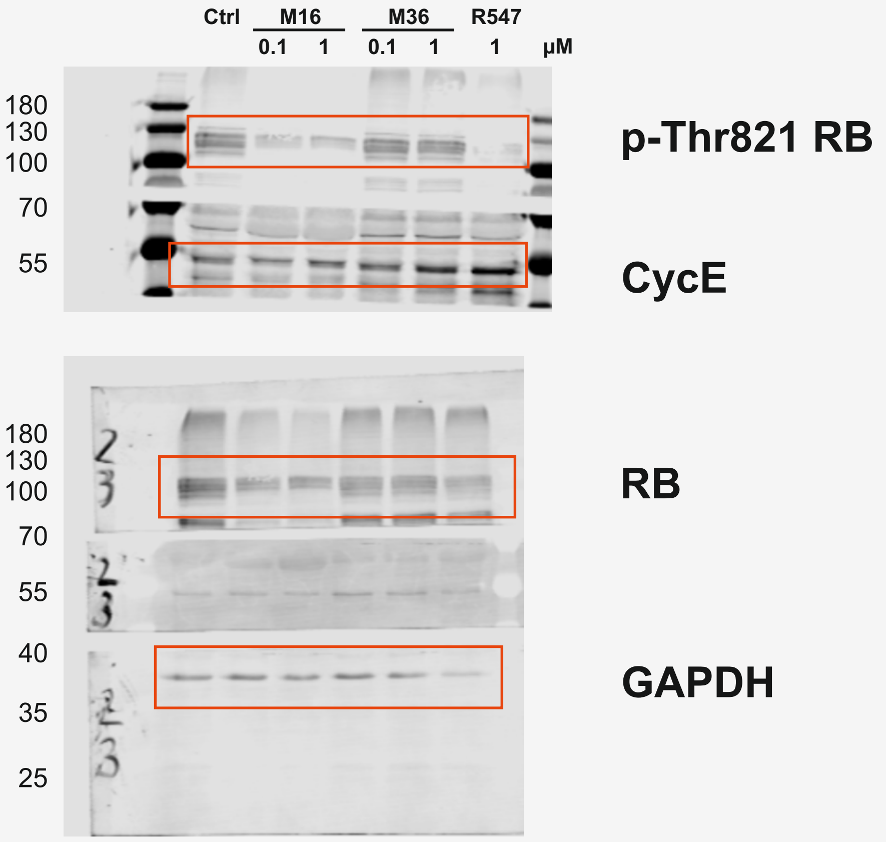

Figure 6B

4 h

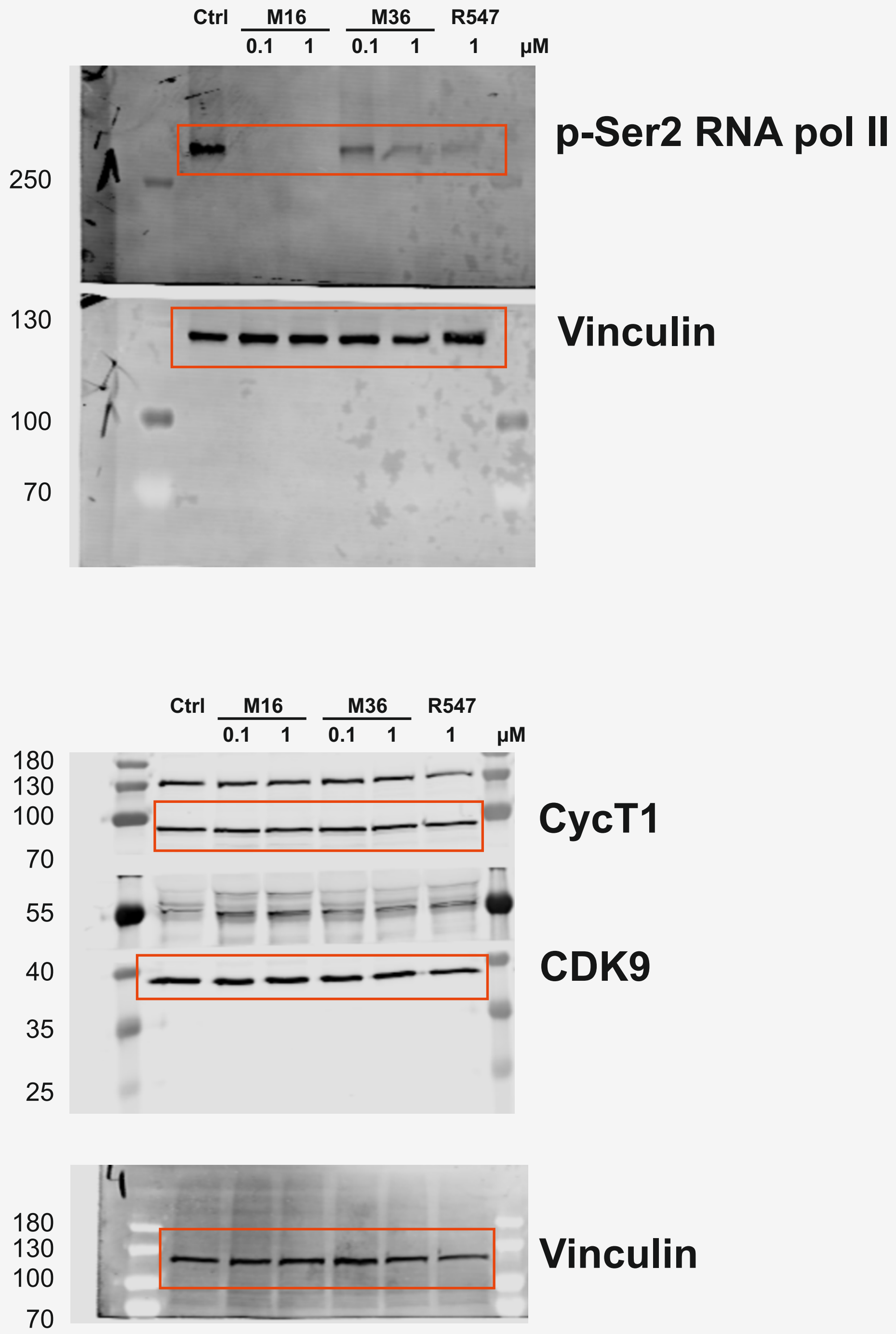

Ctrl
M16
M36
R547
0.1
1
0.1
1
1
µM
250
p-Ser2 RNA pol II
130
100
70
Vinculin
Ctrl
M16
M36
R547
0.1
1
0.1
1
1
µM
180
130
100
70
55
40
35
25
CycT1
CDK9
180
130
100
70
Vinculin

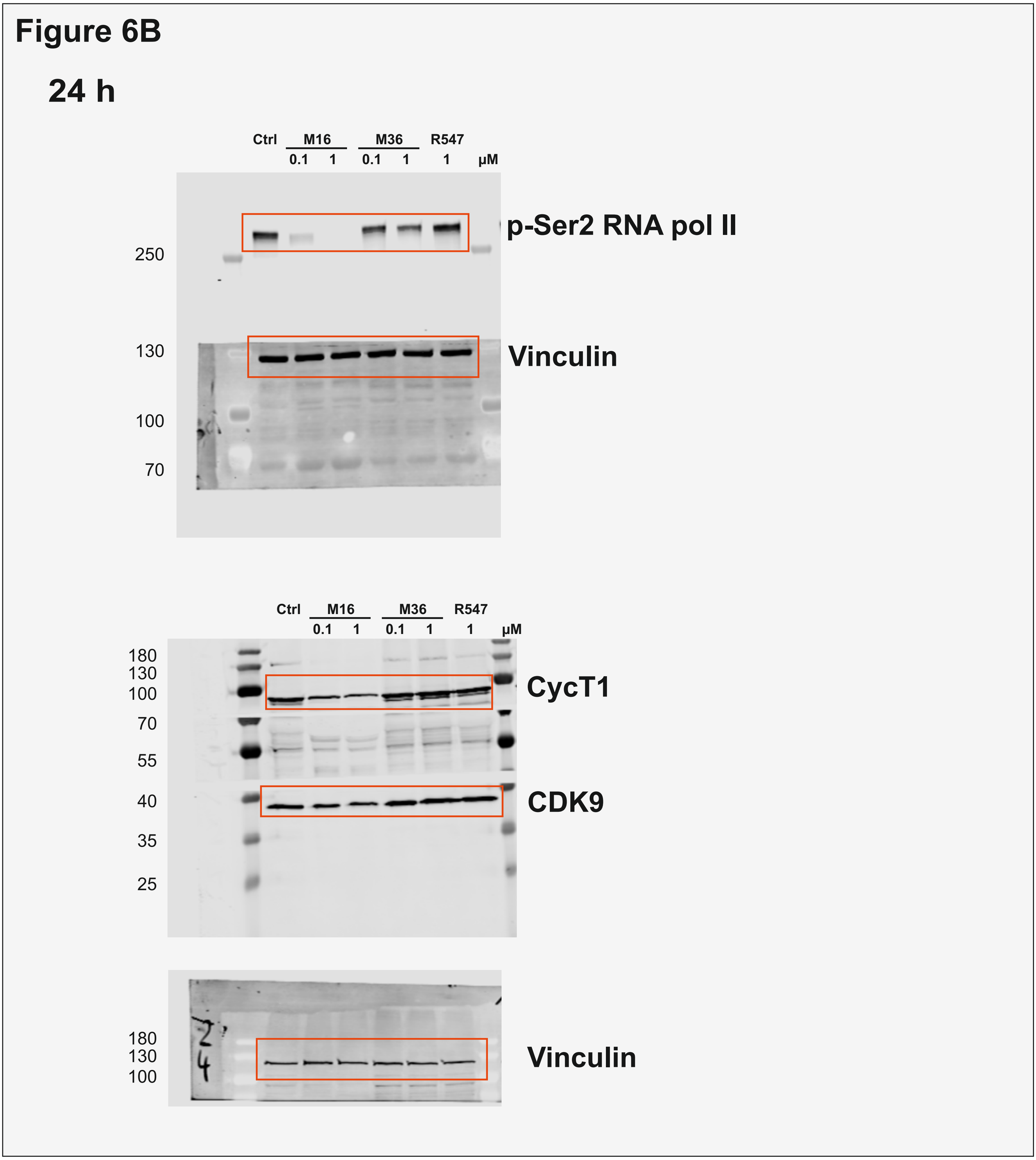

Figure 6B
24 h
Ctrl
M16
M36
R547
0.1
1
0.1
1
1
µM
250
130
100
70
p-Ser2 RNA pol II
Vinculin
Ctrl
M16
M36
R547
0.1
1
0.1
1
1
µM
180
130
100
70
55
40
35
25
CycT1
CDK9
180
130
100
Vinculin

Suppl. Figure 6A

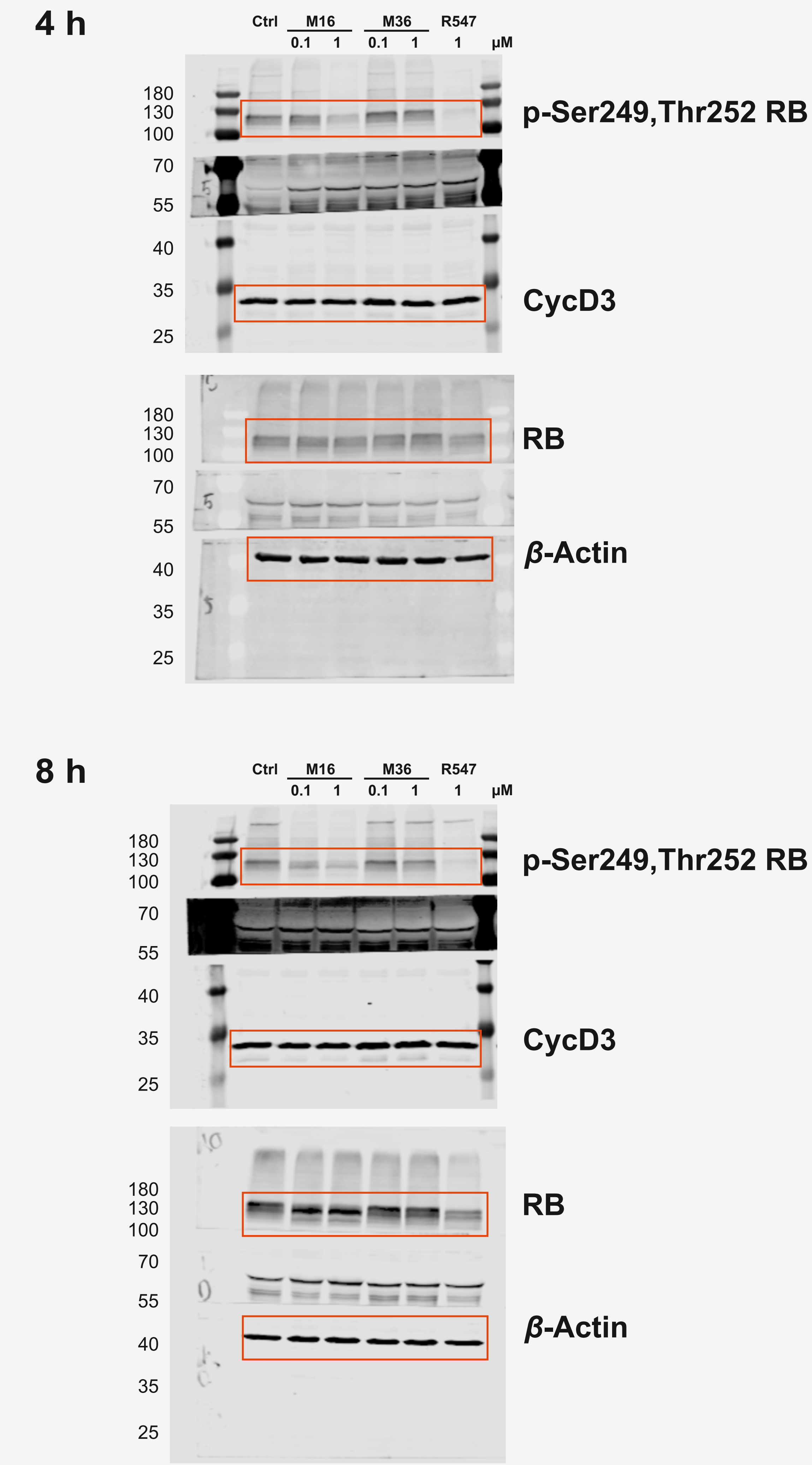

Suppl. Figure 6A

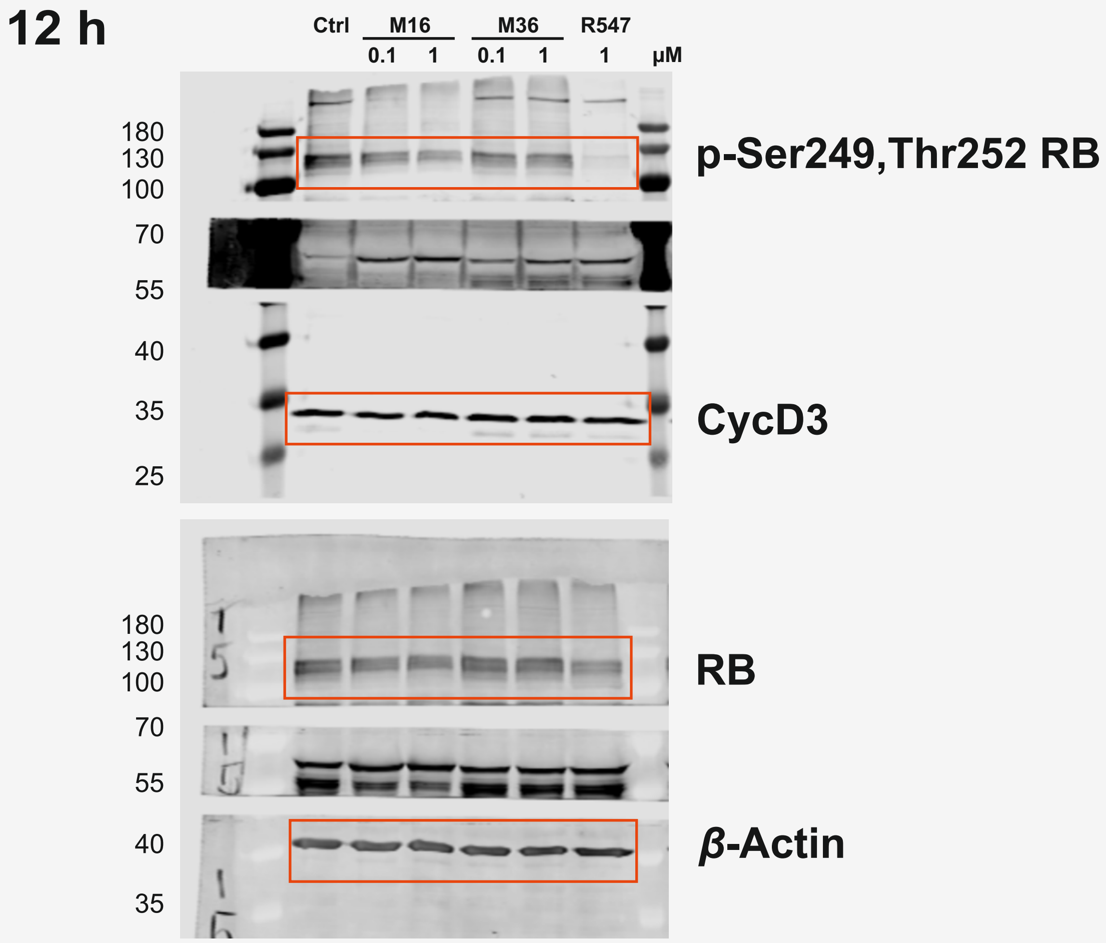

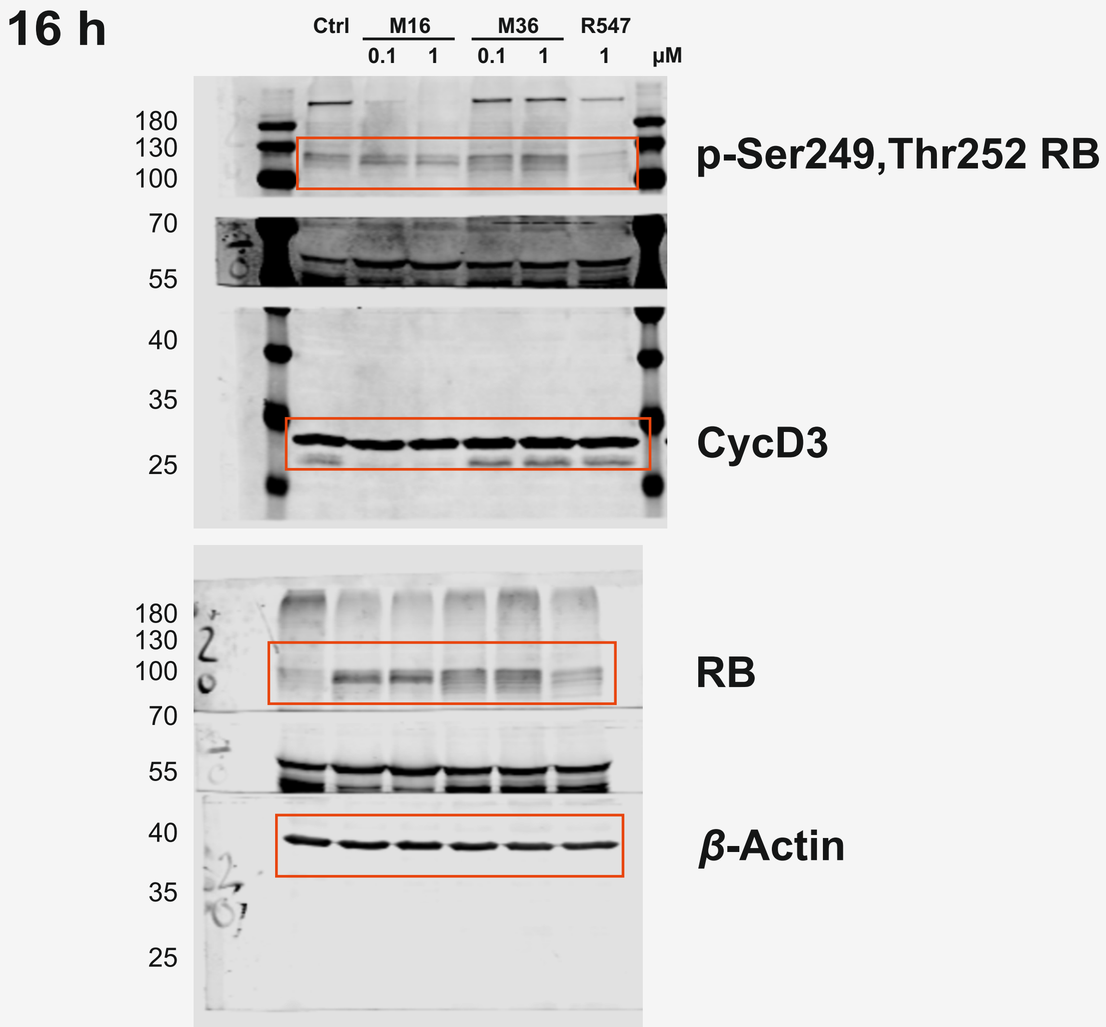

Suppl. Figure 6A

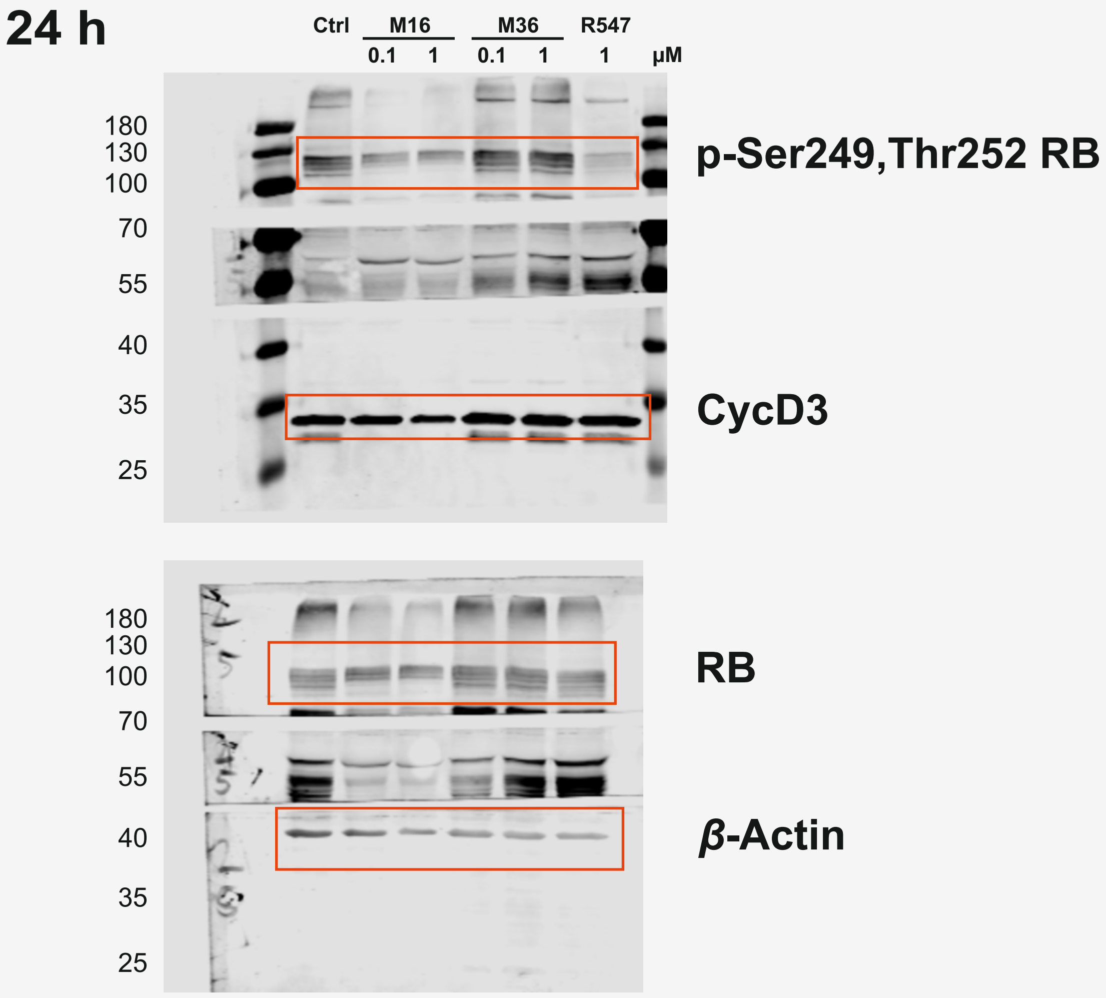

Suppl. Figure 6C

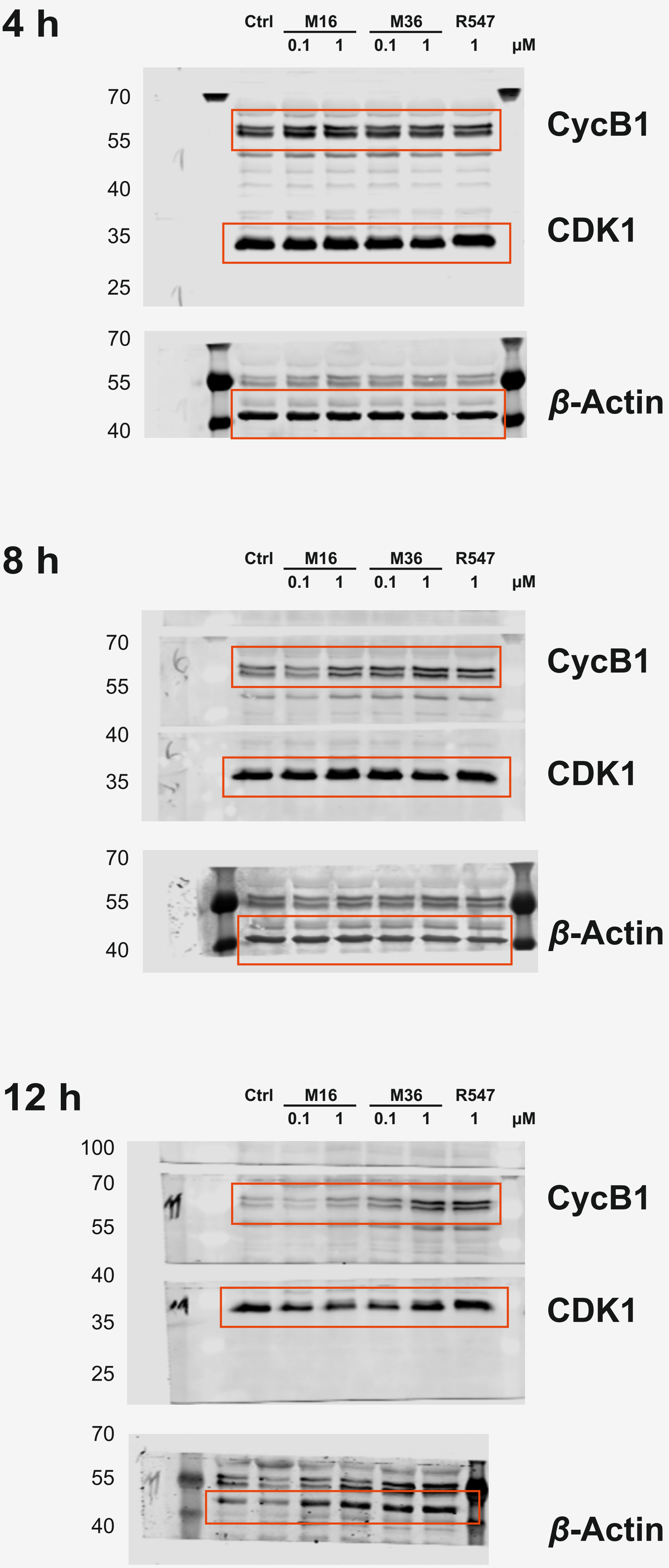

Suppl. Figure 6C

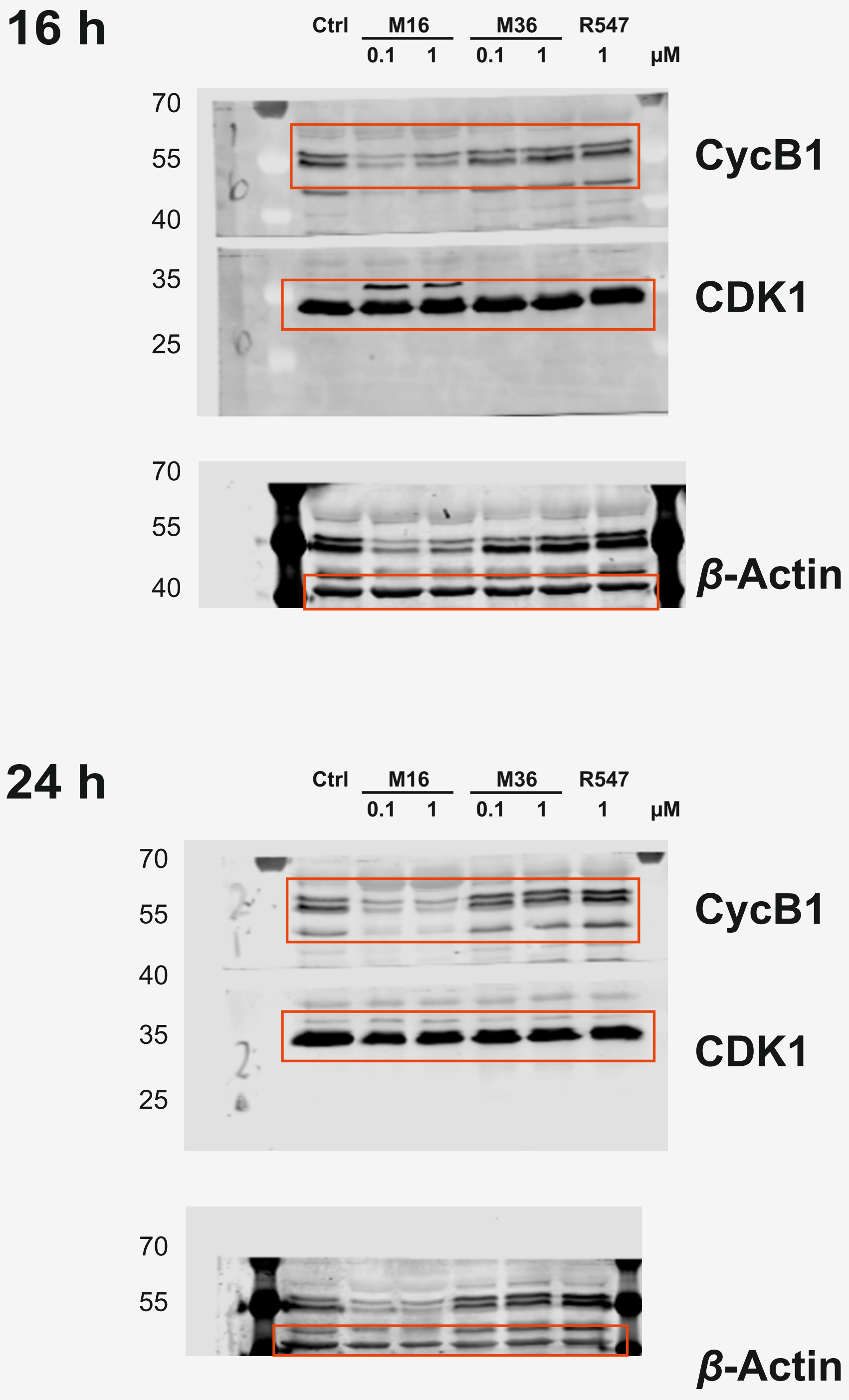

Suppl. Figure 7A

8 h

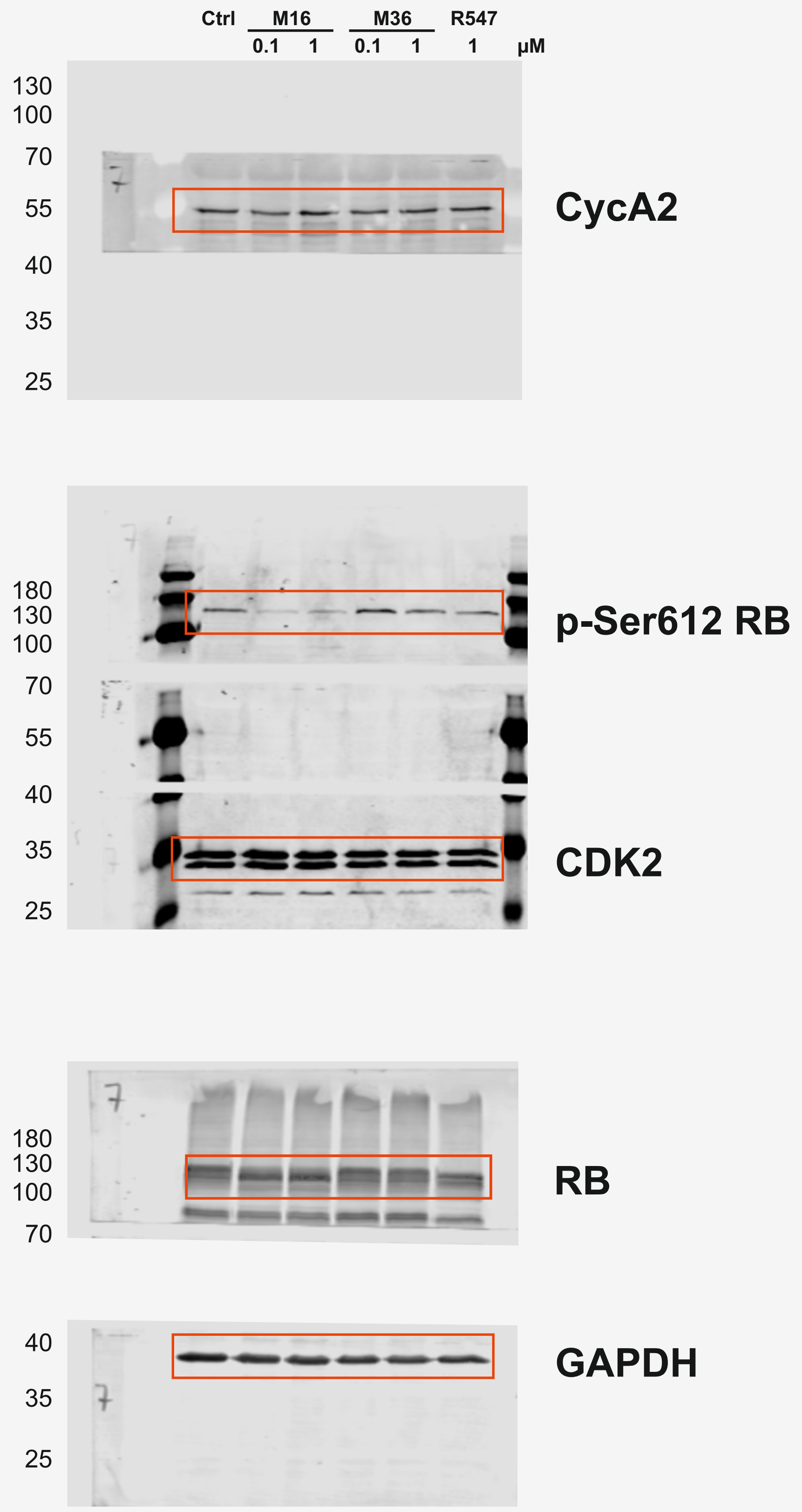

Suppl. Figure 7A

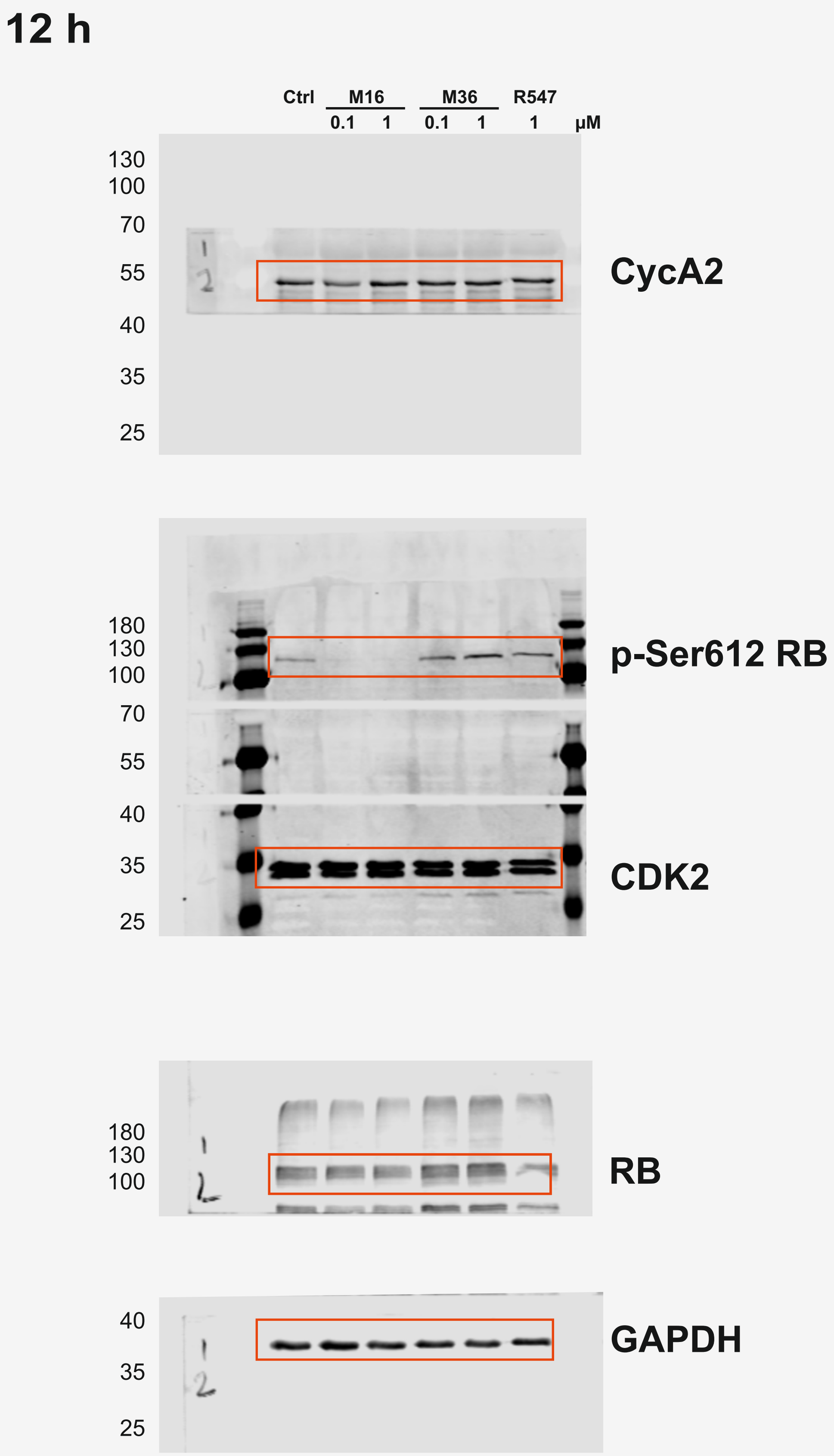

Suppl. Figure 7A

16 h

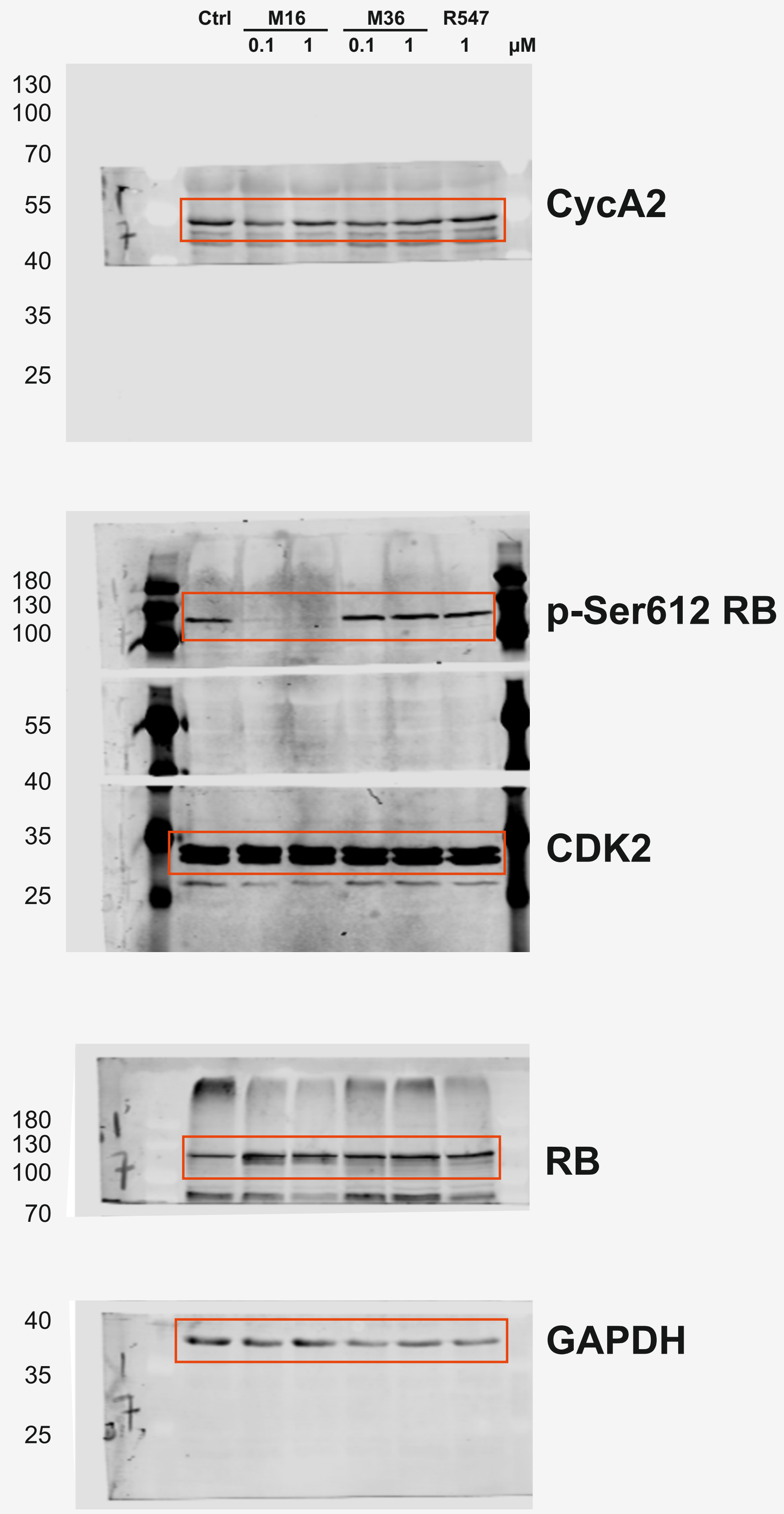

Suppl. Figure 7B

8 h

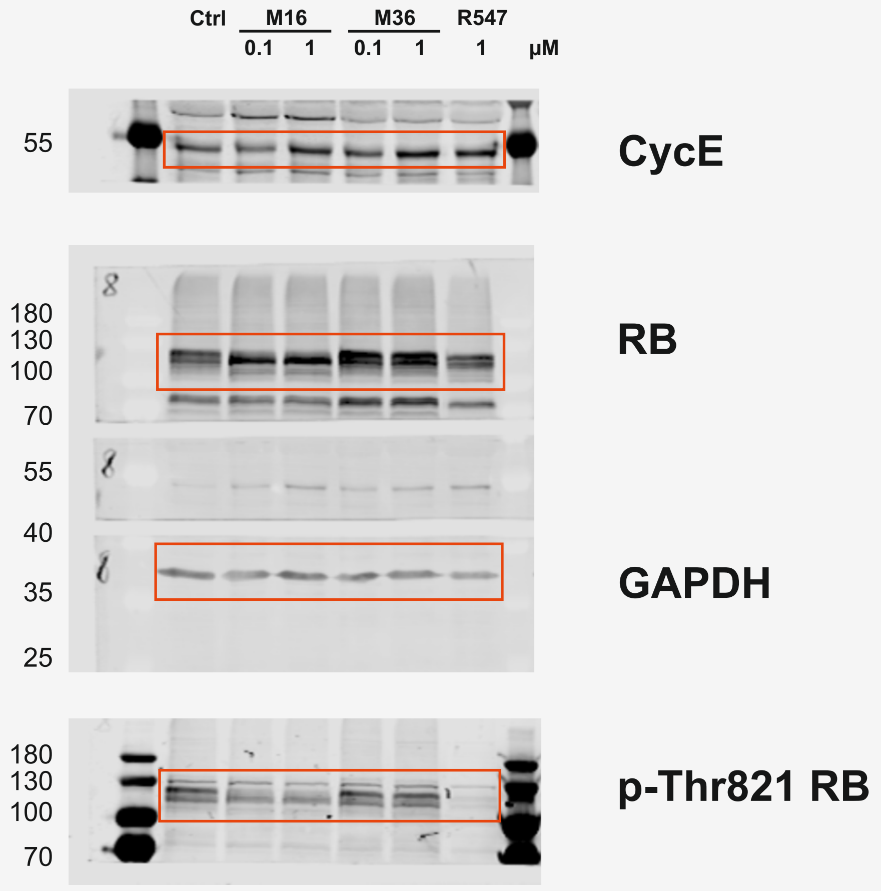

Suppl. Figure 7B

12 h

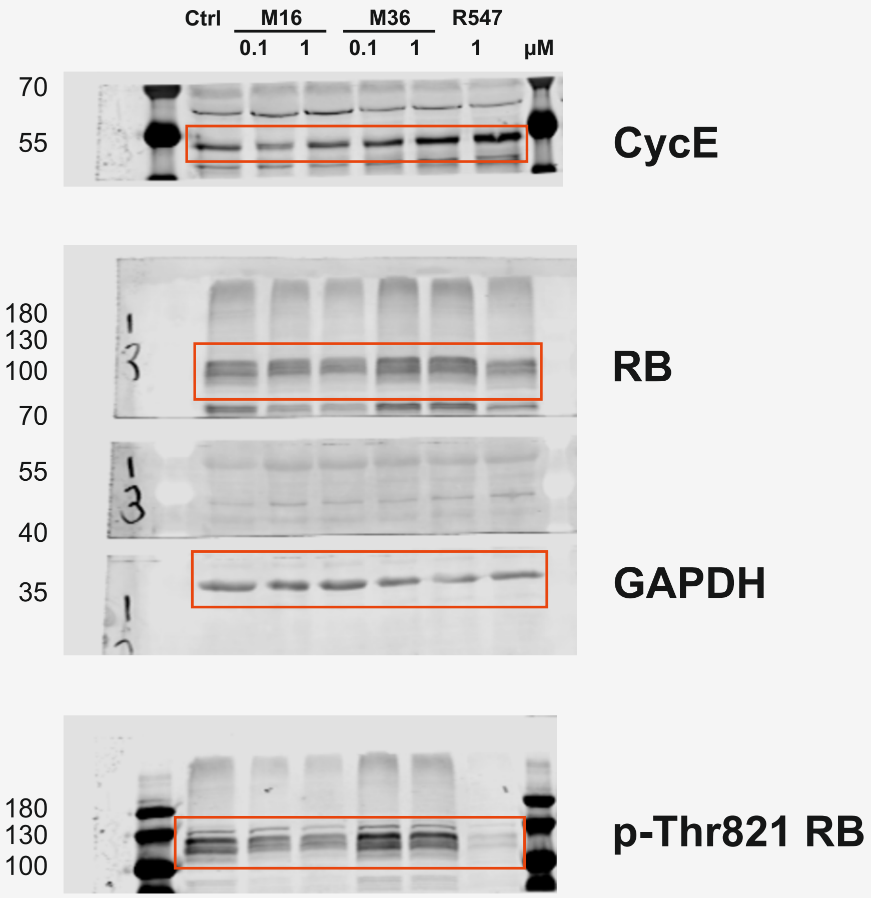

Suppl. Figure 7B

16 h

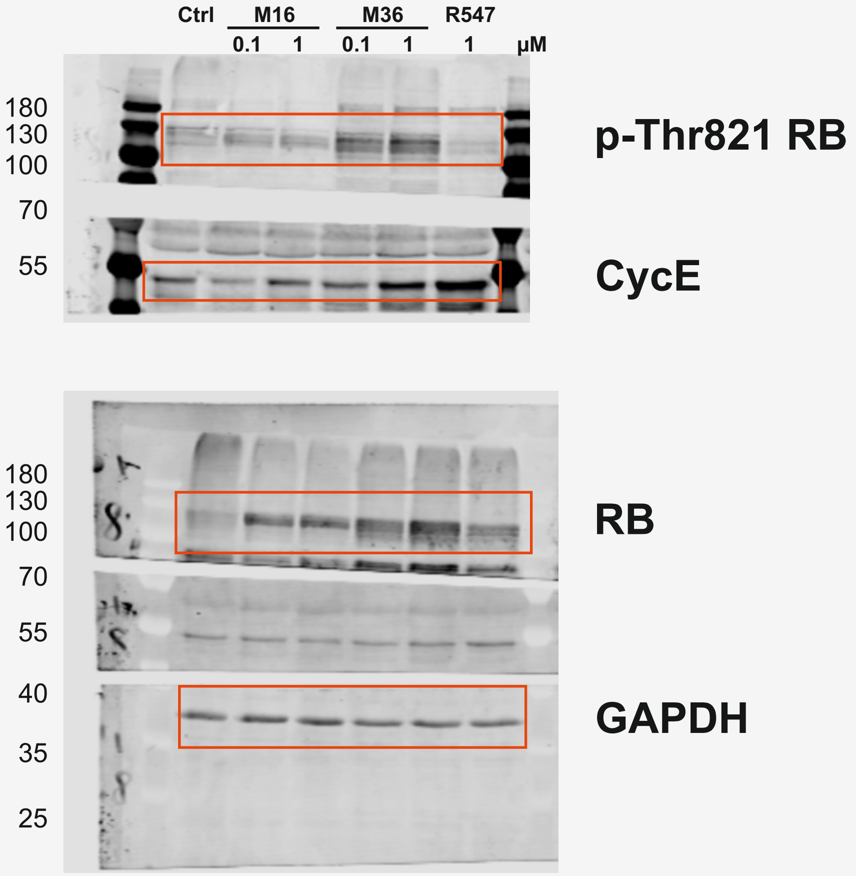

Suppl. Figure 7C

8 h

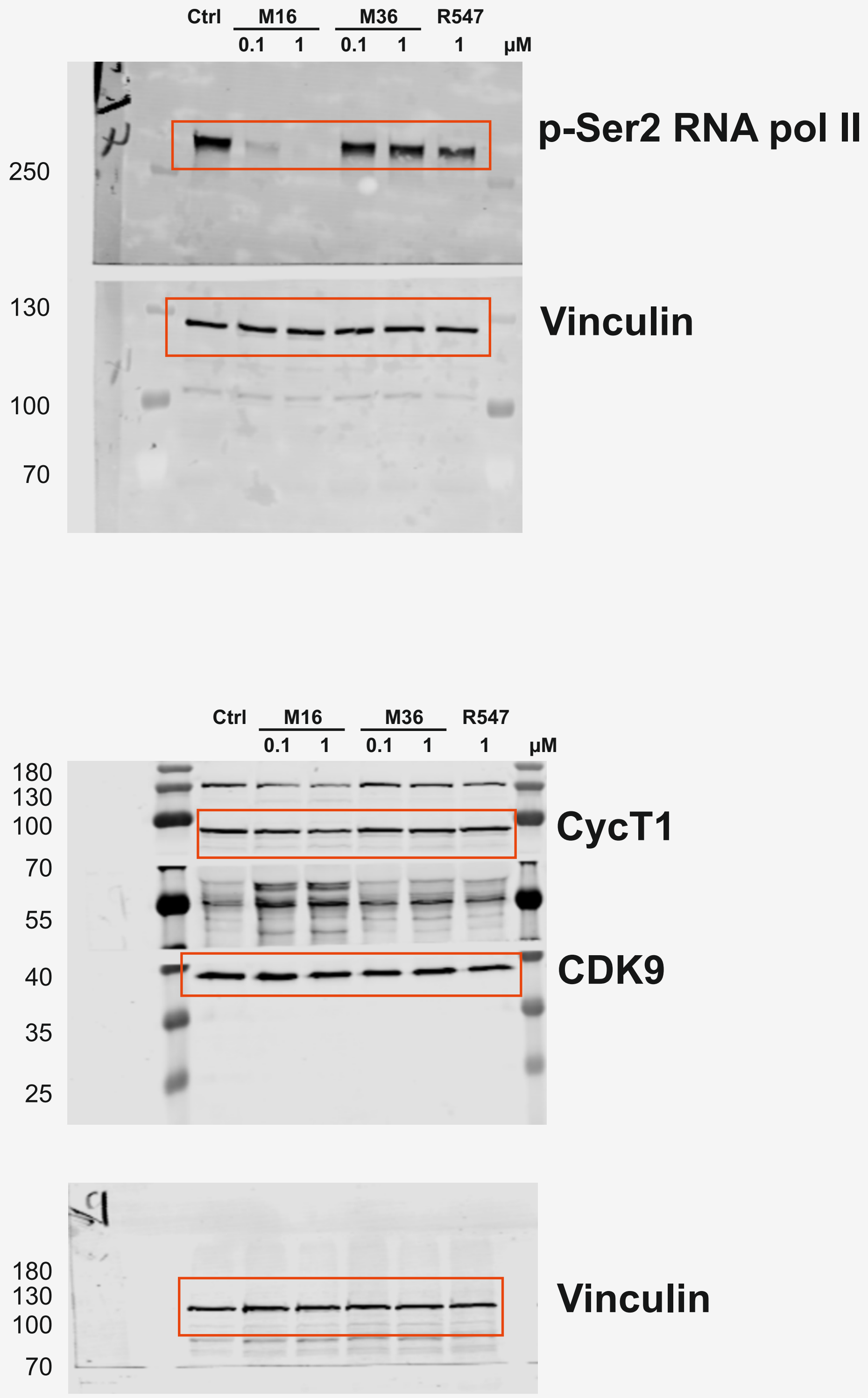

Suppl. Figure 7C

12 h

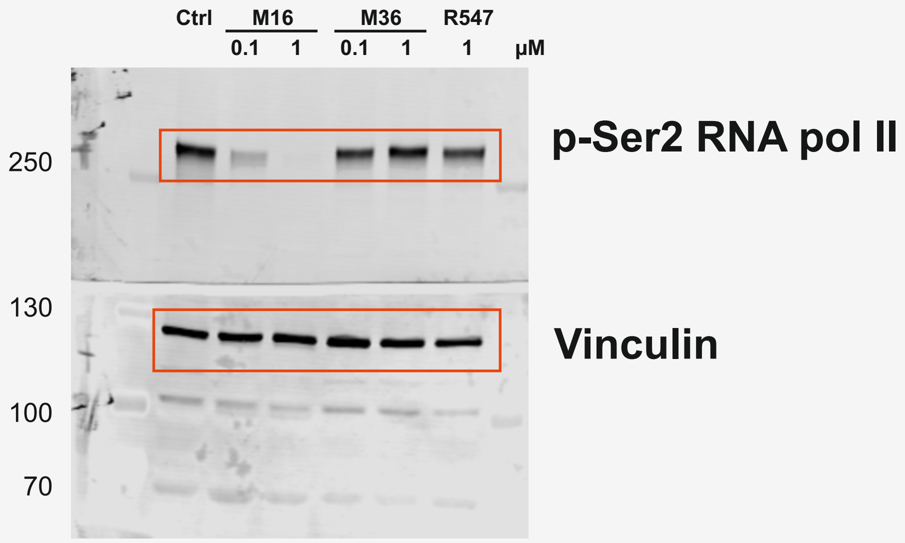

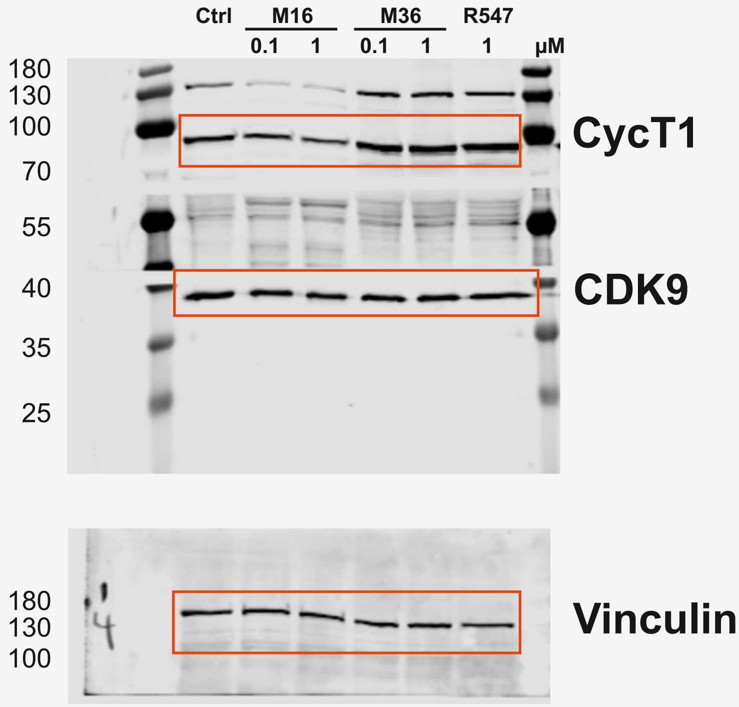

Suppl. Figure 7C

16 h

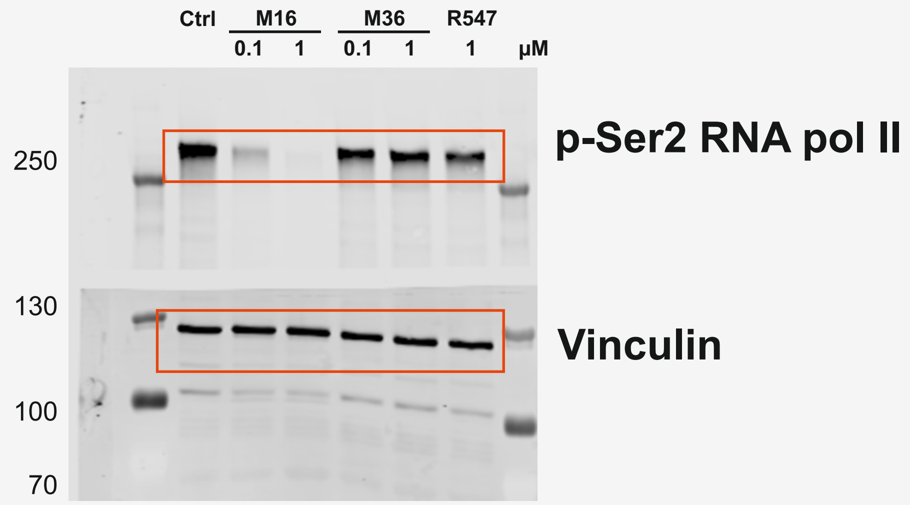

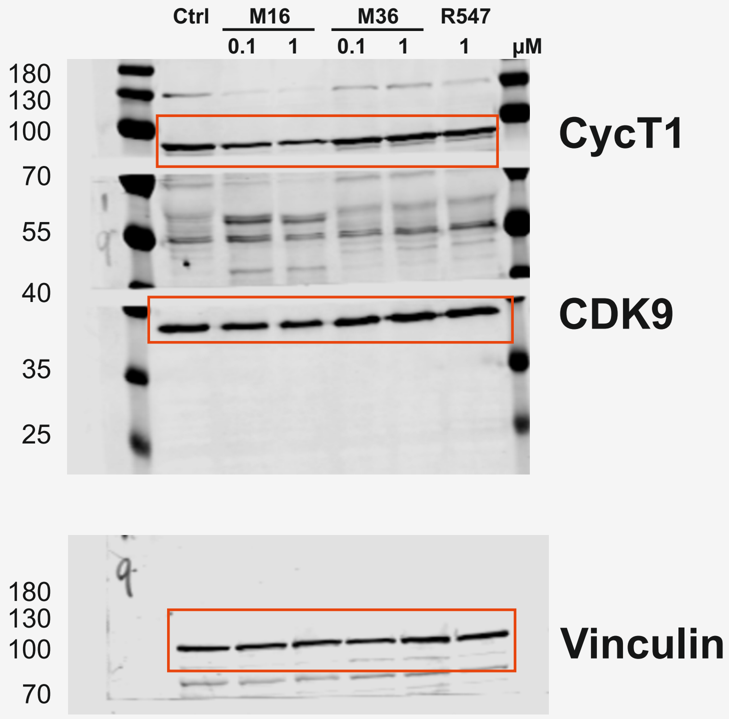

Suppl. Figure 8

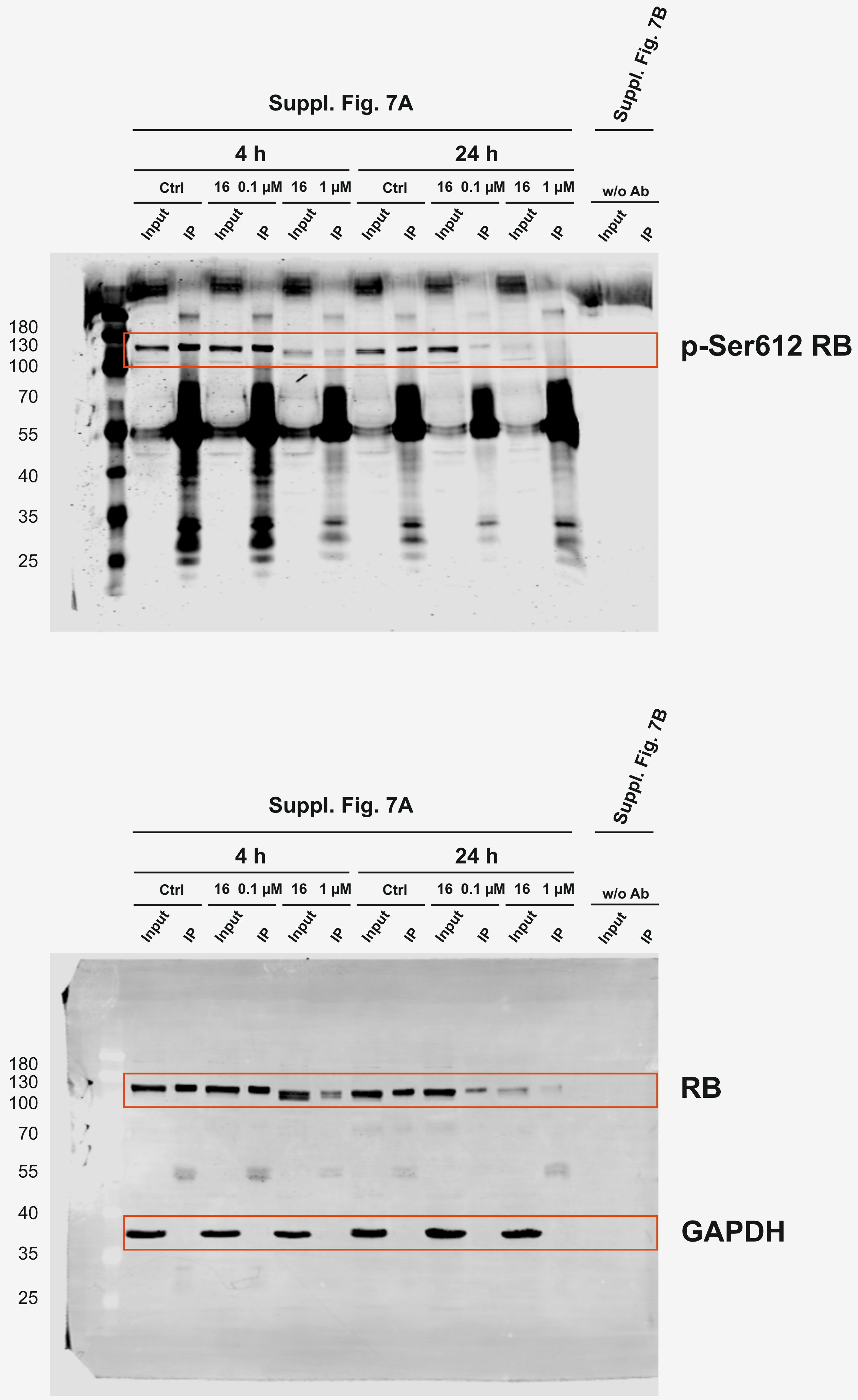

Suppl. Figure 8

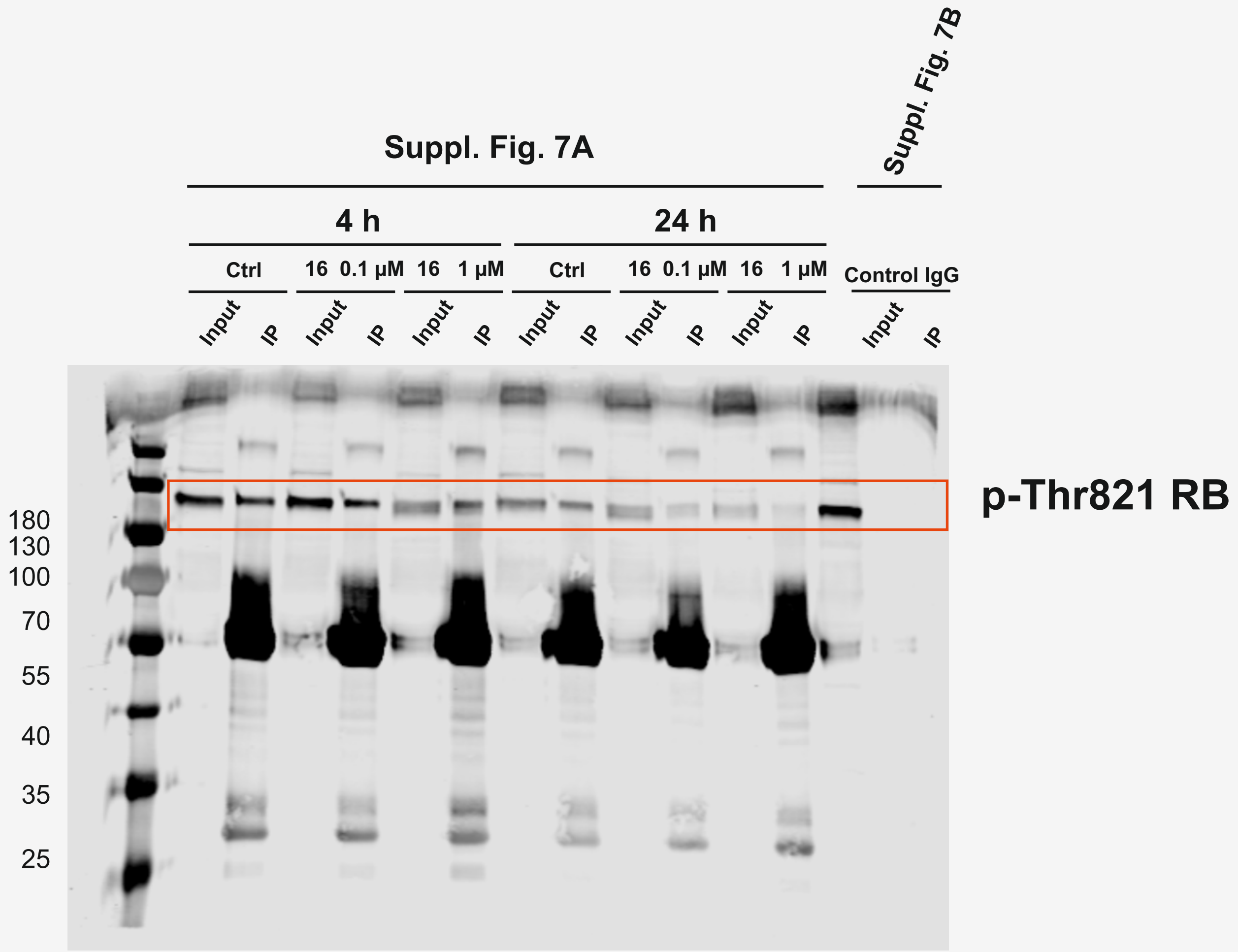

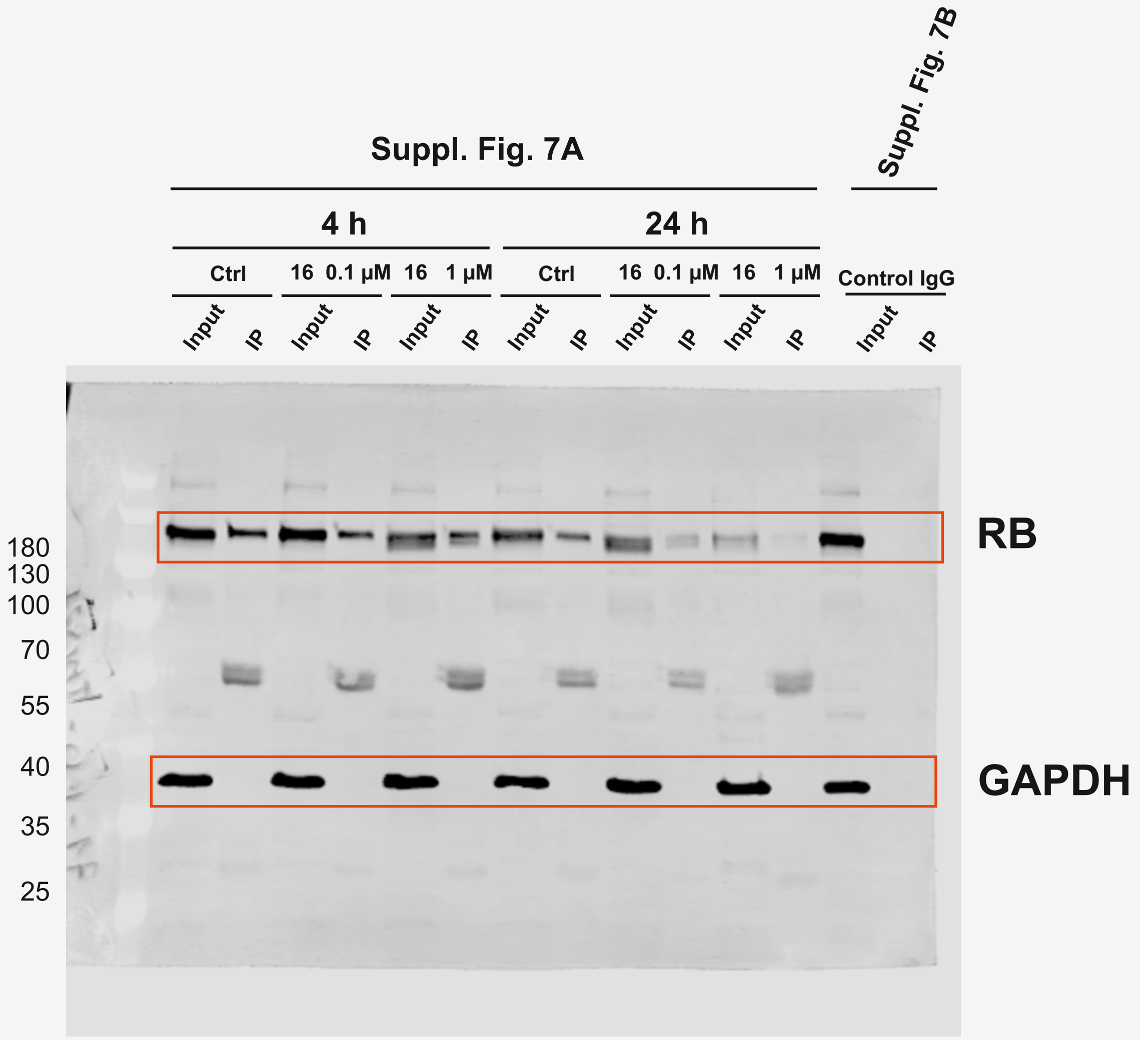

Supplement: Supplementary file 2 — Supplemental Material: Original Western blots [file 41420_2024_2056_MOESM2_ESM.pdf]
